# Supplementary material for: Long-term functional synaptic integration of genome-edited retinal organoids in a primate model of macular degeneration
Source: Mol Ther. 2026 Jun 27;34(9):5444–61. doi: 10.1016/j.ymthe.2026.06.037 (PMC13555564; doi:10.1016/j.ymthe.2026.06.037)
Supplement: Supplementary file 1 [file mmc1.pdf]

YMTHE, Volume *34*

Supplemental Information

Long-term functional synaptic integration of genome-edited retinal organoids in a primate model of macular degeneration

Atsuta Ozaki, Akihiro Kawai, Ryutaro Akiba, Satoko Okayama, Nobuhiko Ohno, Keisuke Kajita, Tomohiro Masuda, Satoshi Yokota, Shin-ichiro Ito, Du Peiyan, Kenta Onoue, Shigenobu Yonemura, Mineo Kondo, Yasuo Kurimoto, Yingbin Fu, and Michiko Mandai

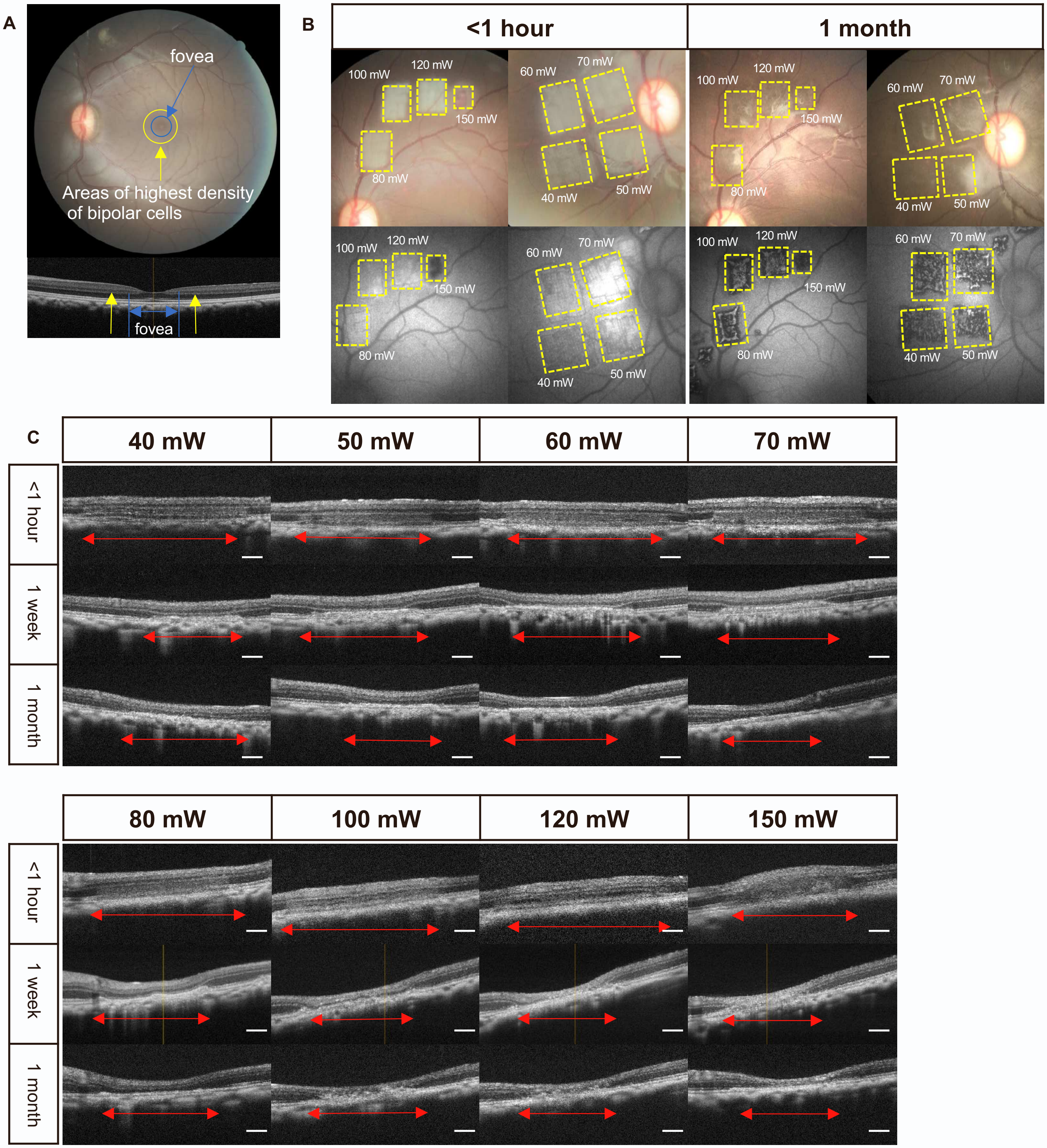

Figure S1. Optimization of laser power from 40 mW to 150 mW. (A) The eccentricity range of fovea is from 0.13 to 0.6 mm (blue circle). The eccentricity of the highest density of BCs are 1mm (yellow circle). (B) Representative fundus images of 80-150 mW (Upper left) and 40-70 mW (Upper right) and autofluorescence images of 80–150 mW (Lower left) and 40–70 mW (Lower right) taken < 1 hour and 1 month after laser. (C) Sectional views of OCT imaging of 40-150 mW < 1 hour, 1 week, and 1 month after laser. The laser area was indicated by red double-headed arrow. Scale bars: 300 μm

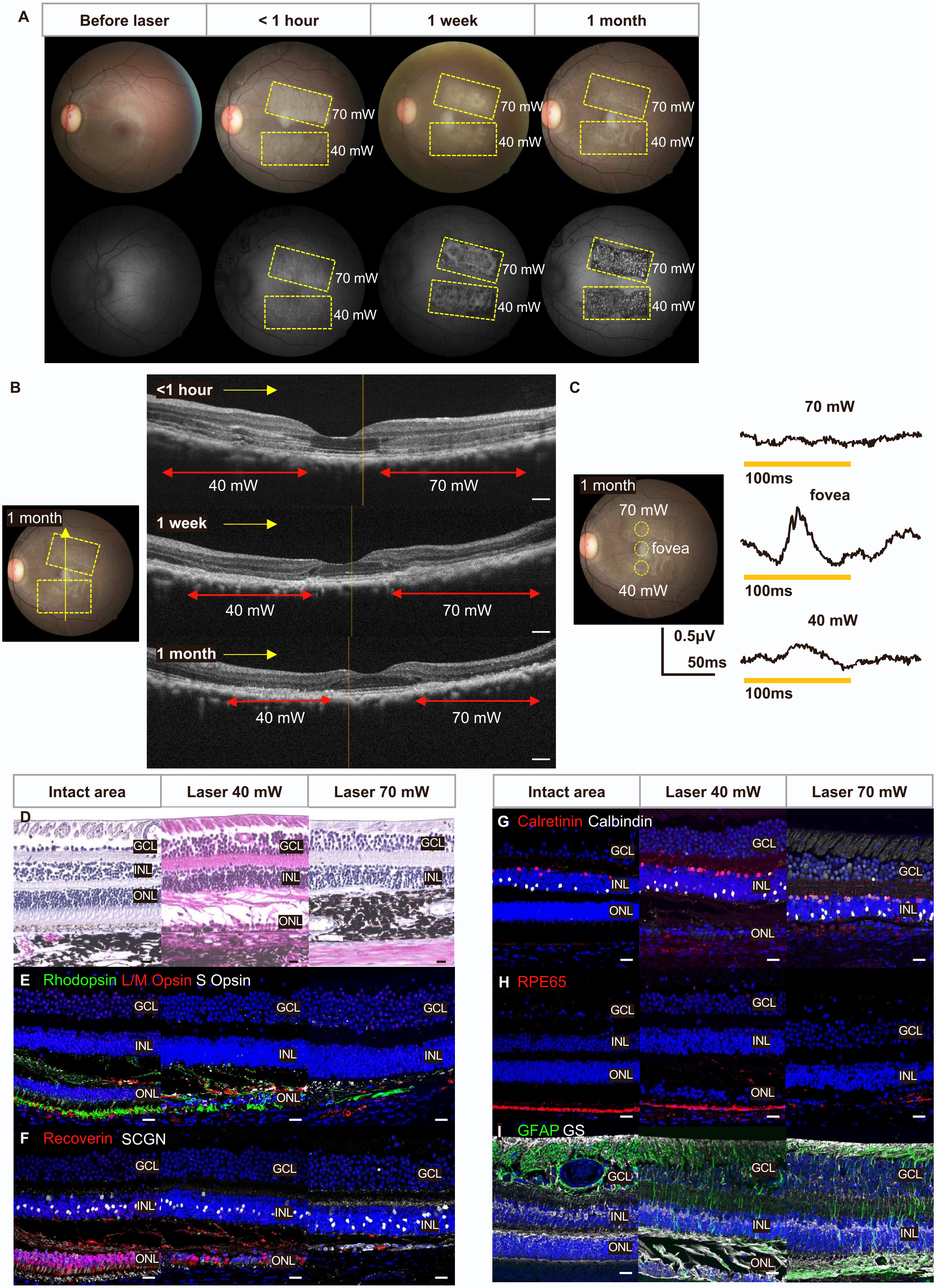

A
Before laser
< 1 hour
1 week
1 month
70 mW
40 mW
B
1 month
<1 hour
1 week
40 mW
70 mW
C
1 month
70 mW
fovea
40 mW
0.5μV
50ms
100ms
D
E Rhodopsin L/M Opsin S Opsin
F Recoverin SCGN
G Calretinin Calbindin
H RPE65
I GFAP GS
Intact area
Laser 40 mW
Laser 70 mW
GCL
INL
ONL

Figure S2. Laser-induced retinal degeneration monkey model involving para- and peri-fovea. (A) Representative fundus images (Upper) and autofluorescence images (Lower) taken before laser treatment, <1 hour, 1 week, and 1 month after laser treatment. The upper area from the fovea was lasered at 70 mW and the lower area at 40 mW (yellow dot). (B) Cross-sectional OCT images of the yellow line on the fundus, taken <1 hour, 1 week, and 1 month after laser treatment. The left side of the OCT was at 40 mW and the right side at 70 mW (red two-headed arrow) Scale bars: 300 µm (C) One month after laser, FMERG was stimulated at 70mW (upper), at the fovea (central), and at 40 mW (lower) (yellow dot circle). (D–I) Histological images of retinas compared with intact area, laser 40 mW, and laser 70 mW. (D) Representative images of Hematoxylin &Eosin stained retinal sections showing ONL loss of Laser 70mW. Scale bars: 20 µm (E–I) Retinal sections stained with Rhodopsin, L/M opsin and S opsin (E), Recoverin and SCGN (F), Calretinin and Calbindin (G), RPE65 (H), GFAP and GS (I). Scale bars: 25 µm

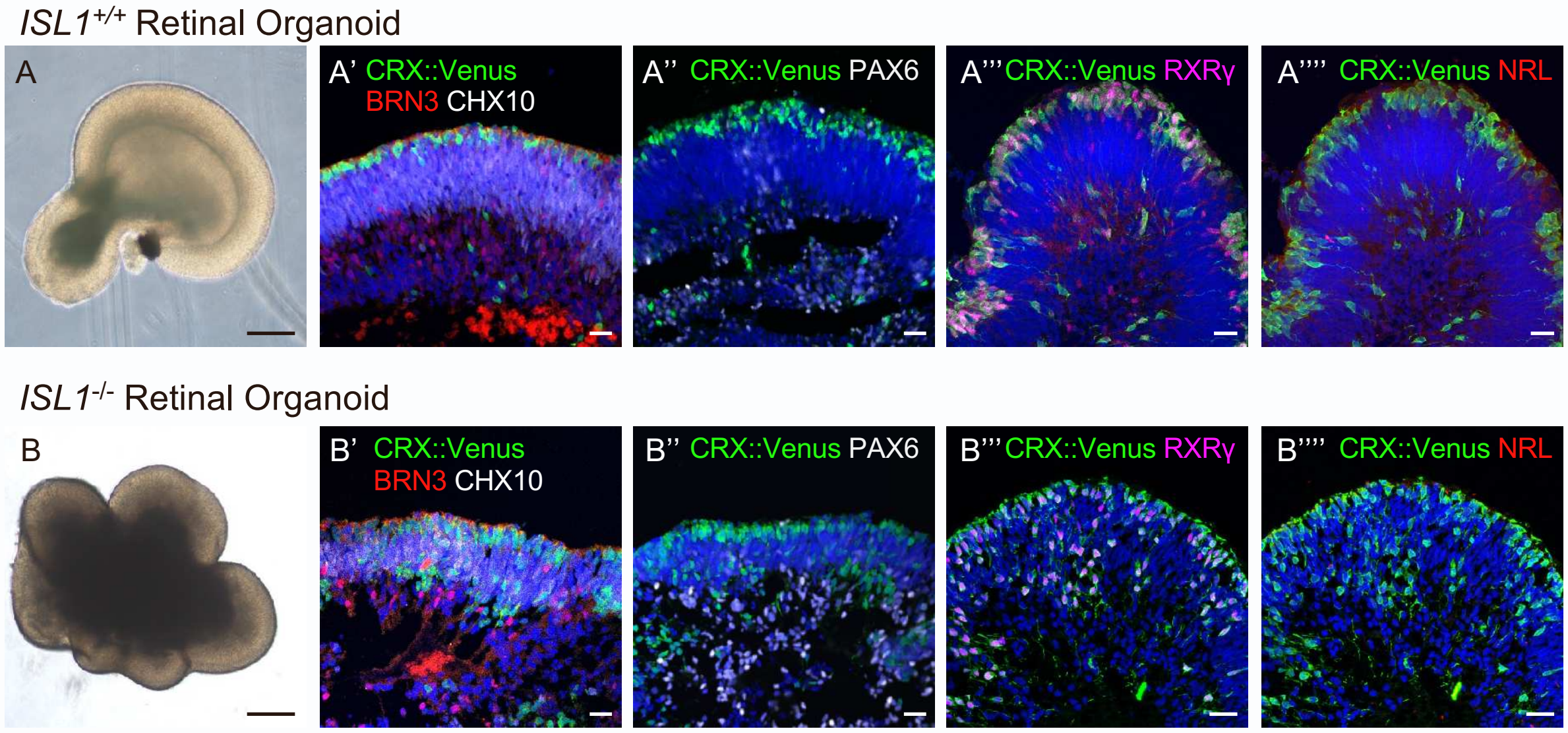

Figure S3. Human ESC-derived RO sheets at the time of transplantation. (A, B) Bright field images of *ISL1*$^{+/+}$ (A) and *ISL1*$^{-/-}$ (B) human ESC ROs (approximately DD60). Scale bars: 100 µm (A’, B’) Immunostaining of *ISL1*$^{+/+}$ (A’) and *ISL1*$^{-/-}$ (B’) human ESC retinal organoids for CRX::Venus, BRN3, and CHX10 (approximately DD60). (A’’, B’’) Immunostaining of *ISL1*$^{+/+}$ (A’’) and *ISL1*$^{-/-}$ (B’’) human ESC retinal organoids for CRX::Venus and PAX6. (A’’’, B’’’) Immunostaining of Immunostaining of *ISL1*$^{+/+}$ (A’’’) and *ISL1*$^{-/-}$ (B’’’) human ESC retinal organoids for CRX::Venus and RXRγ . (A’’’’, B’’’’) Immunostaining of *ISL1*$^{+/+}$ (A’’’’) and *ISL1*$^{-/-}$ (B’’’’) human ESC retinal organoids for CRX and NRL . Scale bars: 20 µm (A’–A’’’’, B’–B’’’’)

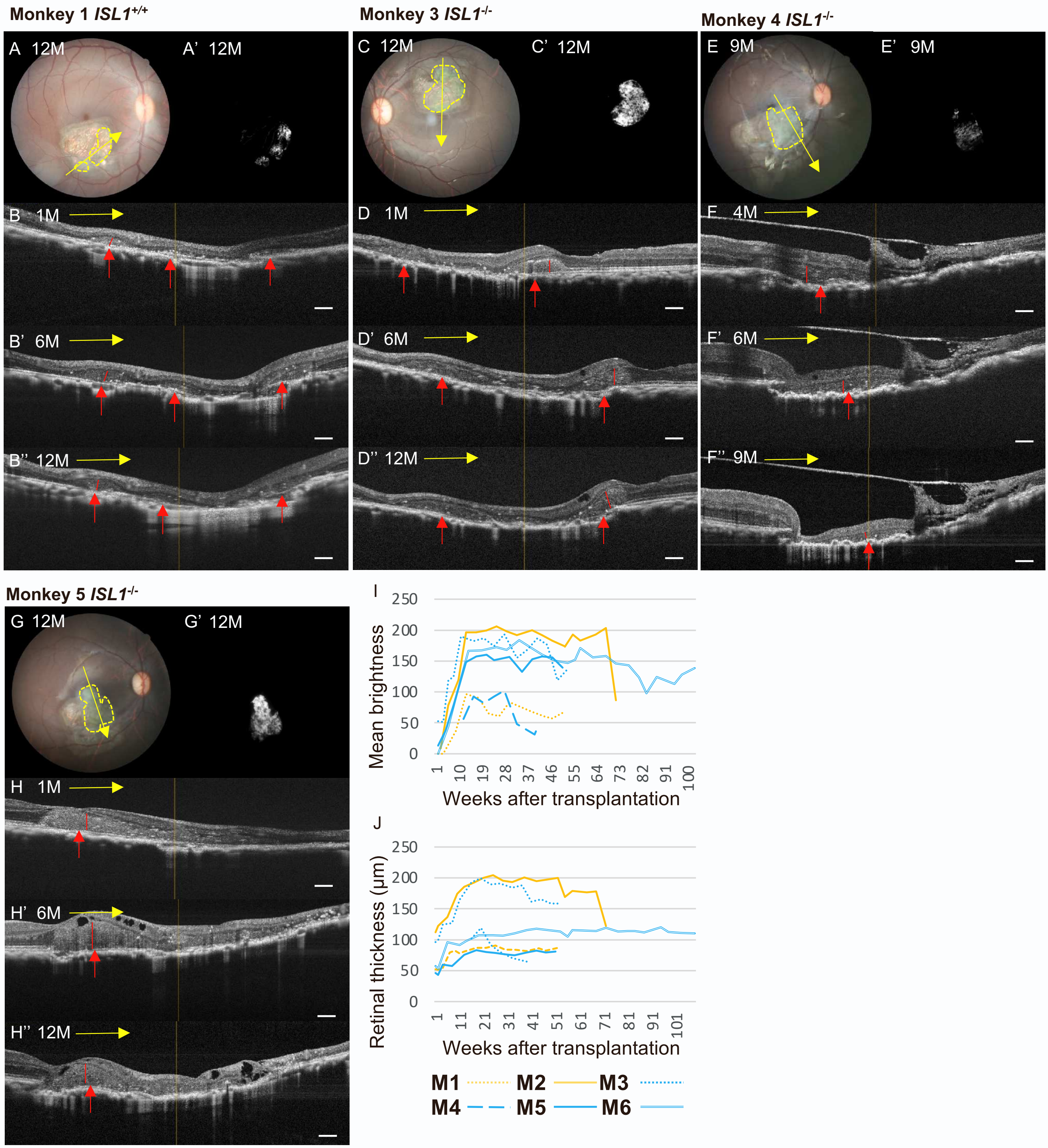

Figure S4. In vivo imaging of human ESC-derived RO sheets after transplantation. (A–H'') Representative fundus images (A, C, E, G), FA images of CRX::Venus (A', C', E', G'), and OCT images (B–B'', D–D'', F–F'', H–H'') of the transplanted grafts in the monkey eyes (Monkey 1, 3, 4, 5, respectively). Scale bars: 300µm (I) Line graph of temporal changes in Mean brightness of CRX::Venus for each transplanted eye. (J) Line graph of temporal changes in graft thickness for each transplanted eye.

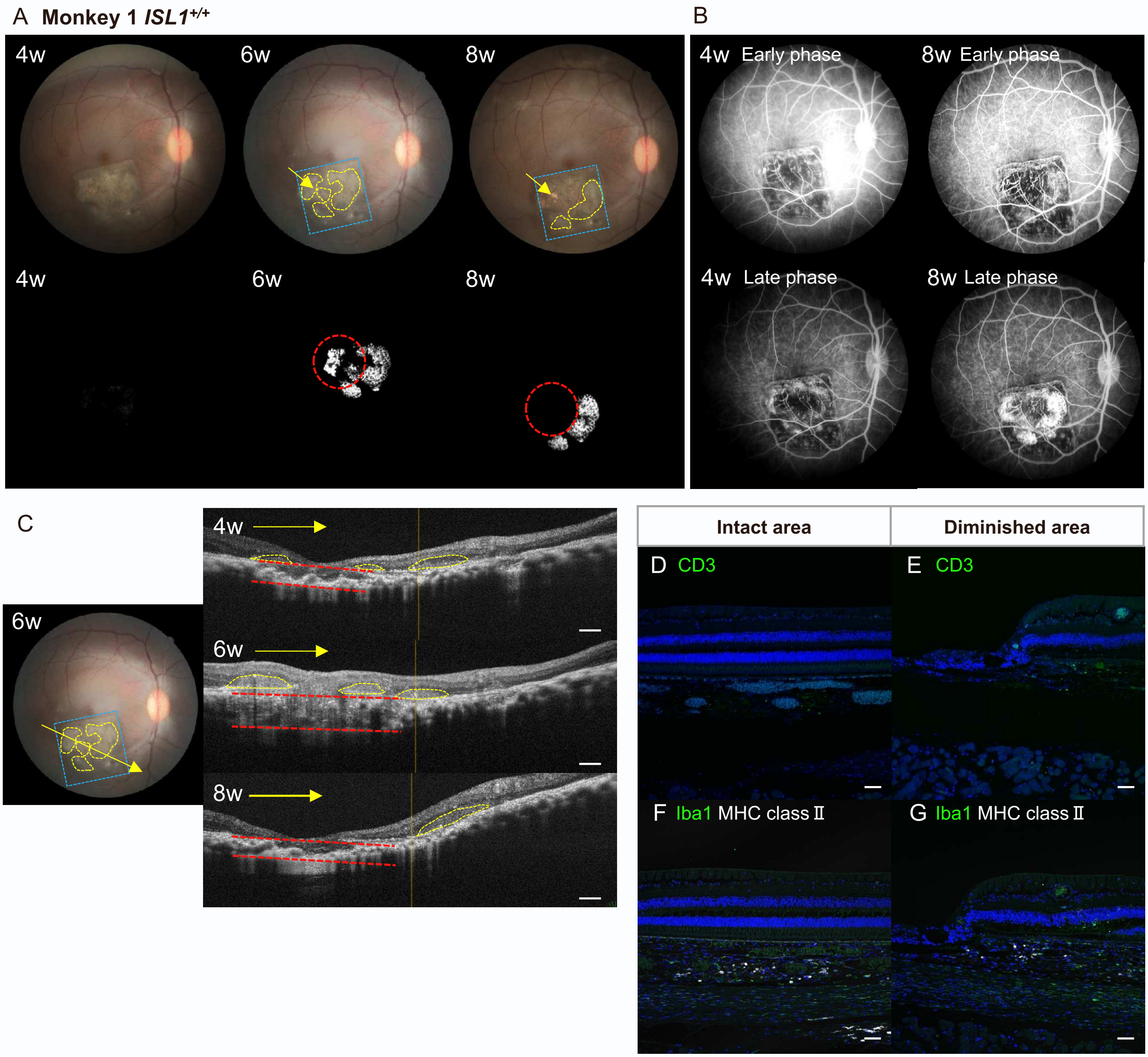

Figure S5. Evaluation of Monkey 1 before and after graft loss. (A) Representative fundus images of the transplanted graft (yellow dot) before and after graft loss (red dot circle). Laser area was indicated by light blue dot, insertion site was indicated by yellow arrow. (B) Representative FA images of the transplanted graft before (4w) and after (8w) graft loss. (C) Representative OCT images of the transplanted graft before (6w) and after (8w) graft loss. The grafts (yellow dot) were located at the laser area (light blue dot) Choroidal thickness was indicated by red dot line. Scale bars: 300μm (D–G) Retinal sections were stained with CD3 (D, E), Iba1 (F, G), and MHC class II (F, G) in the intact area and graft diminished area. Scale bars: 50 μm

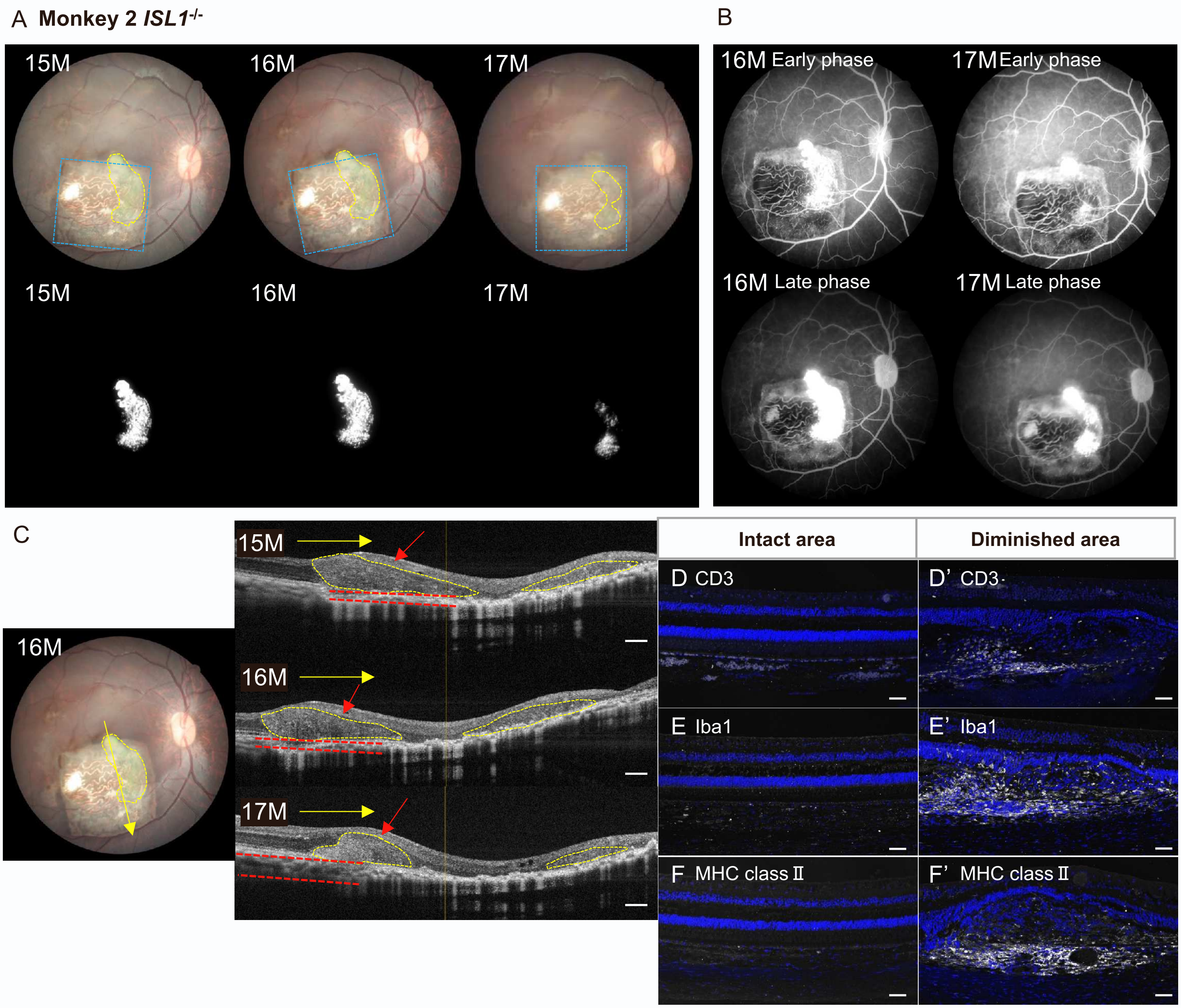

Figure S6. Evaluation of Monkey 2 before and after graft loss. (A) Representative fundus images of the transplanted graft (yellow dot) before and after graft loss. Laser area was indicated by light blue dot. (B) Representative FA images of the transplanted graft before (16M) and after (17M) graft loss. (C) Representative OCT images of the transplanted graft between 15M and 17M. The grafts (yellow dot) were located at the transplanted area, and the diminished area was indicated by red arrow. Red dot line showed the thickness of choroid vessel. Scale bars: 300 μm (D–F') Retinal sections were stained with CD3 (D, D'), Iba1 (E, E'), and MHC class II (F, F') in the intact area and graft diminished area. Scale bars: 50 μm

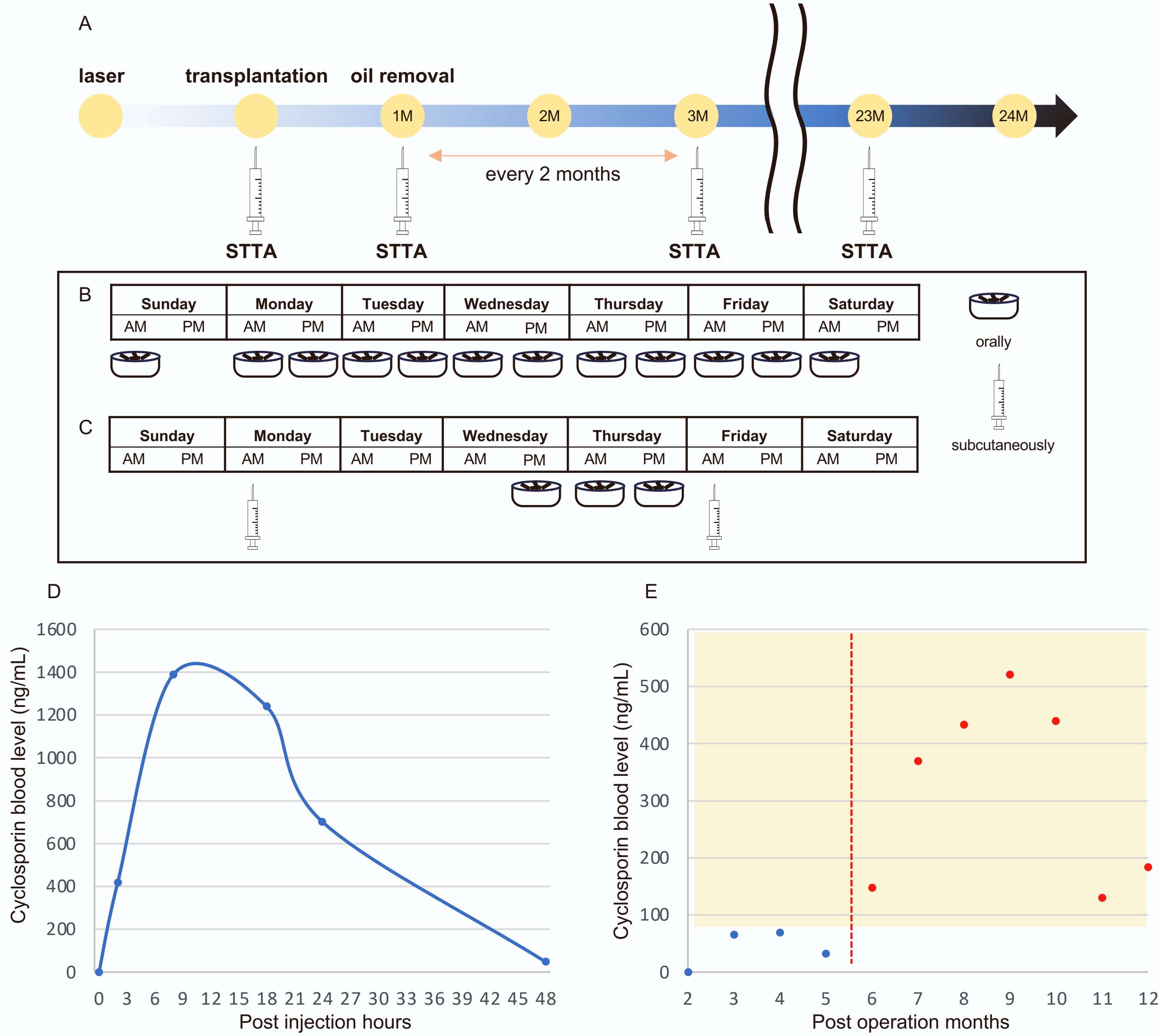

Figure S7. Management of local and systemic immunosuppression. (A) Local immunosuppressant injection protocol. STTA was administered at the time of transplantation and oil removal, and then every 2 months. (B) Systemic immunosuppression protocol of cyclosporin orally. (C) Systemic immunosuppression protocol of oral and subcutaneous administration of cyclosporin (D) Cyclosporine blood concentration profile after subcutaneous administration of cyclosporin. Cyclosporine concentrations were measured over time in four monkeys. (E) Changes in blood concentration of cyclosporine in monkey 3. Blue dots represent cyclosporin managed by only orally, whereas red dots represent cyclosporin managed subcutaneously and orally.

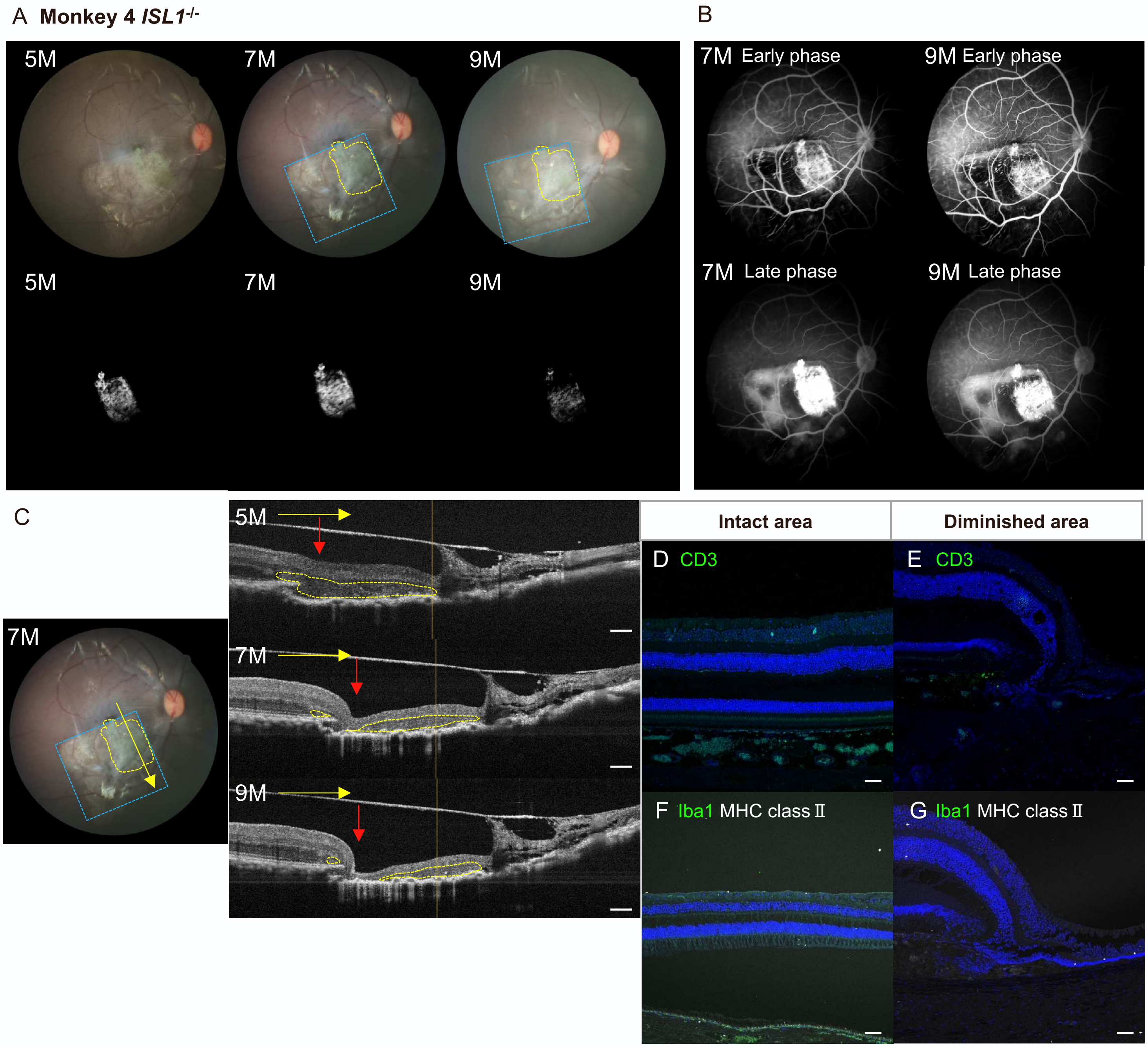

Figure S8. Evaluation of Monkey 4 before and after graft loss. (A) Representative fundus images of the transplanted graft (yellow dot) before and after graft loss. Laser area was indicated by light blue dot. (B) Representative FA images of the transplanted graft before (7M) and after (9M) graft loss. (C) Representative OCT images of the transplanted graft before (7M) and after (9M) graft loss. The grafts (yellow dot) were located at the laser area (light blue dot), and the diminished area was indicated by red arrow. Scale bars: 300 µm (D–G) Retinal sections were stained with CD3 (D, E), Iba1 (F, G), and MHC class II (F, G) in the intact area and diminished area. Scale bars: 50 µm

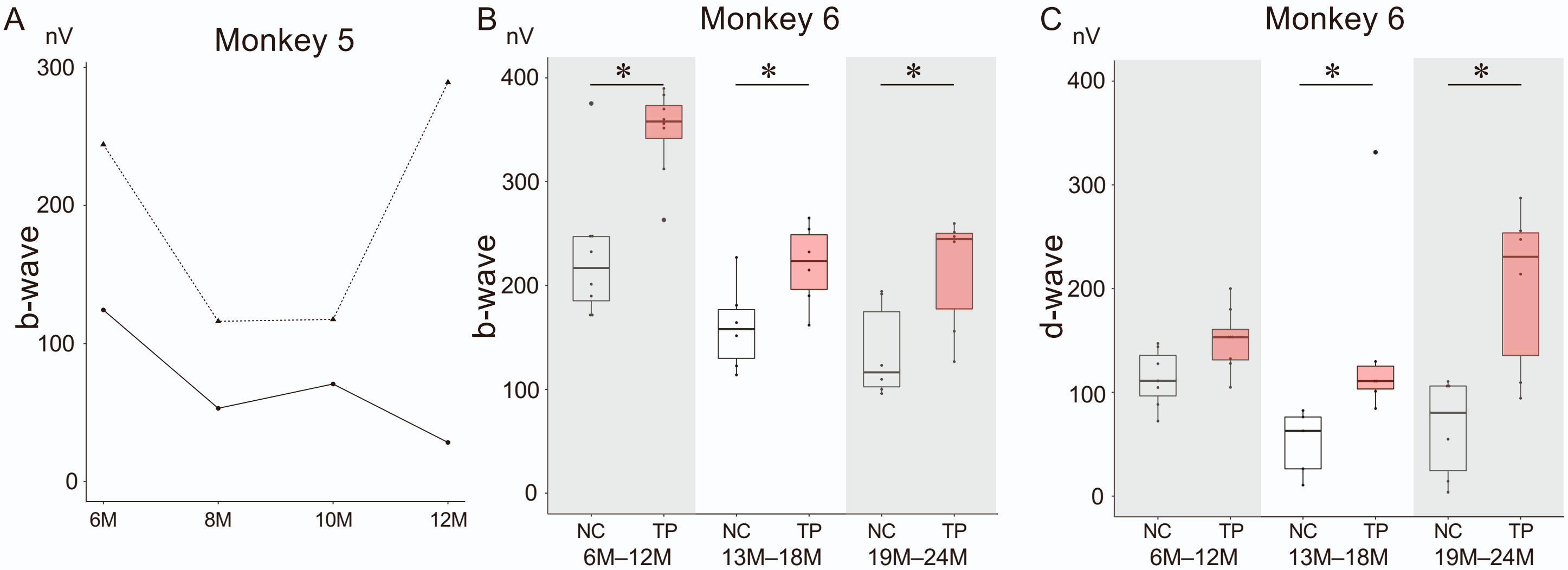

Figure S9. **Longitudinal comparison between grafted and non-grafted area in Monkey 5 and Monkey 6** (A) Longitudinal comparison of FMERG wave amplitudes between grafted and non-grafted retinal areas in Monkey 5. Dotted and solid lines indicate the longitudinal changes in b-wave amplitudes in grafted and non-grafted areas, respectively. (B, C) Comparison of FMERG wave amplitudes between non-grafted (NC) and grafted (TP) areas in Monkey 6 at 6 months–12 months, 13 months–18 months, and 19 months–24 months post transplantation for b-wave (B) and d-wave (C) (*Exact Wilcoxon rank sum test, $p < 0.05$).

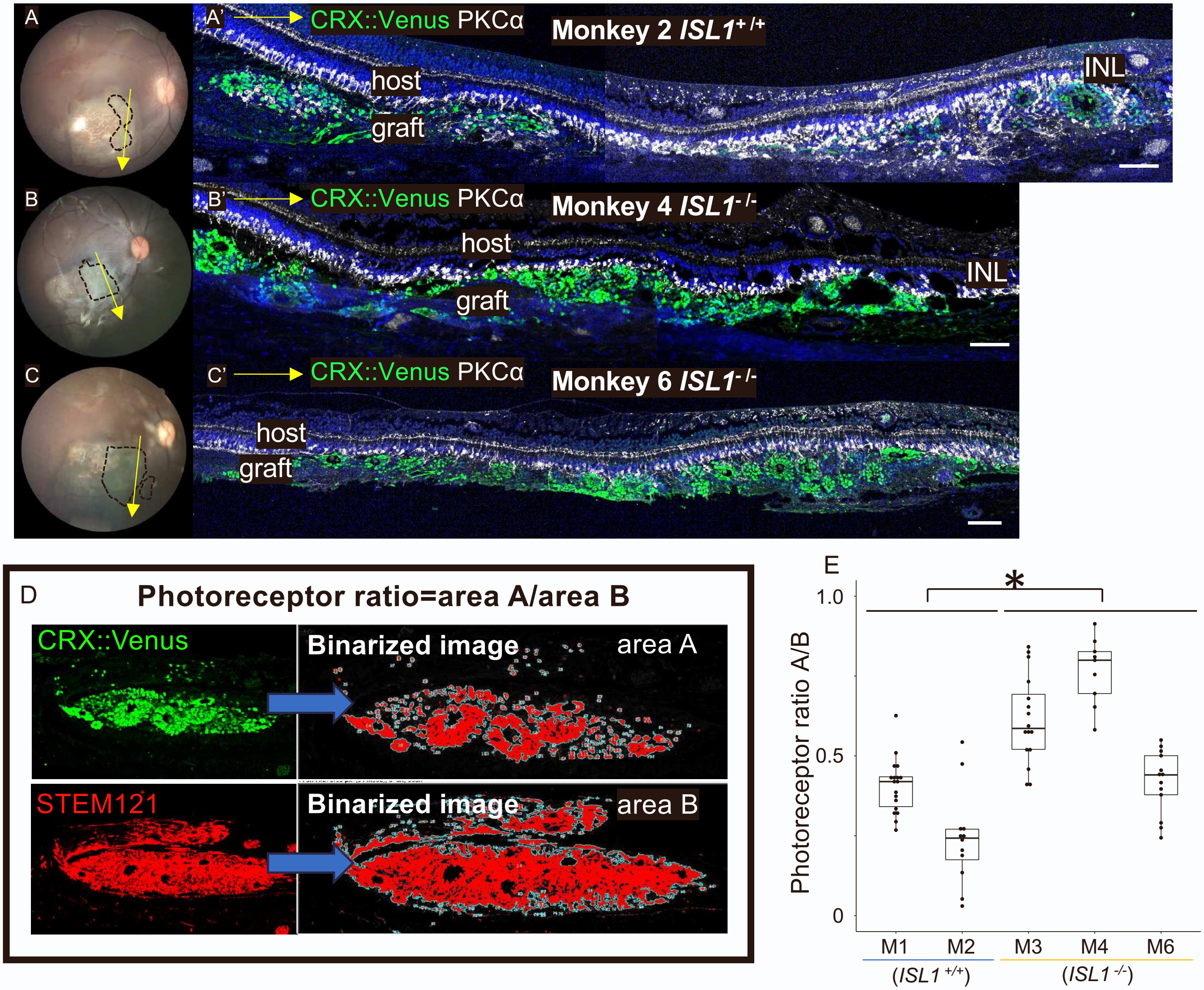

Figure S10. **Histological evaluation of the proportion of photoreceptor cells between *ISL1*$^{+/+}$ and *ISL1*$^{-/-}$.** (A–C') Immunostaining of monkey retinas transplanted with *ISL1*$^{+/+}$ (A) or *ISL1*$^{-/-}$ (B, C) RO sheets at 17 months (A), 9 months (B), and 24 months (C) post-transplantation. Graft photoreceptors were identified within CRX::Venus-positive rosettes in the graft area, and host bipolar cells were defined by their anatomical location within the host INL. In *ISL1*$^{-/-}$ grafts lacking graft-derived PKCα-positive ON-bipolar cells, all PKCα-positive dendrites extending from the host were therefore considered exclusively of host origin. Yellow arrows in the fundus images indicate the orientation of the retinal sections. Scale bars: 100 µm (D) Schematic representations of photoreceptors ratio with binarized images of CRX::Venus (graft photoreceptors) and STEM121 (graft cells). The photoreceptor ratio was defined as the area of CRX::Venus divided by the area of STEM121. (E) The photoreceptor ratio in the grafts of each monkey (monkey 1: 18 sections; monkey 2: 12 sections; monkey 3: 16 sections; monkey 4: 9 sections; monkey 6: 13 sections) (*Exact Wilcoxon rank sum test, $p < 0.05$).

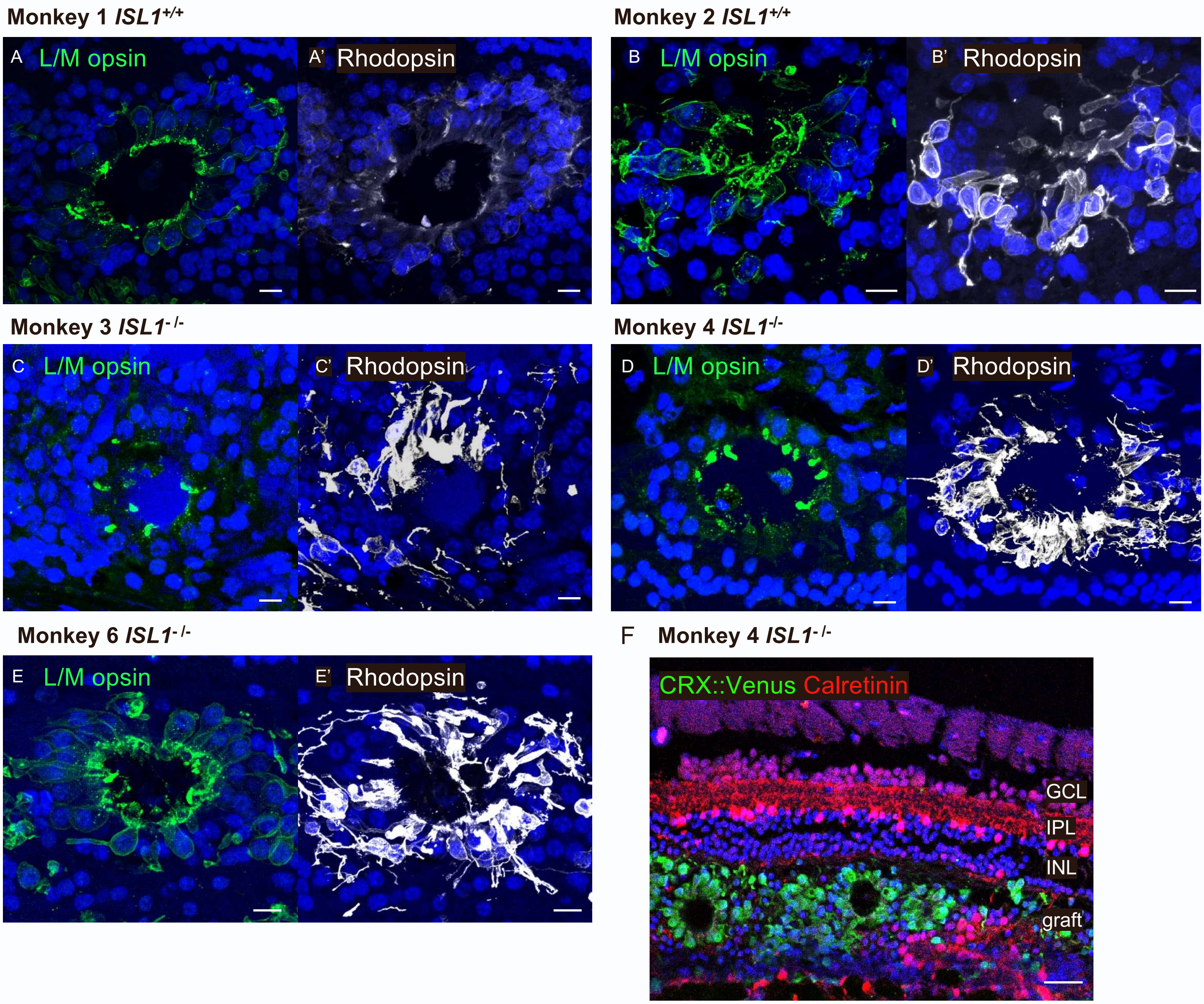

Figure S11. **Histological evaluation of graft maturation and inner plexiform layer integrity.** (A–E') Immunostaining of transplanted monkey retinas showing L/M-opsin and rhodopsin expression in photoreceptor rosettes in *ISL1*$^{+/+}$ and *ISL1*$^{-/-}$ grafts post-transplantation, indicating the differentiation of cone and rod photoreceptors. Scale bars: 10 µm. (F) Representative image of Monkey4 , showing host retinal integrity, as indicated by Calretinin immunopositivity in the host inner plexiform layer in the engrafted area with CRX::Venus-positive graft. Scale bars: 50 µm

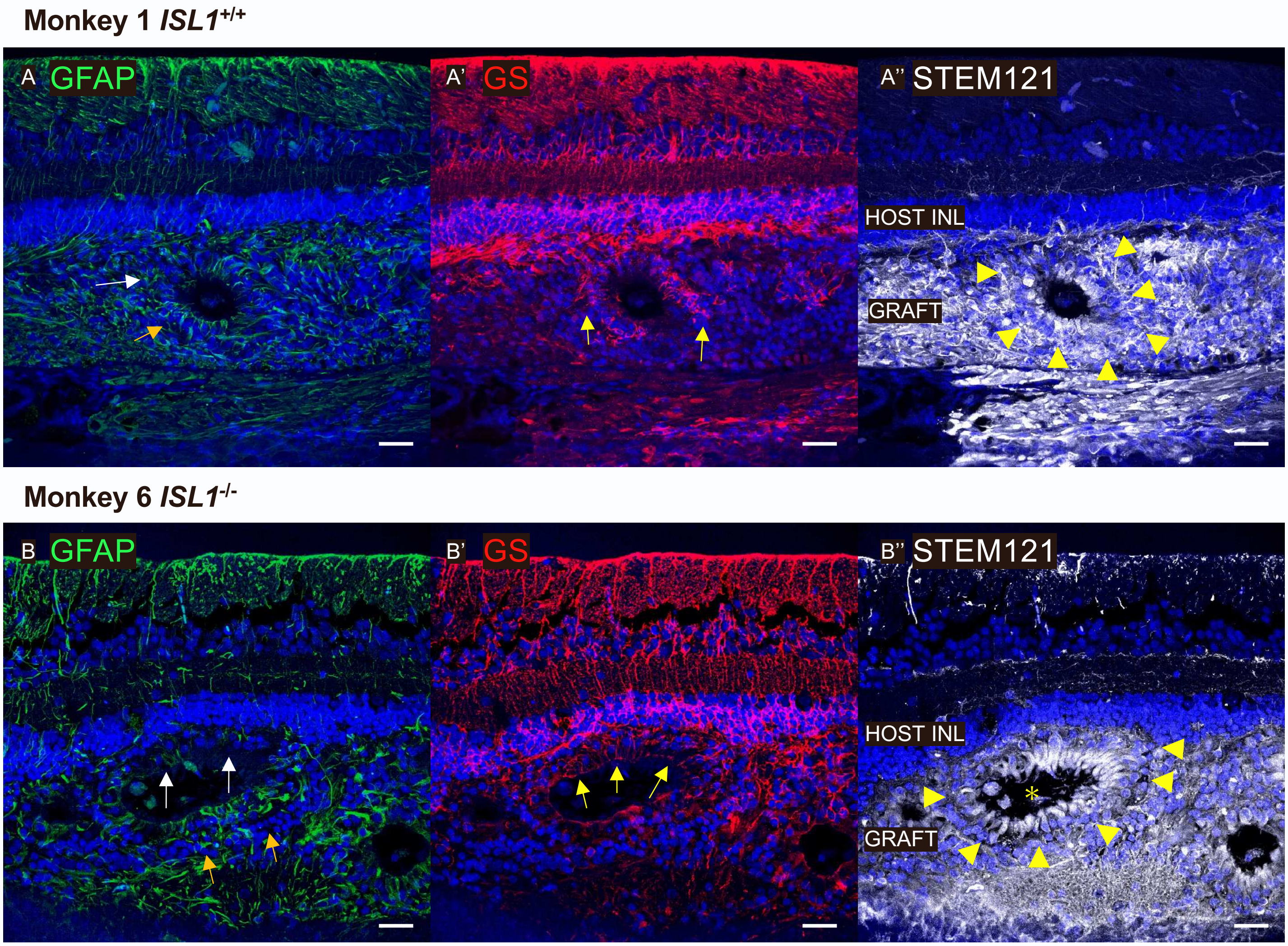

Figure S12. Host and graft Müller cells in *ISL1*$^{+/+}$ and *ISL1*$^{-/-}$ grafts after transplantation. (A–B'') Representative images of host and graft Müller cells from Monkey 1 (*ISL1*$^{+/+}$ grafts; A–A'') and Monkey 6 (*ISL1*$^{-/-}$ graft; B–B''). GS-positive Muller cells were consistently present alongside graft photoreceptors within rosettes, potentially supporting photoreceptor alignment (yellow arrows in A' and B') Scale bars: 30 µm . GFAP was moderately activated in the host retina but did not form a gliotic barrier between the host and graft. Within the graft, GFAP was elevated on the RPE-proximal side of rosettes (orange arrows) compared to the host-proximal side (white arrows in A and B). STEM121 labels grafted ROs, including photoreceptor rosettes (yellow arrow heads in A'' and B'') in which photoreceptors sometimes exhibited inner/outer segment-like morphology (asterisk). GFAP, glial fibrillary acidic protein; GS, glutamine synthetase; RPE, retinal pigment epithelium; INL, inner nuclear layer; ONL, outer nuclear layer. Graft-derived cells were distinguished by STEM121.

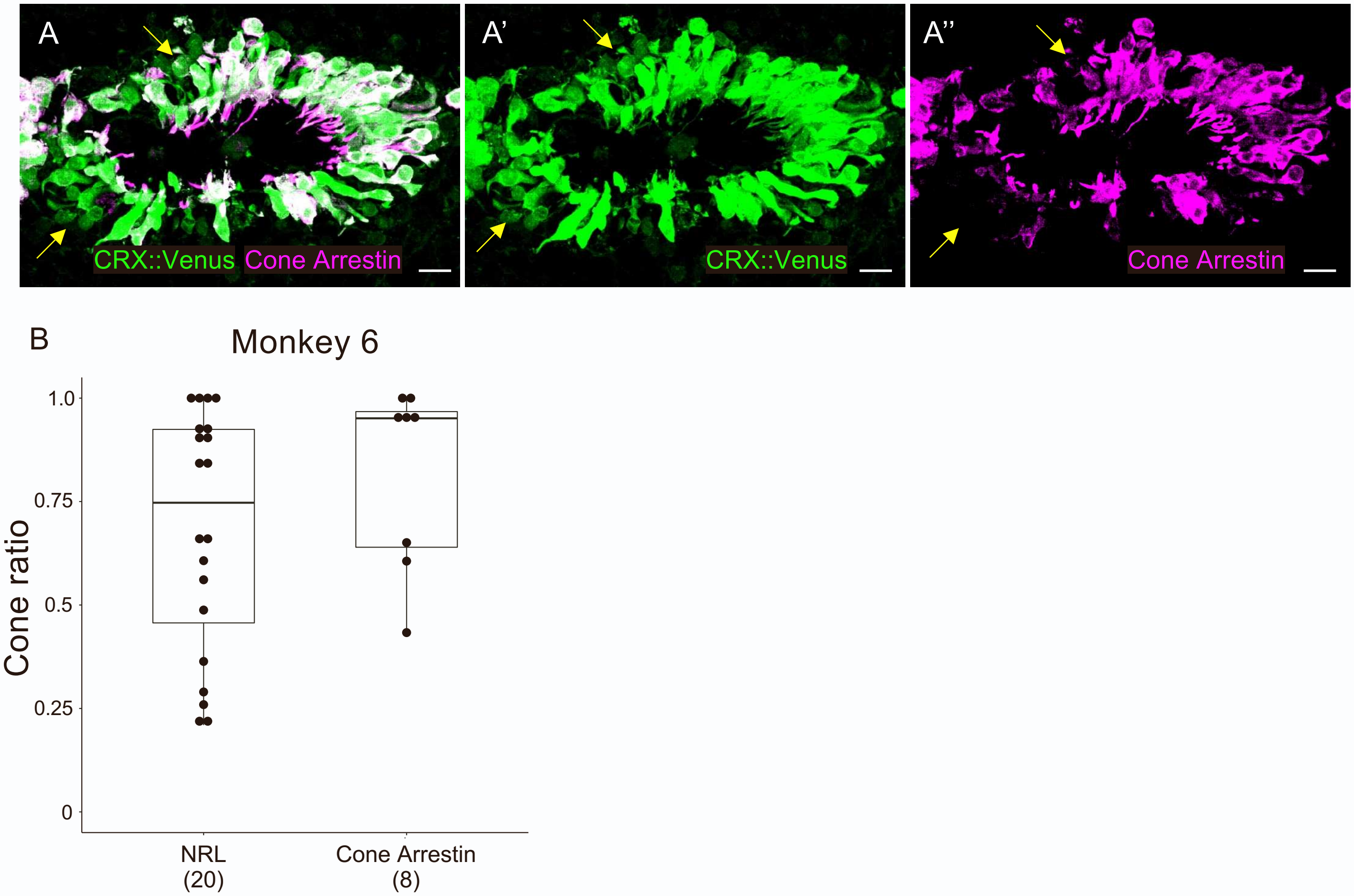

Figure S13. The cone ratio of Monkey 6 using Cone Arrestin. (A–A'') Representative image for CRX::Venus and Cone Arrestin immunostaining in the graft (monkey 6), distinguishing cone from rod photoreceptors. Graft photoreceptors were identified within CRX::Venus-positive rosettes in the graft area. Scale bars: 15 µm (B) Quantification of cone proportion per rosette in monkey 6 using NRL-based (CRX+ NRL-/CRX+) or Cone Arrestin-based (CRX+ Cone Arrestin+/CRX+) classification (NRL: n = 20 rosettes; Cone Arrestin: n = 8 rosettes). Yellow arrows indicate rod photoreceptors (CRX::Venus-positive cells; Cone Arrestin-negative cells)

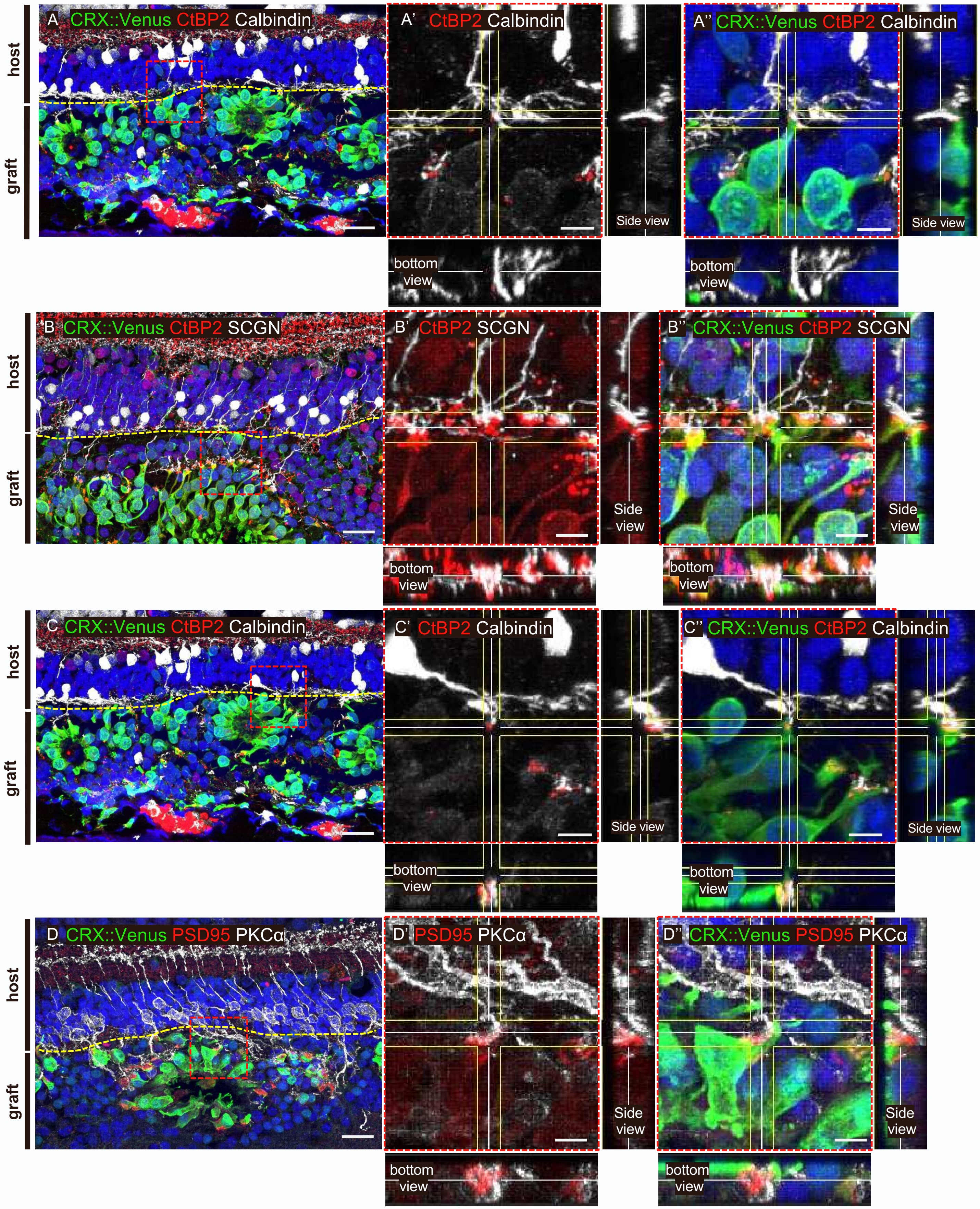

A CRX::Venus CtBP2 Calbindin
host
graft
A' CtBP2 Calbindin
Side view
bottom view
A'' CRX::Venus CtBP2 Calbindin
Side view
bottom view
B CRX::Venus CtBP2 SCGN
host
graft
B' CtBP2 SCGN
Side view
bottom view
B'' CRX::Venus CtBP2 SCGN
Side view
bottom view
C CRX::Venus CtBP2 Calbindin
host
graft
C' CtBP2 Calbindin
Side view
bottom view
C'' CRX::Venus CtBP2 Calbindin
Side view
bottom view
D CRX::Venus PSD95 PKCα
host
graft
D' PSD95 PKCα
Side view
bottom view
D'' CRX::Venus PSD95 PKCα
Side view
bottom view

Figure S14. Host-graft synaptic formation of transplanted photoreceptors.
(A–B'') Presynaptic marker CtBP2 was localized on the margin of the CRX::Venus (graft photoreceptors) and the dendrite tips of Calbindin-positive (A–A'') and SCGN-positive (B-B'') BCs. Scale bars: 15 µm (A, B), 4 µm (A', A'', B, B'') (C–C'') Presynaptic marker CtBP2 was localized on the margin of the CRX::Venus (graft photoreceptors) and the dendrite tips of Calbindin-positive horizontal cells. Scale bars: 15 µm (C), 4 µm (C', C'') (D–D'') Presynaptic marker PSD95 was localized on the margin of graft photoreceptors identified within CRX::Venus-positive rosettes in the graft area, contacting the dendritic tips of host PKCα-positive BCs, identified by their anatomical location within the host INL. PKCα-positive bipolar dendrites were considered exclusively of host origin, as *ISL1*$^{-/-}$ grafts lack ON-bipolar cells. Scale bars: 15 µm (D), 4 µm (D', D'')

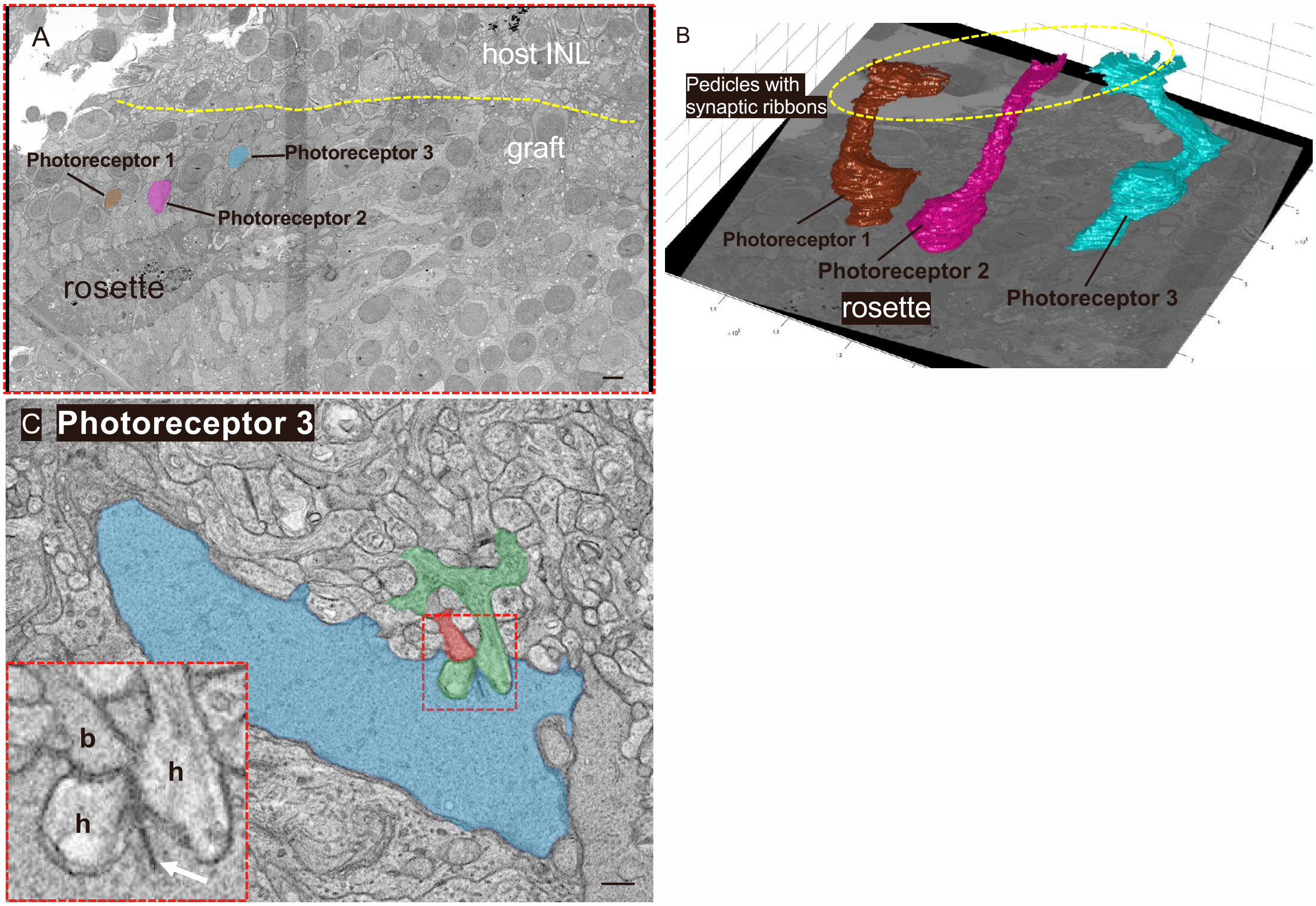

Figure S15. Synaptic connections between host BCs and graft photoreceptors observed in EM. (A) Enlarged view of the area within the red dotted rectangle in (Figure 7C), showing the rosette that includes each traced photoreceptor. Graft photoreceptors were identified within CRX::Venus-positive rosettes by first acquiring z-stack fluorescence images from an original section, from which serial electron microscopy images were obtained with subsequent correlation of the two imaging modalities. Photoreceptor 3: blue (C) scale bars: 5 μm (B) 3D reconstructed image of graft photoreceptors extending from the rosette into the host INL; three photoreceptors were traced. (C) EM images of the axon terminals of individual photoreceptors. white arrows showed synapse ribbon. b, bipolar cell; h, likely horizontal cell. scale bars: 500 nm

Table S1. Summary of transplanted monkey eyes						
**Monkey number**	**Monkey 1**	**Monkey 2**	**Monkey 3**	**Monkey 4**	**Monkey 5**	**Monkey 6**
**Age at transplantation (year)**	6	3	3	3	3	9
**Transplanted eye**	Right	Right	Left	Right	Right	Right
**Organoid genotype**	Wild type	Wild type	$ISL1^{-/-}$	$ISL1^{-/-}$	$ISL1^{-/-}$	$ISL1^{-/-}$
**Differentiation day of RO at transplantation**	56	63	63	59	63	64
**Days post-laser at transplantation**	29	43	18	43	47	28
**Postsurgical events**	Rejection (resolved with steroid at 1.5 months)	Rejection (17 months)	-	Postoperative Endophthalmitis (3 weeks)	Cataract (1 months)	-
**Graft loss**	+	+	-	+	-	-
**Sampling time for histology**	14 months (427 days)	17 months (509 days)	14 months (413 days)	9 months (286 days)	13 months (402 days)	25 months (769 days)

Table S2. Immunohistochemical summary of bipolar cells

**Primary Antibody**	**Type of Bipolar cells**				
**EAAT2**	**Diffuse Bipolar 1**	**Diffuse Bipolar 2**	**Diffuse Bipolar 3a**	**Diffuse Bipolar 3b**	**Flat midget Bipolar**
**Calbindin**	**Diffuse Bipolar 3a**	<u>Diffuse Bipolar 6</u>	Horizontal cell		
**SCGN**	**Diffuse Bipolar 1**	<u>Diffuse Bipolar 6</u>			
**PKC** α	<u>Diffuse Bipolar 4</u>	<u>Diffuse Bipolar 5</u>	<u>Rod bipolar</u>		

ON-bipolar cells are indicated by solid lines, whereas OFF-bipolar cells are indicated in bold. Cell-type markers for the primary antibodies were selected based on previous studies.[19,22]

Table S3. Summary of primary antibodies		
Goat polyclonal anti-BRN3	Santa Cruz biotechnology	Cat#sc-6026 RRID: AB_673441
Rabbit polyclonal anti-Calbindin	Millipore	Cat#ABN2192 RRID: AB_2935805
Mouse monoclonal anti-Calretinin	Millipore	Cat#MAB1568 RRID: AB_94259
Rabbit polyclonal anti-CD3	Abcam	Cat#ab5690 RRID: AB_305055
Mouse monoclonal anti-CHX10	Santa Cruz biotechnology	Cat#sc-365519 RRID: AB_10842442
Mouse monoclonal anti-Arrestin C	Sigma-Aldrich	Cat#MABN2636 RRID:AB_2935804
Mouse monoclonal anti-CtBP2	BD	Cat#612044 RRID: AB_399431
Rabbit polyclonal anti-EAAT2	Proteintech	Cat#22515-1-AP RRID: AB_2879112
Rabbit polyclonal anti-GFAP	DAKO	Cat#Z0334 RRID: AB_ 10013382
Rabbit polyclonal anti-GFP	Abcam	Cat#ab290 RRID: AB_ 2313768
Goat polyclonal anti-Venus	ORIGENE	Cat#AB2166-100 RRID: AB_2895457
Mouse monoclonal anti-GluK1	Santa Cruz biotechnology	Cat#sc-393420 RRID: AB_2716684
Mouse monoclonal anti-GS	Millipore	Cat#MAB302 RRID: AB_ 2110656
Rabbit polyclonal Anti-GNG13	Abcam	Cat#ab126562 RRID:
Rabbit polyclonal anti-Iba1	Wako	Cat#019-19741 RRID: AB_839504
Rabbit polyclonal anti-mGluR6	Novus Biologicals	Cat#NLS4655 RRID: AB_343723
Mouse Monoclonal anti-MHC classII	DAKO	Cat#M0775 RRID: AB_2313661

Goat polyclonal anti-NRL	R&D	Cat#AF2945 RRID: AB_2155098
Goat polyclonal anti-OPN1SW	Santa Cruz biotechnology	Cat#sc-14363 RRID: AB_2158332
Rabbit polyclonal anti-Opsin Re+G	Sigma-Aldrich	Cat#AB5405 RRID: AB_177456
Rabbit polyclonal anti-Opsin Blue	Millipore	Cat#AB5407 RRID: AB_304867
Mouse monoclonal anti-PAX6	BD Biosciences	Cat#BD561462 RRID: AB_10715442
Rabbit polyclonal anti-Peripherin-2	Proteintech	Cat#18109-1-AP RRID: AB_10665364
Mouse monoclonal anti-PKCα	Sigma-Aldrich	Cat#P5704 RRID: AB_477375
Mouse monoclonal anti-PSD95	Biolegend	Cat#810401 RRID: AB_2564750
Rabbit polyclonal anti-Recoverin	Proteintech	Cat#10073-1-AP RRID: AB_2178005 _
Mouse monoclonal anti-Rhodopsin	Sigma-Aldrich	Cat#O4886 RRID: AB_260838
Rabbit polyclonal anti-RPE65	Takara	Cat#AS2352
Mouse monoclonal Anti-RXR γ	Santa Cruz biotechnology	Cat#sc-365252 RRID: AB_10850062
Sheep polyclonal anti-SCGN	BioVendor R&D	Cat#RD184120100 RRID: AB_2034062
Mouse monoclonal anti-STEM121	Takara	Cat#Y40410 RRID: AB_ 2801314

Video S1. 3D reconstruction of host-graft synaptic connection by SBFSEM. This video shows a host ON-bipolar cell (yellow) and a host OFF-bipolar cell (orange) forming synaptic contacts with a grafted cone photoreceptor (pink).
