# Supplementary material for: Long-term functional synaptic integration of genome-edited retinal organoids in a primate model of macular degeneration
Source: Mol Ther. 2026 Jun 27;34(9):5444–61. doi: 10.1016/j.ymthe.2026.06.037 (PMC13555564; doi:10.1016/j.ymthe.2026.06.037)
Supplement: Supplementary file 2 [file mmc3.pdf]

Molecular Therapy
Original Article

Long-term functional synaptic integration of genome-edited retinal organoids in a primate model of macular degeneration

Atsuta Ozaki,[1,2,3] Akihiro Kawai,[4] Ryutaro Akiba,[4] Satoko Okayama,[5] Nobuhiko Ohno,[6,7] Keisuke Kajita,[1,8] Tomohiro Masuda,[1] Satoshi Yokota,[1,9] Shin-ichiro Ito,[1,9] Du Peiyan,[4] Kenta Onoue,[5] Shigenobu Yonemura,[5,10] Mineo Kondo,[3] Yasuo Kurimoto,[1,9] Yingbin Fu,[11] and Michiko Mandai[1,9]

[1]Kobe City Eye Hospital, Kobe 650-0047, Japan; [2]Cell and Gene Therapy in Ophthalmology Laboratory, BZP, RIKEN, Wako, Saitama 351-0198, Japan; [3]Department of Ophthalmology, Mie University Graduate School of Medicine, Tsu 514-8507, Japan; [4]Ophthalmology and Visual Science, Chiba University Graduate School of Medicine, Chiba 260-8670, Japan; [5]Laboratory for Ultrastructural Research, RIKEN Center for Biosystems Dynamics Research, Kobe 650-0047, Japan; [6]Division of Ultrastructural Research, National Institute for Physiological Sciences, Okazaki 444-8787, Japan; [7]Department of Anatomy, Division of Histology and Cell Biology, Jichi Medical University, Shimotsuke 329-0498, Japan; [8]Department of Ophthalmology, Institute of Biomedical Sciences, Tokushima University Graduate School, Tokushima 770-8503, Japan; [9]Laboratory for Animal Resources and Genetic Engineering, RIKEN Center for Biosystems Dynamics Research, Kobe 650-0047, Japan; [10]Department of Cell Biology, Tokushima University Graduate School of Medicine, Tokushima 770-8503, Japan; [11]Cullen Eye Institute, Baylor College of Medicine, Houston, TX 77030, USA

Retinal organoids represent a promising regenerative strategy for restoring vision in retinal degenerative diseases, but the capacity of host cone bipolar cells in the primate macula to rewire with transplanted photoreceptors has not been established. In this study, we transplanted genome-edited *ISL1*$^{-/-}$ human retinal organoids lacking ON-bipolar cells into an acute laser-induced macular photoreceptor ablation non-human primate model. Using immunohistochemistry, ultrastructural imaging, and focal macular electroretinography, we demonstrate that host rod and cone bipolar cells actively extend dendrites toward grafted photoreceptors and form synaptic contacts, with evidence of functional signal transmission in a subset of transplanted eyes. Longitudinal, per-eye analyses revealed that host ON-bipolar responses improved in two of four eyes with *ISL1*$^{-/-}$ graft by up to 21.6% and remained stable for up to 2 years post transplantation. Moreover, OFF-pathway connectivity showed potential progressive maturation, with delayed increase in d-wave after 13 months in one of those eyes. These findings provide the first demonstration of long-term anatomical host-graft synaptic integration in the primate macula, establishing that central cone bipolar circuits retain the capacity for durable rewiring with human stem-cell-derived grafts. Our results highlight *ISL1*$^{-/-}$ retinal organoids as a promising approach for central vision restoration in macular degeneration.

INTRODUCTION

Inherited retinal dystrophies (IRDs) and age-related macular degeneration cause irreversible vision loss through progressive photoreceptor degeneration.[1,2] Loss of photoreceptors in the macula directly impairs central vision and profoundly affects quality of life. Stem-cell-derived retinal organoids (ROs) represent a promising regenerative strategy,[3–5] but the true restoration of vision depends on whether host bipolar cells (BCs) can re-establish synaptic connections with transplanted photoreceptors. In the retina, photoreceptors (rod and cone photoreceptors) convert light into electrical signals that are relayed via BCs to retinal ganglion cells and ultimately to the brain. ON-BCs depolarize at light onset, while OFF-BCs depolarize at light offset; together, these pathways encode the contrast and temporal information essential for visual perception. Cone BCs are particularly critical for central vision, as they process high-acuity, color-discriminating signals in the primate macula, a specialized region responsible for fine detail and reading vision.

Rodent transplantation studies have shown that developing ROs can integrate with the host retina, form synapses with rod BCs, and restore light responses.[3–5] This process involves active host BC dendritic regrowth and recruitment of horizontal cells,[6,7] suggesting that a developmentally immature graft environment may enhance host plasticity. IRDs with primary rod photoreceptor loss are expected to benefit from rod reconstruction, especially before the secondary loss of central cone photoreceptors, and hold potential for additional cone-mediated restoration in advanced degeneration. This motivated our recent first-in-human clinical study, demonstrating the safety and long-term survival of RO grafts in patients with severely advanced retinal degeneration.[8] However, direct evidence that primate cone BCs can regrow dendrites and form functional synapses

Received 22 January 2026; accepted 18 June 2026;
https://doi.org/10.1016/j.ymthe.2026.06.037.

Correspondence: Michiko Mandai, Department of Ophthalmology, Kobe City Eye Hospital, Kobe 650-0047, Japan.
E-mail: e_lab.mandai@kcho.jp

Published by Elsevier Inc. on behalf of The American Society of Gene and Cell Therapy.
This is an open access article under the CC BY license (http://creativecommons.org/licenses/by/4.0/).

with donor photoreceptors for pathologies involving the central cone photoreceptors is lacking. Rodent models are limited by the absence of a macula, and our prior non-human primate (NHP) transplants, which primarily targeted rod reconstruction outside the macula,[9,10] were not positioned to assess cone pathway rewiring in the foveal region. Similarly, our primate macular hole study demonstrated anatomical success, but synaptic contacts between host BCs and graft photoreceptors could not be confirmed due to sample limitations.[11]

In the human retina, dendritic outgrowth of rod BCs has been observed under aging and pathological conditions,[12–14] but cone BC plasticity remains uncertain. A recent NHP study demonstrated that midget BCs in the fovea of zones with complete photoreceptor degeneration failed to extend dendrites toward the neighboring photoreceptors,[15] leaving open the question of whether cone BCs in the primate macula retain the capacity to rewire and restore functional cone-mediated vision.

In the present study, we addressed this gap by transplanting *ISL1*$^{-/-}$ RO sheets derived from human embryonic stem cells lacking ON-BCs into an acute laser-induced macular photoreceptor ablation NHP model. We previously reported that removing graft-derived ON-BCs eliminates competition for synaptic partners and enables direct assessment of host-driven rewiring,[16,17] and that *ISL1*$^{-/-}$ ROs improve synaptic contact and increase retinal sensitivity relative to wild-type ROs,[16,17] with rod-dominant recovery of some physiological retinal functions including background adaptation and temporal resolution in rodents.[18] In the current study, we demonstrate that primate rod and cone BCs extend dendrites into grafts, form canonical synapses with transplanted photoreceptors, and support the recovery of ON- and OFF-cone BC responses as assessed by focal macular electroretinography (FMERG). These findings provide the first demonstration of functional host-graft synaptic integration in the primate macula and highlight the potential of *ISL1*$^{-/-}$ ROs as a strategy to restore cone-mediated central vision in macular degeneration.

RESULTS

Generation of a laser-induced macular photoreceptor ablation NHP model

Photoreceptors were selectively ablated by laser photocoagulation in the parafoveal and extrafoveal regions, including areas with the highest cone-BC density (Figure S1A).[19] Laser power was first optimized by testing coagulation outside the macula at 40–150 mW; 150 mW caused immediate retinal hemorrhage (Figure S1B). Autofluorescence imaging revealed hyperfluorescence immediately after at all power levels, transitioning to hypofluorescence by 1 month (Figure S1B). On optical coherence tomography (OCT), diffuse opacification of the outer nuclear layer (ONL) immediately after laser coagulation at 60–80 mW consistently predicted selective ONL loss at 1 week and 1 month without inner nuclear layer (INL) damage (Figure S1C).

Based on these findings, parafoveal and perifoveal coagulation was performed at 40 and 70 mW (Figure S2A). At 1 month, autofluorescence showed mottling in the coagulated area, indicating localized retinal pigment epithelium (RPE) impairment (Figure S2A). OCT confirmed complete ONL loss with 70 mW, while the ONL partially remained at 40 mW (Figure S2B). FMERG recordings from 70 mW lesions were nearly non-recordable, whereas 40 mW lesions retained a moderate b-wave (Figure S2C). Histologically, 70 mW photocoagulation selectively ablated the ONL, with loss of rhodopsin-, L/M opsin-, S opsin-, and recoverin-positive photoreceptors, while preserving INL integrity (Figures S2D–S2F). Inner retinal cells positive for secretagogin (SCGN) (diffuse bipolar [DB] 1, 6), calretinin (amacrine cells), and calbindin (horizontal cells [HCs], DB3a and DB6), were present at comparable levels across 40 mW, 70 mW, and untreated eyes (Figures S2F and S2G). RPE65-positive cells were decreased in 70 mW lesions, indicating partial RPE damage (Figure S2H), and increased glial fibrillary acidic protein (GFAP) expression confirmed Müller cell activation consistent with retinal degeneration (Figure S2I). Based on these results, 70 ± 10 mW was selected for subsequent experiments.

Dendrites of multiple BC subtypes consistently retracted following photoreceptor loss

To confirm that our laser model recapitulates the synaptic denervation seen in IRDs, we first characterized BC dendrite retraction and loss of postsynaptic markers following photoreceptor ablation. Dendrite length, defined as the distance from the INL border to the longest dendritic tip, was measured across BC subtypes. Calbindin-positive BCs were distinguished from calbindin-positive HCs by the presence of axons extending into the inner plexiform layer (IPL).

Protein kinase Cα (PKCα)-positive ON-BCs (DB4, DB5, and rod BCs), SCGN-positive (DB1 OFF-BC and DB6 ON-BC), and calbindin-positive BCs (DB3a OFF-BC and DB6 ON-BC) showed significant dendrite retraction following ONL ablation (Figures 1A–1I). Consistent with this, postsynaptic marker mGluR6 immunoreactivity appeared partially clotted and redistributed to cell bodies, as previously reported in laser-treated and genetically degenerated retinas (Figures 1J–1K′).[1,20,21] Conversely, GluK1, an OFF-cone BC postsynaptic marker, was retained at the synaptic site, though at reduced levels (Figures 1L–1M′). Together, these findings confirm that near-complete photoreceptor ablation produces complete ON-BC denervation with dendrite retraction, validating our laser models as a platform for studying host-graft synaptic integration.

RO grafts at transplantation contain cone-committed photoreceptor progenitors with no rod photoreceptors

ROs derived from the KhES-1 CRX::Venus human embryonic stem cell (ESC) line (*ISL1*$^{+/+}$) and its genome-edited *ISL1*$^{-/-}$ derivative (330A16)[17] were transplanted at approximately differentiation day 60 (dd60) (Figures S3A and S3B). At this stage, ROs exhibited a characteristic continuous neuroepithelial layer positive for the retinal progenitor markers CHX10/PAX6 and apical/basal expression of CRX::Venus/BRN3, as confirmed by immunohistochemistry (IHC) (Figures S3A′, S3A″, S3B′, and S3B″). Most CRX-positive photoreceptor progenitors were committed to the cone photoreceptor

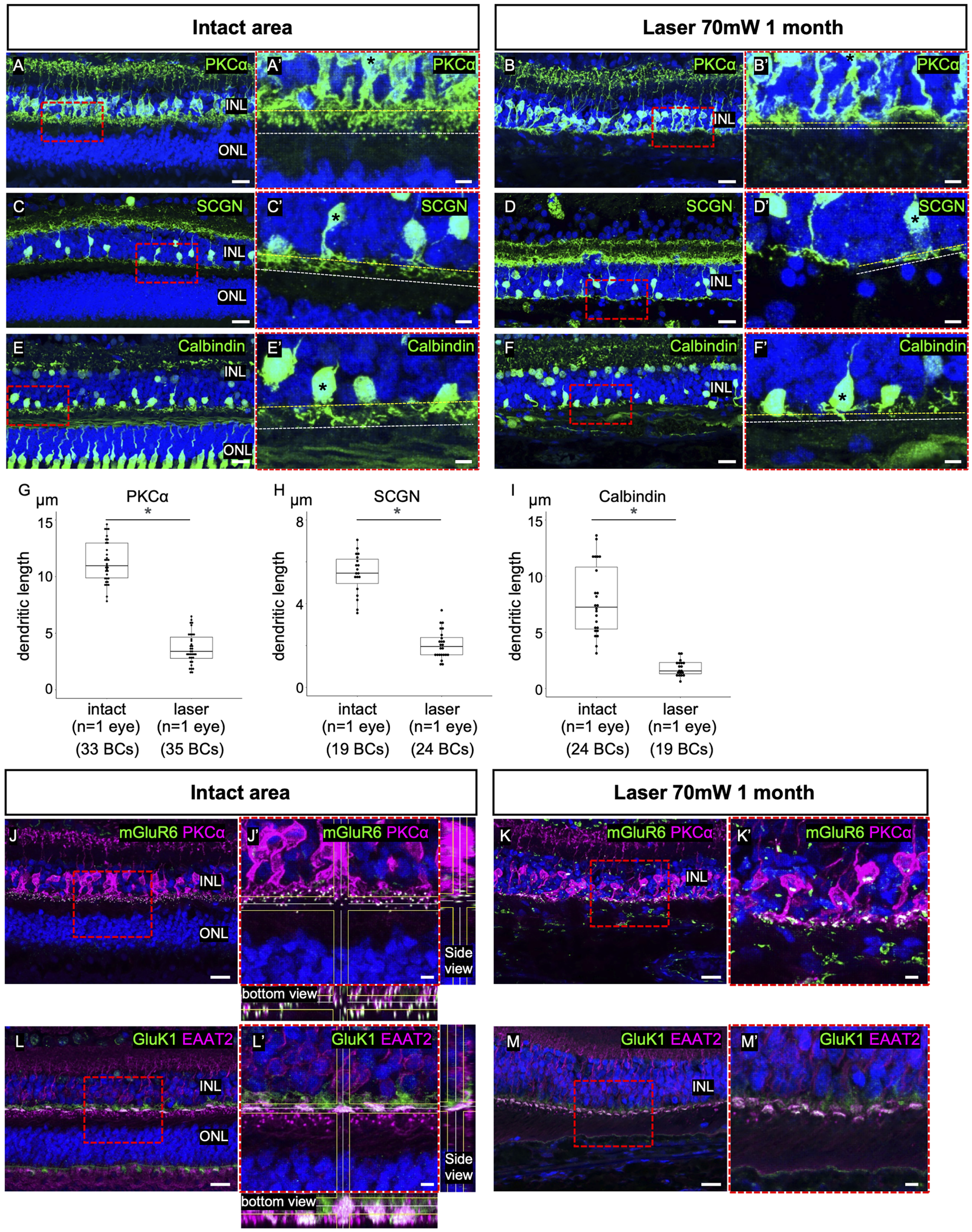

Figure 1. Retraction of BC dendrites following laser treatment

(A–F′) Immunohistological analysis of dendritic changes in three BC subtypes at 1 month after laser treatment relative to those in the intact areas. Representative images show PKCα-positive BCs (A–B′), SCGN-positive BCs (C–D′), and Calbindin-positive BCs (E–F′). The yellow dashed line indicates the bottom of the INL, and the white dashed line indicates the longest dendritic tip of the asterisk-marked cell, used to determine dendrite length. Red dashed rectangles indicate an enlarged region. Scale bars, 20 μm (A, B, C, D, E, and F) and 5 μm (A′, B′, C′, D′, E′, and F′) (G–I) Quantitative analysis of dendritic length for PKCα-positive BCs (G), SCGN-positive BCs (H), and Calbindin-positive BCs (I). Statistical significance was assessed using the Wilcoxon rank-sum test (*$p < 0.05$). (J–K′) Changes in mGluR6-positive postsynaptic markers in intact and laser-treated areas. Scale bars, 20 μm (J and K) and 5 μm (J′ and K′). (L–M′) Changes in GluK1-positive postsynaptic markers in intact and laser-treated areas. Scale bars, 20 μm (L and M) and 5 μm (L′ and M′).

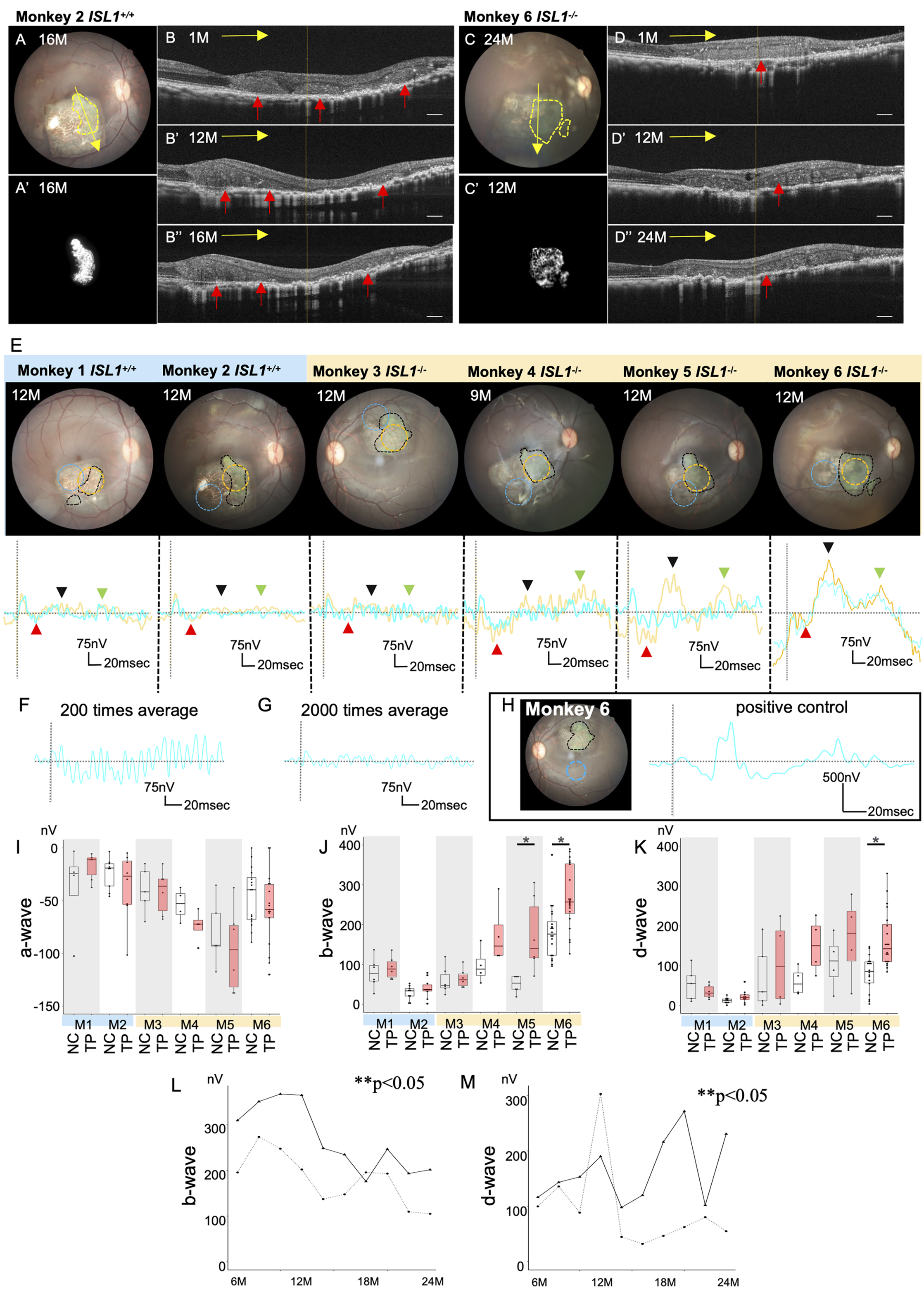

(legend on next page)

lineage, while NRL-positive rod photoreceptors and precursor cells had not yet emerged (Figures S3A‴, S3A⁗, S3B‴, and S3B⁗).

Long-term graft survival achieved through improved immunosuppression in the primate macula

Six RO sheets (~1 × 1.5 mm) were transplanted into the eyes of six monkeys (monkeys 1–2: *ISL1*$^{+/+}$ RO sheets; monkeys 3–6: *ISL1*$^{-/-}$ RO sheets), and graft survival was monitored by CRX::Venus fluorescence and OCT (Figures 2A–2D″ and S4A–S4H″). Four monkeys completed follow-up as planned: monkeys 1, 3, and 5 for 1 year and monkey 6 for 2 years. Monkey 1 exhibited partial graft loss due to suspected rejection but retained the remaining graft for 1 year. In contrast, monkeys 2 and 4 showed significant graft loss at 17 and 9 months, respectively, likely due to rejection and endophthalmitis, and were therefore redirected to earlier histological analyses (Table S1).

Overall, CRX::Venus fluorescence increased until approximately 13 weeks post transplantation and remained stable thereafter (Figure S4I), with graft thickness following a similar trajectory, plateauing at approximately 10 weeks (Figure S4J).

Monkey 1 lost half of the graft between 6 and 8 weeks post transplantation (Figure S5A), with no signs of rejection such as leakage from grafts by fluorescein angiography (FA) (Figure S5B). However, OCT revealed increased choroidal thickness at 6 weeks, suggesting potential immune cell accumulation (Figures S5C). Subtenon triamcinolone acetate (STTA) injections stabilized the graft, enabling 1-year follow-up with no further loss. IHC analysis at 1 year showed no infiltration of immune cells (Figures S5D–S5G). Monkey 2 showed rapid graft loss between 16 and 17 months post transplantation (Figure S6A), with no fluorescein leakage on FA (Figure S6B), but OCT demonstrated graft shrinkage and increased choroidal thickness at 17 months (Figure S6C), suggesting rejection. IHC at 17 months confirmed prominent immune cell accumulation at the graft site (Figures S6D–S6F′).

Graft loss in monkey 1 prompted us to refine our systemic cyclosporine protocol to maintain serum concentrations above 70 ng/mL (Figures S7A and S7B). When oral intake was insufficient, subcutaneous cyclosporine was administered accounting for post-injection concentration decline (Figures S7C and S7D), successfully stabilizing serum levels in monkey 3 from 6 months post transplantation (Figure S7E).

Monkey 4 developed postoperative endophthalmitis at 3 weeks, presenting with anterior chamber fibrin and vitreous opacity. Vitrectomy and intravitreal vancomycin and ceftazidime restored graft visibility; however, CRX::Venus brightness and retinal thickness declined between 7 and 9 months (Figures S4I, S4J, and S8A–S8C). IHC at 9 months showed no significant immune cell accumulation, although focal choroidal excavation was observed at the graft site (Figures S8D–S8G).

ON-BC functional recovery in half of *ISL1*$^{-/-}$ RO-grafted eyes with long-term stability

To evaluate the functional consequences of host-graft synaptic integration, we performed longitudinal FMERG every 2 months beginning at 6 months post transplantation. To minimize inter-individual variability, recordings of 10° circle from the grafted and non-grafted areas within the same ONL-ablated lesion were compared in each eye (Figure 2E). Increasing waveform averaging to 2,000 sweeps improved signal-to-noise ratios, enabling reliable isolation of the a-, b-, and d-wave components, reflecting photoreceptor outer segment (OS) currents, ON-BC, and OFF-BC responses, respectively; recordings from intact retina served as positive controls (Figures 2F–2H).

By pooling recordings across the entire follow-up period, two of four *ISL1*$^{-/-}$ RO transplanted eyes (monkeys 5 and 6) demonstrated significantly higher b-wave amplitudes in grafted areas compared to non-grafted areas, with median improvements of 21.6% and 17.5%, respectively, relative to positive controls, while a-wave amplitudes remained unchanged across all eyes (Figures 2I and 2J). Monkey 6 additionally showed a significant d-wave enhancement, suggesting restoration of OFF-bipolar signaling (Figure 2K). No significant improvements were observed in *ISL1*$^{+/+}$ grafted eyes. Together with our previous rat transplantation study using the same RO lines in which *ISL1*$^{-/-}$ grafts showed enhanced synaptic contact and restoration of light responsiveness,[17] the present results

Figure 2. Long-term functional integration of transplanted retinal organoids in the primate macula

(A–D) Representative fundus photographs, FA images showing CRX::Venus expression, and corresponding OCT images showing the location of transplanted retinal organoid (RO) sheets (yellow outlines) within laser-ablated macular regions in *ISL1*$^{+/+}$ and *ISL1*$^{-/-}$ recipient eyes at multiple post-transplantation time points. Red arrows indicate the graft-host interface. Scale bars, 300 μm (B–D″). (E) Representative focal macular electroretinography (FMERG) waveforms recorded from grafted regions (yellow-dot circles) (TP) and adjacent non-grafted control regions (blue circles) (NC) within the same eye. Arrowheads indicate a-, b-, and d-wave components. (F and G) Comparison of the FMERG waveforms obtained using 200 versus 2,000 averages, demonstrating improved signal-to-noise ratio and reliable isolation of ERG components with increased averaging. (H) Positive control from intact retina on the opposite side of the fovea, confirming waveform fidelity. (I–K) Cross-sectional comparisons of a-wave (I), b-wave (J), and d-wave (K) amplitudes between grafted (TP) and non-grafted (NC) areas obtained from matched sites within the same laser-ablated lesion. Data are presented as median and interquartile range from pooled recordings obtained every 2 months starting at 6 months post transplantation throughout the follow-up period. Monkeys 5 and 6 with *ISL1*$^{-/-}$ grafts demonstrated increased b-wave amplitudes indicating ON-bipolar response, while monkey 6 also showed enhanced d-wave amplitudes, indicating restoration of OFF-bipolar signaling. (* Wilcoxon rank-sum tests, $p < 0.05$) (L) Longitudinal b-wave trajectories in monkey 6 demonstrate sustained enhancement in grafted regions compared to non-grafted controls from 6 to 24 months post transplantation. (M) Longitudinal d-wave trajectories in monkey 6 reveal delayed yet significant increase in OFF-pathway responses, suggesting potential maturation of connectivity from 13 months onward. In (L) and (M), Solid and dotted lines indicate the longitudinal changes in b- and d-wave amplitudes in grafted and non-grafted areas, respectively. Statistical significance was assessed using Wilcoxon signed-rank tests (** $p < 0.05$).

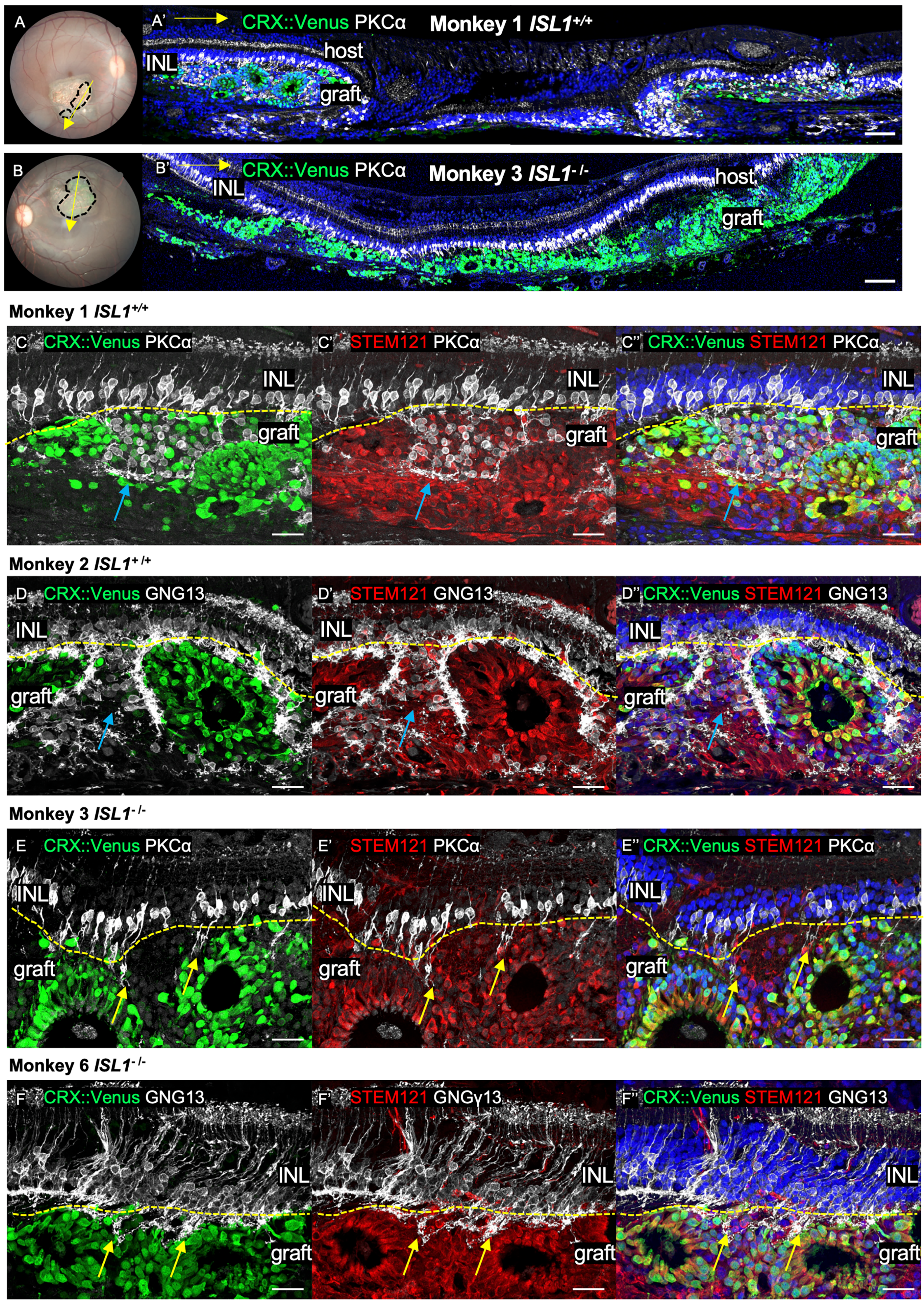

(legend on next page)

consistently suggested that elimination of donor-derived ON-BCs may facilitate host-driven circuit rewiring.

Longitudinal analysis in monkey 6 revealed consistently higher b-wave amplitudes in the grafted area than in non-grafted area from 6 to 24 months, while d-wave enhancement emerged after 1 year (Figures 2L and 2M). Cross-sectional comparisons across successive 6-month periods confirmed significantly higher b-wave amplitudes throughout follow-up, whereas d-wave improvements reached significance only in later periods (13–18 and 19–24 months), suggesting that OFF-pathway reconstruction may require prolonged maturation (Figures S9B and S9C). Collectively, these findings demonstrate stable ON-BC functional recovery in two of four *ISL1*$^{-/-}$ RO-grafted eyes, including one eye followed for up to 2 years in which an additional potential sign of OFF-pathway reconstruction emerged beyond 1 year post transplantation.

Absence of ON-BCs in *ISL1*$^{-/-}$ grafts revealed extensive host BC dendrite regrowth toward graft photoreceptors

The eyes of five monkeys, Monkeys 1 (*ISL1*$^{+/+}$), 2 (*ISL1*$^{+/+}$), 3 (*ISL1*$^{-/-}$), 4 (*ISL1*$^{-/-}$), and 6 (*ISL1*$^{-/-}$), were processed for IHC analysis after euthanasia at 61, 72, 59, 40, and 109 weeks post transplantation, respectively. CRX::Venus-positive grafted photoreceptors were distributed throughout the grafted area in all animals (Figures 3A–3B′ and S10A–S10C′). As expected, PKCα and GNGγ13 (a pan-ON-BC marker) were present in *ISL1*$^{+/+}$ grafts but absent in *ISL1*$^{-/-}$ grafts (Figures 3C–3F″). This enabled clear visualization of PKCα-positive and GNGγ13-positive BC dendrites extending into *ISL1*$^{-/-}$ grafts to contact GFP-positive graft photoreceptor axon terminals (Figures 3E–3F″, yellow arrows). The proportion of photoreceptors was significantly higher in *ISL1*$^{-/-}$ grafts than in *ISL1*$^{+/+}$ grafts across animals (Figures S10D and S10E). In both genotypes, grafts consistently developed mature cone photoreceptors with appropriately localized L/M opsin expression in the OS, whereas unpolarized expression of rhodopsin indicated immature or stressed rod photoreceptors (Figures S11A–S11E′).

Müller glia may act supportively rather than block host-graft integration

In the host retina, Müller cells showed moderate GFAP expression at 1 year in the *ISL1*$^{+/+}$ eye (monkey 1) and at 2 years with *ISL1*$^{-/-}$ eye (monkey 6)—apparently less than that observed 1 month after the laser treatment—and GFAP-positive barrier scarring did not form between the host and graft in either eye. Within RO grafts, Müller cells were consistently present alongside graft photoreceptors, as identified by glutamine synthetase (GS) expression, suggesting structural support for photoreceptor alignment within photoreceptor rosettes (Figure S12). GFAP expression in graft Müller cells appeared elevated compared to that in host Müller cells, potentially reflecting environmental stress distinct from the cells' native developmental setting. Interestingly, consistent with our previous reports,[10,17] despite the presence of GS-positive Müller fibers, the host side of rosettes, where photoreceptors are putatively integrating with the host, showed less GFAP upregulation compared to the RPE-proximal side, where photoreceptors are not integrating (Figure S12). This raises the possibility that integrating graft photoreceptors may locally suppress activation of neighboring Müller cells.

Macular environment may favor cone survival over rod development in grafts

Given the immature or stressed rhodopsin expression patterns in rod photoreceptors of the current macular grafts, which contrasted with our previous extramacular grafts in macaque eyes,[9,10] we quantified the cone proportion (Figures 4A, 4B, and S13). Cone photoreceptors were primarily located apically, whereas rod photoreceptors were positioned basally, mirroring the layered structure of the human retina (Figure 4A). Unexpectedly, the cone proportion in the current macular grafts was markedly higher (75%–92%; Figures 4B and S13) than that observed in our previous mid-peripheral grafts (10%–15%).[9] A correlation was also observed between the photoreceptor rosette size and cone proportion: smaller rosettes had a higher cone proportion (Figure 4C). This suggests that rod photoreceptors, which had not yet emerged at the time of transplantation, may have failed to develop properly or may have been selectively lost due either to the influence of the macular microenvironment or differential vulnerability of rods to the stress of macular transplantation, including inflammation.

ISL1$^{-/-}$ RO transplantation significantly improved contact efficiency between host BCs and graft photoreceptors

To confirm the primary rationale for using *ISL1*$^{-/-}$ RO grafts, we evaluated the extent of direct contact between host BCs and grafted photoreceptor rosettes, categorizing the contact efficiency into three grades: good (direct contact), fair (partial contact), and poor (no contact) (Figure 4D). Photoreceptors in *ISL1*$^{-/-}$ grafts (monkeys 3 and 6) showed a trend toward a higher direct contact ratio with host BCs than those in *ISL1*$^{+/+}$ grafts (monkeys 1 and 2) (Figure 4E). Since findings from our previous work suggested that host retinal disruption may reduce the transplantation efficacy,[17] we further investigated whether graft cell migration disrupted the host IPL. Calretinin immunostaining of the host retina showed an intact IPL structure above the grafts in four monkey eyes examined (four sections each from monkeys 1, 3, 4, and 6) (Figure S11F).

Figure 3. *ISL1*$^{-/-}$ grafts enable unambiguous visualization of host BC dendrite regrowth

(A–B′) Representative immunostaining of monkey retinas transplanted with *ISL1*$^{+/+}$ (A) or *ISL1*$^{-/-}$ (B) RO sheets at 12 months post transplantation. Graft photoreceptors were identified within CRX::Venus-positive rosettes in the graft area, and host bipolar cells were defined by their anatomical location within the host INL. In *ISL1*$^{-/-}$ grafts lacking graft-derived PKCα-positive ON-bipolar cells, all PKCα-positive dendrites extending from the host were therefore of host origin. Yellow arrows in the fundus images indicate the orientations of the retinal sections. Scale bars, 100 μm. (C–F″) Immunostaining with PKCα and GNGγ13 antibodies demonstrates the presence of ON-BCs between the graft photoreceptor rosettes in *ISL1*$^{+/+}$ grafts (C–D″, blue arrows), while their absence in *ISL1*$^{-/-}$ grafts enabled unambiguous visualization of host BC dendrites extending into the grafted area (E–F″, yellow arrows). Scale bars, 30 μm (C–F″).

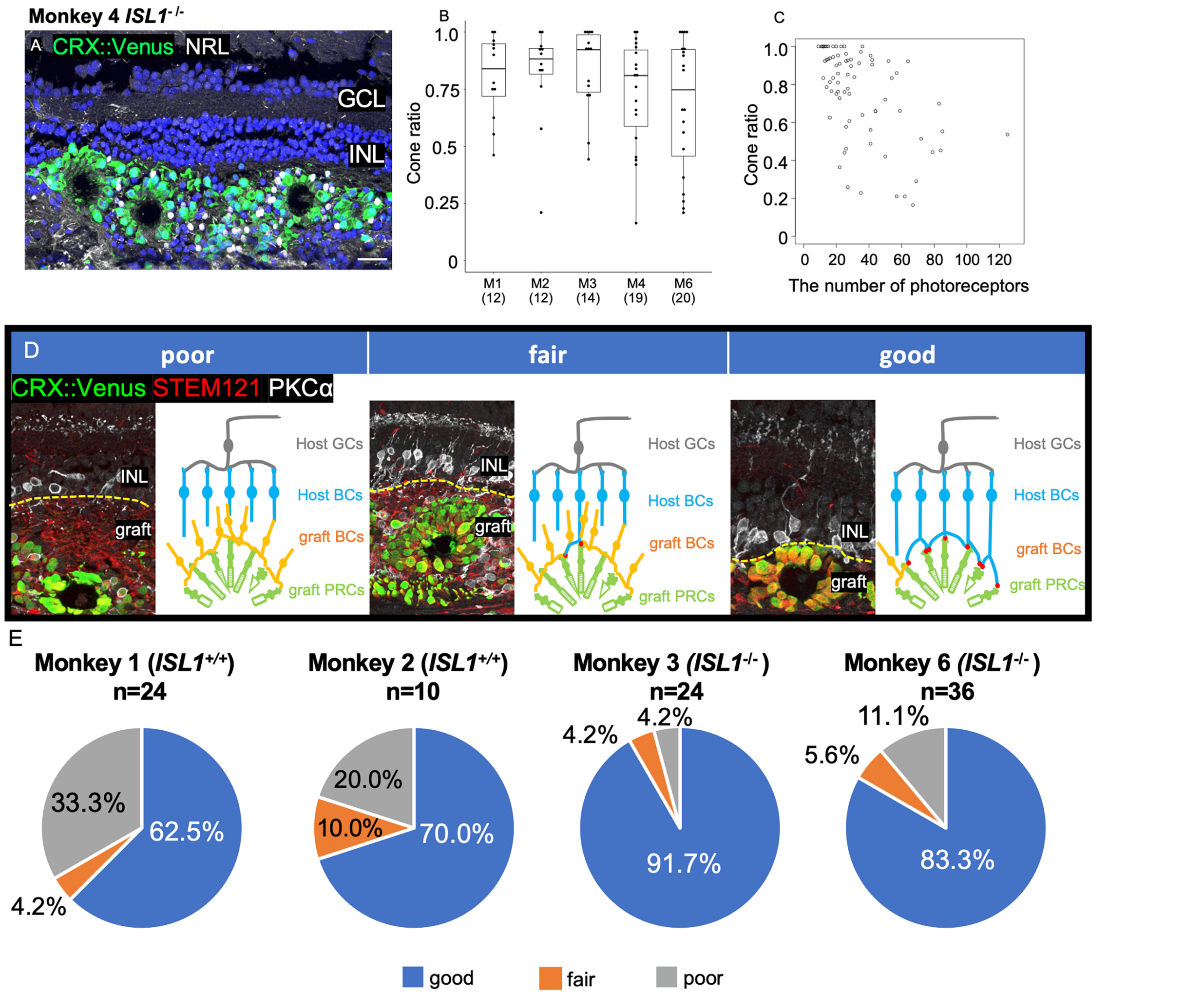

Figure 4. High cone proportion in macular grafts and improved host-graft contact with *ISL1*$^{-/-}$ grafts

(A) Representative image of CRX::Venus-positive photoreceptor rosettes in the graft area and NRL immunostaining to distinguish between cone and rod photoreceptors in the graft (monkey 4). Scale bars, 30 μm. (B) Quantification of cone photoreceptor ratio per rosette across individual monkeys (M1, 12 rosettes; M2, 12 rosettes; M3, 14 rosettes; M4, 19 rosettes; M6, 20 rosettes). (C) Correlation analysis of the cone photoreceptor ratio and total photoreceptor count per rosette ($n = 77$ rosettes, Spearman's rank correlation coefficient = −0.59), suggesting an inverse relationship between rosette size and cone photoreceptor proportion. (D) Schematic representation of host BC and graft photoreceptor contact patterns. Contact was graded into three categories: poor (no contact), fair (partial contact), and good (direct contact). (E) Quantification of host BC-graft photoreceptor contact across monkeys. (monkey 1 [$n = 24$ rosettes]: good 62.5%, fair 4.2%, and poor 33.3%; monkey 2 [$n = 10$ rosettes]: good 70.0%, fair 10.0%, and poor 20.0%; monkey 3 [$n = 24$ rosettes]: good 91.7%, fair 4.2%, and poor 4.2%; monkey 6 [$n = 36$ rosettes]: good 83.3%, fair 5.6%, and poor 11.1%).

Multiple BC subtypes regrow dendrites in response to *ISL1*$^{-/-}$ RO engraftment

To evaluate host BC plasticity in response to *ISL1*$^{-/-}$ RO transplantation, we assessed the dendritic extension across three BC subtypes in grafted and non-grafted laser ablation areas. BC cytoplasmic and dendritic structures appeared disorganized after 9 months (monkey 4) to 1 year (monkey 3) in non-grafted areas (Figures 5A–5C′), where the dendrite length remained unchanged from 1 month through 9 months to 1 year (Figures 5E–5G), confirming that dendrite regrowth does not occur spontaneously after photoreceptor loss. In contrast, all three BC subtypes in the grafted areas showed significant dendrite elongation at both 9 months to 1 year and 2 years (monkey 6) relative to non-grafted controls, with dendrites projecting toward grafted photoreceptor axons (Figures 5E–5G). Overall, dendrite length did not differ significantly between these two time points, suggesting that graft-induced dendrite regrowth occurs primarily in the early post-transplantation period and that the resulting elongation may subsequently be stabilized, potentially through synapse formation.

Host-graft contacts reconstitute canonical ON-, OFF-, and cone-specific synaptic pathways

To determine whether the observed host-graft contacts represented true circuit reconstruction rather than non-specific apposition, we

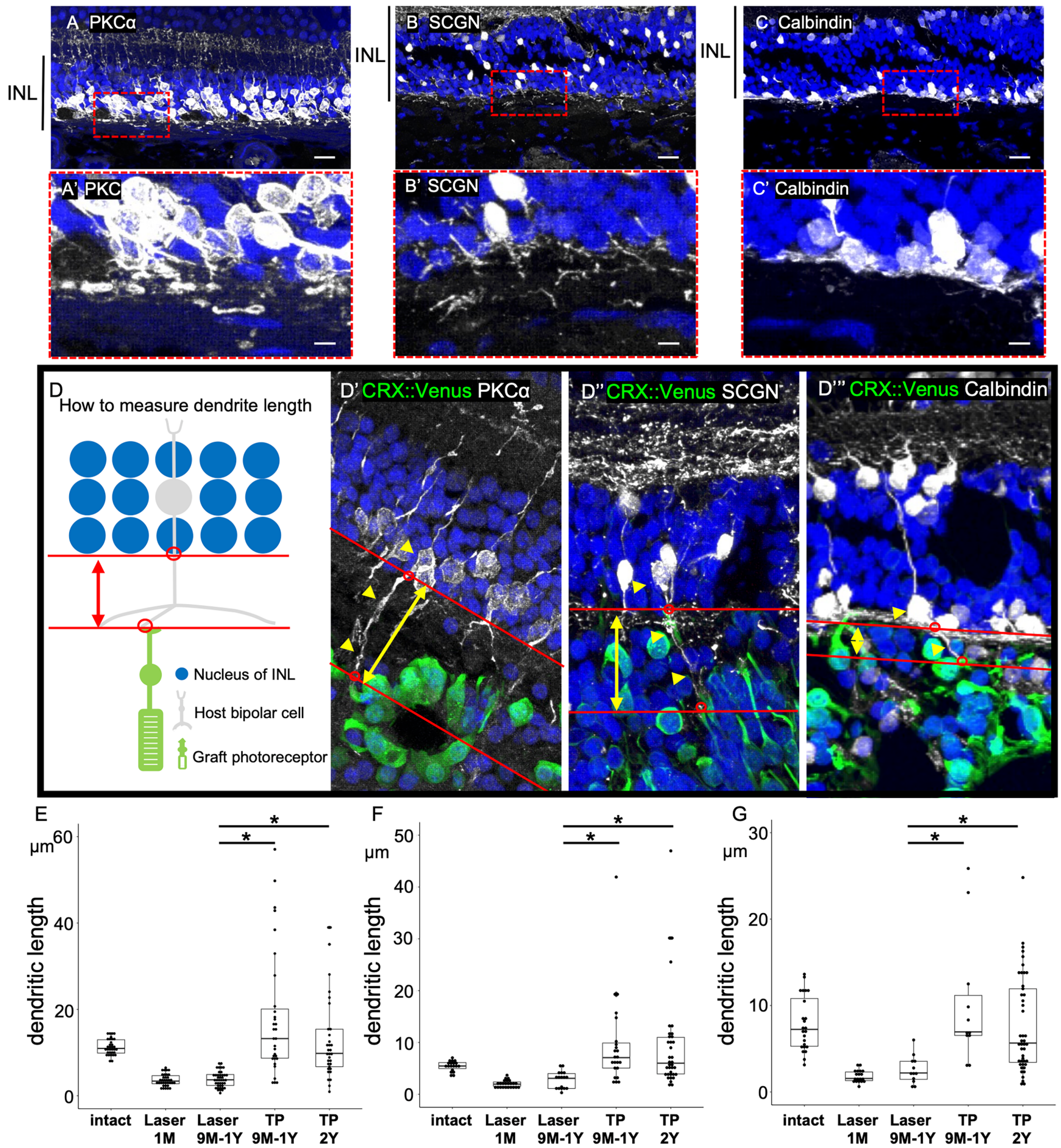

Figure 5. Dendritic regrowth across multiple host BC subtypes in eyes with *ISL1*$^{-/-}$ grafts

(A–C′) Immunohistochemical analysis of host BC dendrites 1 year post laser treatment (monkey 3). Representative images show dendrites of PKCα-positive BCs (A and A′), SCGN-positive BCs (B and B′), and Calbindin-positive BCs (C and C′). Scale bars, 5 μm. (D) Schematic drawing of dendrite length measurement in host BCs. (D′–D‴) Representative histological images depicting dendrite extension in the three BC subtypes (yellow arrowheads). The dendrite length was measured from the bottom of the INL to the longest axon tip of the graft photoreceptors (yellow double-headed arrows). (E–G) Changes in dendrite length of host BCs before and after laser treatment and post transplantation. (E) PKCα-positive BCs (intact: n = 1 eye, 33 BCs; laser at 1 month [1M]: n = 1 eye, 35 BCs; laser at 9 months to 1 year [9M–1Y]: n = 2 eyes, 41 BCs; transplantation 9M–1Y: n = 1 eye, 31 BCs; transplantation at 2 years [2Y]: n = 1 eye, 33 BCs). (F) SCGN-positive BCs (intact: n = 1 eye, 19 BCs; laser 1M: n = 1 eye, 24 BCs; Laser 9M–1Y: n = 2 eyes, 17 BCs; transplantation 9M–1Y: n = 2 eyes, 23 BCs; transplantation 2Y: n = 1 eye, 36 BCs). (G) Calbindin-positive BCs (intact: n = 1 eye, 24 BCs; laser 1M: n = 1 eye, 19 BCs; laser 9M–1Y: n = 2 eyes, 12 BCs; transplantation 9M–1Y: n = 1 eye, 11 BCs; transplantation 2Y: n = 1 eye, 45 BCs). Statistical significance was assessed using the Wilcoxon rank-sum test (*$p < 0.001$).

characterized the synaptic interface using pathway-specific molecular markers. Since *ISL1*$^{-/-}$ ROs lack donor-derived ON-BCs, host-driven synaptic integration was unambiguously identified. ON-BCs were identified by PKCα (rod-BC and DB4 and DB5) and OFF-BCs by EAAT2, while calbindin and SCGN labeled both ON-BC (DB6) and OFF-BC (DB3a and DB1, respectively) subtypes (Table S2).

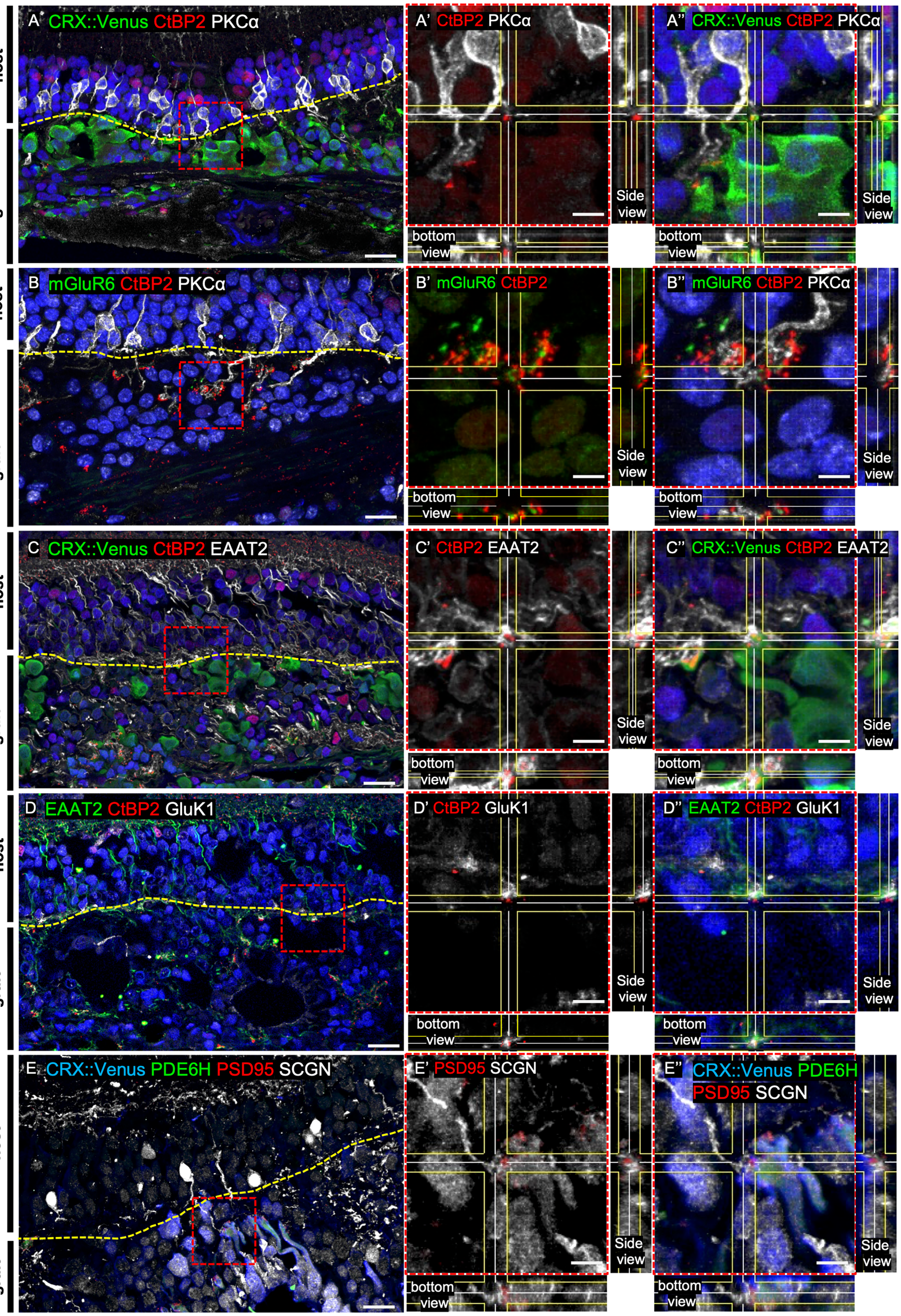

(legend on next page)

For the ON-pathway, PKCα-positive BC dendrites formed direct contacts with CtBP2-positive presynaptic ribbons at the axon terminals of graft photoreceptors, with precise apposition of the postsynaptic ON-BC marker mGluR6, confirming re-establishment of canonical invaginating synapses (Figures 6A–6B″). For the OFF-pathway, GluK1-positive dendritic tips of host EAAT2-positive OFF-BCs contacted graft photoreceptor axon terminals (Figures 6C–6D″). For cone-specific circuitry, calbindin- and SCGN-positive cone BCs also established synaptic contact with CRX::GFP-positive photoreceptor axon terminals including cone pedicles (Figures S14A–S14B″), with HC processes also recruited potentially contributing to classic photoreceptor triad synapses (Figures S14C–S14C″). Cone-specific BC-photoreceptor contact was further confirmed between SCGN-positive BC and PDE6H/GFP-double-positive photoreceptors in the graft (Figures 6E–6E″). Additionally, quite a few PKCα-BCs were also observed contacting cone pedicles of graft photoreceptors (Figures S14D–S14D″); as PKCα labels both rod BCs and DB4/5 cone ON-BCs in the primate retina, the identity of these cells remains to be determined. Collectively, these findings suggest that host-graft integration in the primate macula involves precise, pathway-specific, and cell-type–appropriate synaptic reconstruction, although the identity of PKCα-positive cells contacting cone pedicles warrants further investigation.

Ultrastructural imaging demonstrated host-graft triad formation in cone pedicles

High-resolution ultrastructural imaging using electron microscopy (EM) and serial block-face scanning EM (SBFSEM) was conducted at 55 weeks post transplantation (monkey 5). Graft photoreceptor axons, originating from cell bodies confirmed within the CRX::Venus-positive rosettes within the graft, were traced to their terminal in the host-graft borders (Figures 7A–7D, S15A, and S15B), where synaptic ribbons formed multiple triad synapses with invaginating BCs and likely HCs in the observed pedicles, consistent with the IHC observation (Figures 7E, S14C–S14C″, and S15C); dyad configurations potentially demonstrating the preceding likely HC invagination without BC participation were also noted (Figure 7F), consistent with previous observation from mouse *Isl1*$^{-/-}$ RO transplantation in an *rd1* mouse model.[7] The coordinated recruitment of both BCs and HCs indicates that the graft environment supports comprehensive reconstruction of macular synaptic architecture rather than isolated pairwise contacts. SBFSEM further demonstrated synaptic connections between graft photoreceptor ribbons and both host ON-BCs and OFF-BCs (Figures 7G–7I and Video S1). ON-BC dendrites formed canonical invaginating synapses, whereas OFF-BC dendrites made basal contact with transplanted cone pedicles (Figures 7J and 7K).

DISCUSSION

By employing *ISL1*$^{-/-}$ ROs, in which genome editing was applied to improve graft efficacy by eliminating donor-derived ON-BCs, we demonstrate for the first time that both ON- and OFF-cone BCs in the primate macula can actively regenerate dendrites upon photoreceptor engraftment and form functional synapses, with stable integration documented over 2 years in one eye. Additionally, the proportion of cone photoreceptors (∼80%) in the current macular grafts was unexpectedly higher than in our previous extramacular grafts,[9] suggesting that RO grafts may adapt their photoreceptor composition to the implantation site, a potentially advantageous property for tailoring grafts to target either cone- or rod-dominant degeneration.

Functional resilience, pathway-specific maturation, and translational refinement

A major challenge in interpreting transplantation success in the primate macula is the modest absolute amplitude of functional recovery. *In vivo* electrophysiological recordings tend to underestimate graft efficacy relative to subjective examinations,[10] and the robust recovery of host RGC light responses consistently observed in rodent retinas using *ex vivo* multielectrode arrays is not readily detectable by *in vivo* electrophysiological recordings.[16–18] Nevertheless, b-wave increases of 17.5%–21.6% in the current study were remarkably stable across longitudinal assessments, supporting their physiological relevance.

This stability was further contextualized by pathway-specific findings. A significant increase in ON-BC responses in two *ISL1*$^{-/-}$ grafted eyes, compared with adjacent non-grafted controls, suggests that eliminating donor-derived BC competition may facilitate productive host-driven integration. Photoreceptor a-wave amplitudes remained unchanged across all subjects, likely reflecting the rosette-like organization and imperfect orientation of OSs of variable maturity in transplanted tissue. Nonetheless, downstream BC activation occurred despite these structural limitations, demonstrating that host circuits can extract functional signals from grafted photoreceptors even in the absence of robust global photoreceptor currents.

Longitudinal analysis in monkey 6 confirmed that b-wave enhancement was sustained over 24 months, establishing that host-graft rewiring is durable rather than transient. Although FMERG recordings required 2,000 averages to overcome low signal-to-noise ratios, the

Figure 6. Pathway-specific host-graft synaptic reconstruction in eyes with *ISL1*$^{-/-}$ grafts

(A and C) The presynaptic marker, CtBP2, was localized in the axon terminals of GFP-labeled graft photoreceptors, contacting dendritic tips of PKCα-positive bipolar cells (BCs) (A) and EAAT2-positive BCs (C). Scale bars, 15 μm (A and C) and 4 μm (A′, A″, C′, and C″). (B and D) Apposition of CtBP2 (presynaptic) and mGluR6 (postsynaptic) synaptic markers at PKCα-positive BC dendrite tips (B); CtBP2 (presynaptic) and GluK1 (postsynaptic) are juxtaposed at EAAT2-positive BC dendrite tips (D). Scale bars, 15 μm (B and D) and 4 μm (B′, B″, D′, and D″). (E) The presynaptic marker PSD95 localized on graft cone axon terminals labeled with GFP and PDE6H contacted dendritic tips of SCGN-positive BCs. Scale bars, 15 μm (E) and 4 μm (E′ and E″). Graft photoreceptors were identified based on CRX::Venus-positive rosettes in the graft area. Host bipolar cells were defined by their anatomical location within the host INL.

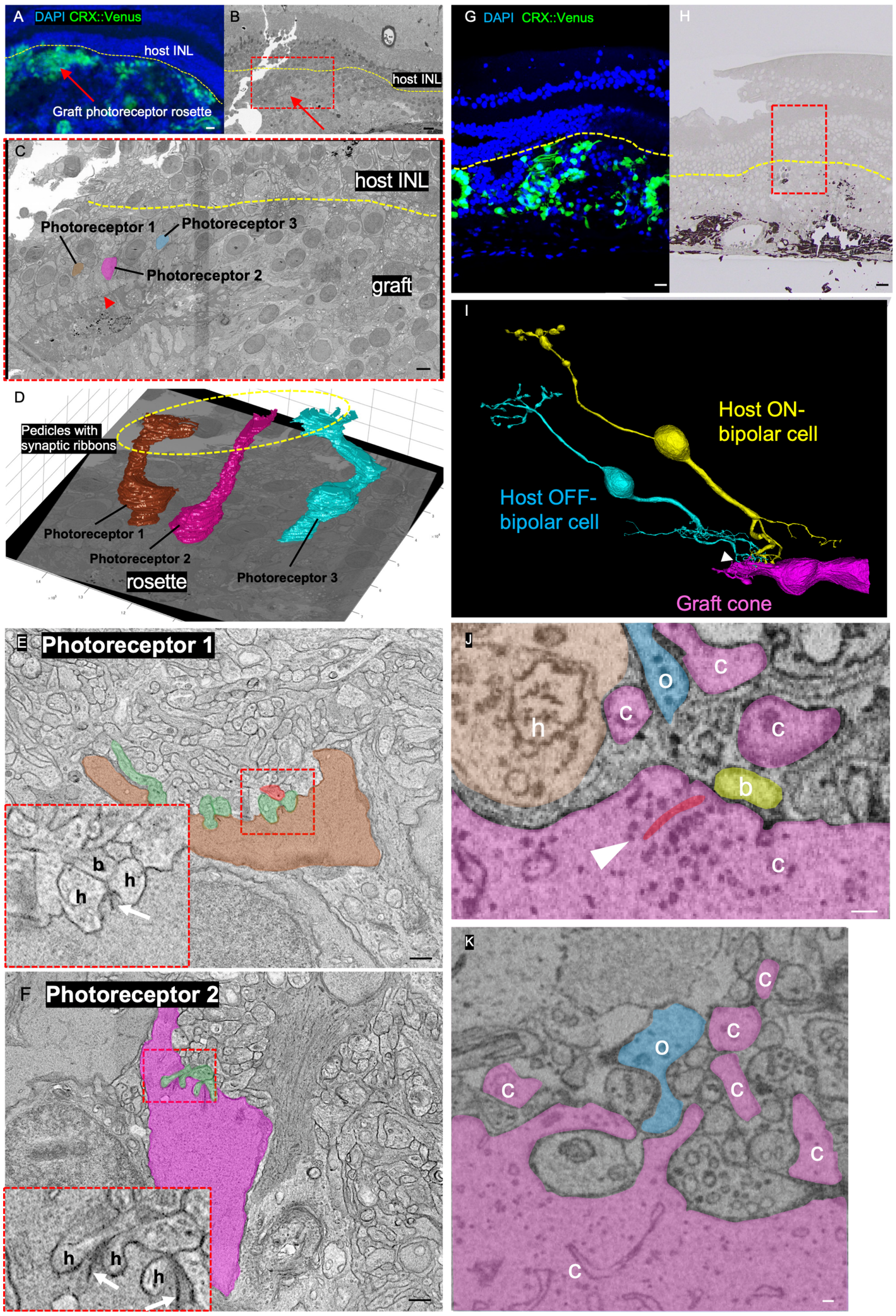

Figure 7. Ultrastructural evidence of triad synapse formation between host BCs and grafted photoreceptors with *ISL1*$^{-/-}$ ROs

(A) A pre-embedding image showing CRX::Venus-positive graft photoreceptors within a rosette (red arrow) in the *ISL1*$^{-/-}$ graft lacking ON-BCs. Scale bar, 20 μm. (B) EM image of the same area from the same original section as in (A), with the red arrow indicating the same rosette. Scale bar, 20 μm. (C) Enlarged view of the region within the red dotted rectangle in (B). Red arrowhead indicates the external limiting membrane of a rosette. Scale bar, 5 μm. (D) 3D reconstruction showing photoreceptors in the graft rosette projecting axons into the host INL. (E and F) Three photoreceptors were traced to their individual axon terminals. The red dotted rectangles highlight two synapse configurations: a characteristic triad synapse (E; photoreceptor 3 shown in Figure S15), where both bipolar cells (b) and likely horizontal cells (h) invaginate the ribbon synapse (white arrow), and a dyad synapse (F), where only likely horizontal cells (h) invaginate without bipolar cell participation. Scale bars, 500 nm. (G) A pre-embedding image for SBFSEM analysis showing CRX::Venus-positive graft photoreceptors within a graft area. Scale bars, 20 μm. (H) EM image of the corresponding region in (G). Scale bars, 20 μm. (I) 3D reconstruction from SBFSEM analysis, revealing direct contact (white arrowhead) between a host ON-bipolar cell and a host OFF-bipolar cell, both located within the host INL, and a grafted cone photoreceptor. (J) Higher-magnification image highlighting the contact site between the grafted cone photoreceptor (pink) and the host ON-bipolar cell (yellow). The synaptic ribbon (white arrowhead, red color) is positioned opposite the host ON-bipolar cell process, confirming synaptic integration. Scale bars, 50 nm. (K) Higher-magnification image showing flat contact between the grafted cone photoreceptor (pink) and the host OFF-bipolar cell (blue). b, bipolar cell; c, cone photoreceptor; h, horizontal cell; o, OFF-bipolar cell. Scale bars, 50 nm.

consistency of responses across multiple time points within the same eye supports their interpretation as genuine physiological signals. The delayed emergence of significant d-wave improvements, observed only after 1 year in one eye, suggests that ON- and OFF-pathway reconstruction may follow distinct temporal trajectories, potentially reflecting differences in synaptic architecture, receptor composition, or activity-dependent refinement in the adult retina.

Mechanistically, *ISL1*$^{-/-}$ ROs lacking donor-derived ON-BCs enabled unambiguous identification of host-driven rewiring. Host PKCα-positive ON-BCs formed canonical invaginating synapses with graft photoreceptors, confirmed by mGluR6 apposition at CtBP2-positive ribbons, while OFF-cone BCs established GluK1-positive synapses characteristic of native OFF-pathway signaling. Ultrastructural analyses further revealed complete triad synapses within graft-derived cone pedicles, with coordinated recruitment of horizontal cells and host ON-BCs. Although foveal midget BCs were once thought to have limited rewiring capacity,[15] a contact with potential midget ON- and OFF-BCs was observed at one graft-derived cone. Multiple host SCGN-positive cone BCs also formed synapses with graft-derived cone photoreceptors, indicating that the adult primate macula retains molecular cues for cell-type-specific synaptic matching even after photoreceptor loss. Together, these findings may support b-wave amplitude as a biomarker of functional integration for future translational studies.

High cone proportion in the current macular grafts

Interestingly, the proportion of cone photoreceptors in both *ISL1*$^{+/+}$ and *ISL1*$^{-/-}$ RO grafts was approximately 80%, markedly higher than the 10%–15% cone ratio observed in our previous extramacular grafts.[9] The key differences between these studies were the transplantation site and the degree of inflammation.

One possibility is a reported high expression of CYP26A1, a retinoic acid (RA)-degrading enzyme, in the adult primate foveal region,[22] resulting in a relatively low local RA environment. Since RA has been reported to promote rod and reduce cone photoreceptor numbers in human ROs *in vitro*,[23] regional differences in RA metabolism may have influenced graft photoreceptor composition. Light exposure in the fovea may additionally affect the differentiation or survival of rod photoreceptors, given their greater sensitivity to light relative to cones.[24] However, given that the normal parafoveal cone proportion is reportedly less than 50%,[25] this mechanism alone may not fully account for the ~80% cone ratio observed here.

Another possibility is a role of inflammation. Inflammatory responses such as macular edema were more frequent in the current study than in the previous extramacular studies,[9] potentially impairing the maturation or survival of late-born rod photoreceptors. Consistent with this, larger rosettes tended to contain a higher proportion of rod photoreceptors, suggesting that rod development was limited or rod photoreceptors were selectively lost in the macular environment. Given that the underlying mechanism remains speculative, these findings underscore the importance of investigating whether the implantation site consistently influences cone-rod composition in transplanted ROs.

Clinical and surgical refinement for translational success

Although our study demonstrates durable, pathway-specific functional integration, several clinical and surgical factors require further optimization.

Immunological management remains a challenge. Most grafts survived long term under systemic and local immunosuppression, but persistent macular edema and graft loss suggested localized immune activation near the fovea, consistent with our previous NHP macular hole study.[11] This contrasts with minimal immune reactions in our previous extramacular grafts,[9,10] suggesting that the macula may be more immunologically vulnerable. However, our recent clinical trial showed no rejection with allogeneic RO graft near the fovea,[8] and prior fetal retinal transplantation studies reported low rejection rates.[26,27] ESC/induced pluripotent stem cell-derived ROs also exhibit low immunogenicity,[28,29] although surgical trauma near the insertion site may trigger localized responses.[30] Reducing retinal incision size and increasing the distance between graft placement and the entry site may help mitigate these responses.

The rosette-like organization of transplanted ROs remains a major barrier to restoring photoreceptor a-wave responses, as it disrupts the OS orientation required for efficient phototransduction. Future strategies, including bioengineered scaffolds, earlier-stage RO transplantation, or extracellular matrix modulation, may improve OS orientation and photoreceptor current generation, increasing the overall functional "take" rate.

Limitations

This study has several limitations. First, we used an acute laser ablation model, which differs from the progressive course of IRDs.[31] This paradigm was deliberately chosen to isolate the BC synaptic capacity under conditions where inner retinal architecture is preserved but native photoreceptor terminals are absent, ensuring that any observed BC synaptic contacts most likely reflect host-graft rather than host-host connectivity. We acknowledge that IRD involves prolonged remodeling, gliosis, and ectopic circuitry. These include rewiring of cone and rod BCs, as documented in classic and contemporary studies[32]; however, transplanted ROs achieved stable engraftment in our previous clinical trial, forming an outer plexiform layer-like structure between the host INL and graft tissues without scarring barriers, as examined by OCT and adaptive optics OCT.[8,33] In these patients, the graft was placed over areas with relatively preserved RPEs, suggesting that the transplantation site and disease stage are critical determinants of stable engraftment. Future studies using chronic genetic NHP models are needed to confirm durability of rewiring during long-standing degeneration.

Second, although the current study involved some variability in the interval between laser treatment and transplantation, OCT confirmed that host photoreceptors were absent by 1 week after laser photocoagulation (Figure S1), suggesting that this variability did not substantially affect the outcomes.

Third, the number of transplanted animals was relatively small; however, this is an inherent limitation of large animal transplantation studies, and the findings in NHPs nonetheless provide meaningful insights relevant to clinical translation.

Finally, although the *ISL1*$^{-/-}$ human ESC line used in this study showed the expected differentiation phenotype and no overt abnormal proliferation after transplantation, formal karyotyping or genome-wide integrity analysis was not performed for the research-grade clone. For future clinical translation, comprehensive assessment of genomic stability will be required using clinical-grade cell lines.

Conclusion

This study provides proof of concept that the primate macula with acute photoreceptor degeneration retains the functional plasticity necessary to rewire with human stem-cell-derived ROs. By demonstrating that host cone and rod BCs can regenerate dendrites and form canonical, stable, and potentially functional synapses with transplanted photoreceptors, we address a long-standing barrier in regenerative ophthalmology. Sustained b-wave enhancement over 1–2 years observed in a subset of animals suggests durable host-graft integration, and delayed d-wave improvement suggests that

OFF-pathway reconstruction may also be possible. Moreover, the use of *ISL1*$^{-/-}$ ROs may improve therapeutic efficacy by reducing synaptic competition. These findings position *ISL1*$^{-/-}$ RO transplantation as a promising approach for restoring central vision in macular degeneration.

MATERIALS AND METHODS

Animals

All animal experiments were conducted in accordance with the local guidelines and the ARVO Statement on the Use of Animals in Ophthalmic and Vision Research. All experimental protocols were approved by the Institutional Animal Care and Use Committee of RIKEN Kobe Branch. Seven cynomolgus monkeys (provided by Eve Bioscience, Hashimoto, Japan) were used in the experiment (one for laser treatment and monkeys 1–6 for transplantation; Table S1). Six RO sheets were used for transplantation in each monkey, and *ISL1*$^{+/+}$ RO sheets were used for transplantation in monkeys 1 and 2. *ISL1*$^{-/-}$ RO sheets were used for the transplantation in monkeys 3–6.

Human ESC line and genome editing

The human ESC (KhES-1) cell line was established and distributed by Kyoto University (NIH approval number: NIHhESC-22-0487). The KhES-1 CRX::Venus line (RIKEN BioResource Center, Cell Number: HES0653)[34] was used to generate the genome-edited *ISL1*$^{-/-}$ line by CRISPR-Cas9-mediated genome editing, as described in detail previously.[17] In brief, a CRISPR-Cas9 construct targeting the *ISL1* locus was introduced by transfection, followed by puromycin selection of transfected colonies. Individual clones were screened by PCR, and two independent lines with bi-allelic *ISL1* disruption (no. 330a16 and no. 330A19) were established. Gene disruption was confirmed by Sanger sequencing, and loss of ISL1 protein was verified by IHC. Both clones showed a consistent ON-bipolar cell-deficient phenotype; the no. 330A16 line, whose functional validation including improved host-graft contact and restoration of light responsiveness after transplantation into nude rats with an advanced retinal degeneration model has been reported previously,[17] was used in the current study. The KhES-1 CRX::Venus and *ISL1*$^{-/-}$ lines were used in this study in accordance with the human ESC research guidelines of the Japanese government.

Cell culture and differentiation of human ESCs

Retinal differentiation was performed using a modified SFEBq method.[35] Subconfluent human ESCs were cultured with 5 μM SB431542 (Sigma-Aldrich, St. Louis, MO, USA; catalog [cat.] #S4317) and 300 nM SAG (Enzo Biochem, Farmingdale, NY, USA; cat. #ALX-270-426-M001) from 24 h before differentiation. Cells were dissociated into single cells using TrypLE Select Enzyme (Thermo Fisher Scientific, Waltham, MA, USA; cat. #A12859-01) suspended in 100 μL of growth-factor-free chemically defined medium (gfCDM), Ham's F-12 Nutrient Mix (Life Technologies, Waltham, MA, USA; cat. #11765-054), IMDM+ GlutaMAX (Life Technologies; cat. #31980-030), CD lipid concentrate (Life Technologies; cat. #11905-031), Knockout Serum Replacement (Life Technologies; cat. #10828-028), and 1-thioglycerol (Sigma-Aldrich; cat. #M6145) with 10 μM Y-27632 (FUJIFILM Wako Pure Chemical, Osaka, Japan; cat. #253-00513); and seeded in low-cell-adhesion 96-well V-bottomed plates (Sumitomo Bakelite, Tokyo, Japan; cat. #MS-9096V) at 1.2 × 10^4/well. On dd3, the aggregates were treated with 1.5 nM recombinant human BMP4 protein (R&D Systems, Minneapolis, MN, USA; cat. #HZ-1045). The gfCDM medium was changed every 3 days from dd6 to dd15. Aggregates on dd18 were transferred to DMEM/F12 + GlutaMAX (Invitrogen, Waltham, MA, USA; cat. #10565-042) with 3 μM CHIR99021 (FUJIFILM Wako Pure Chemical; cat. #034-23103), 5 μM SU5402 (FUJIFILM Wako Pure Chemical; cat. #193-16733), and N2-supplement (100×, Life Technologies; cat. #17502-048) for 3–4 days in a 90-mm low-adhesion culture dish (Corning, NY, USA). Subsequently, aggregates were cultured in the control maturation medium (DMEM/F12 with GlutaMAX [Life Technologies; cat. #10565-042], N2 supplement [Life Technologies; cat. #17502-048]; 10% FBS [Gibco, Waltham, MA, USA; cat. #10270-106], 100 μM taurin [Sigma-Aldrich; cat. #T8691-25G], and penicillin-streptomycin-amphotericin B suspension [FUJIFILM Wako Pure Chemical; cat. #161-23181]). The medium was exchanged every 3–4 days until use at approximately dd60.

Laser-induced retinal degeneration model

A laser-induced retinal degeneration model was established using the 577-nm OPSL laser system (PASCAL Streamline yellow; Topcon, Tokyo, Japan). The laser was used for selective photocoagulation of the ONL using a laser contact lens (Macula lens 901; Haag-Streit International, Köniz, Switzerland) with a magnification of 0.96×. The pattern scan consisted of nine spots arranged on a 3 × 3 grid. The parameters for the laser procedure included a spot spacing of 0 μm, laser size of 100 μm, and duration of 15 ms. We evaluated the photocoagulated area immediately after the laser with Spectral Domain OCT (SD-OCT) (RS3000; NIDEK, Gamagori, Japan) and a fundus camera (CX-1; Canon, Tokyo, Japan) and added the laser if deemed necessary.

Procedure of transplantation

Surgery was performed as described in a previous clinical study by Hirami et al.[8] Localized retinal detachment was created in the laser area by subretinal injection of oxyglutathione intraocular irrigating solution (BSS Plus; Alcon, Vernier, Switzerland) using a 38GPolyTip Cannula (MedOne, Sarasota, FL, USA), which was enlarged with scissors (Pinnacle 360 25G Vertical Scissors; Synergetics, MO, USA) for graft insertion using a customized 24-gauge plastic cannula. Another hole was created within the retinal detachment to allow smooth graft insertion without increasing subretinal pressure. Grafts were transplanted into the subretinal space using 1:6 sodium hyaluronate and chondroitin sulfate sodium (Viscoat; Alcon)/BSS Plus and spread throughout the target area with subretinal forceps (Keeler, Windsor, UK). Perfluoro-*n*-octane (Perfluoron; Alcon) was injected to drain the subretinal fluid, followed by the injection of silicone oil (Alcon) after fluid-air exchange.

Immunosuppression

Immunosuppression was achieved through STTA injections (KENACORT-A 40 mg/mL, 0.5 mL, Bristol Myers Squibb, Anagni, Italy) administered at the end of the transplantation surgery, 2 weeks post transplantation, and at the time of silicone oil removal. STTA injections were also administered when transplant rejection was suspected, with subsequent administration every 2 months. Cyclosporine (Sandimmun; 10 mg/mL, 7–9 mL, Novartis, Stein, Switzerland) was administered orally for systemic immunosuppression to maintain serum concentrations between 70 and 1,000 ng/mL. A subcutaneous cyclosporine injection (0.5 mg/mL) was administered when oral administration was challenging. The immunosuppression management protocol is shown in Figure S7.

FMERG recordings

FMERG was performed using an ER-80 system (Kowa, Nagoya, Japan) and a Burian-Allen bipolar contact lens electrode (Hansen Ophthalmic Development Laboratory, Bellingham, WA, USA) with a ground electrode placed on the ear for recording. Recordings were obtained from the photocoagulated area using the following parameters: stimulus light intensity of 39 cd/m^2, background light intensity of 1.28 cd/m^2, stimulus duration of 100 ms, and a 10° stimulus spot. The laser-induced retinal degeneration model was created using a stimulus repetition rate of 5 Hz and the average of 200 responses recorded using a Neuropack MEB-2200 system (Nihon Kohden, Tokyo, Japan). For assessments conducted 6 months after transplantation, the stimulus repetition rate was 4.5 Hz, and 2,000 responses were averaged using a PuREC system (Mayo, Inazawa, Japan). At each time point, FMERG recordings were performed twice in each session. For the analysis, all individual measurements were plotted in Figures 2I–2K, S9B, and S9C, whereas the mean value of the two recordings was used in Figures 2L, 2M, and S9A. Recordings with obvious noise or artifacts were excluded from the analysis.

Fluorescein angiography

Fluorescein angiography was performed monthly using the CX-1. Early phase indicates approximately 1 min after intravenous fluorescein injection, whereas late phase indicates approximately 10 min after injection.

IHC

The eyes were immediately harvested upon euthanization, fixed in SUPERFIX (Kurabo, Osaka, Japan) for 1 week, and transferred to PBS without calcium and magnesium. They were subsequently embedded in paraffin and sectioned at 10-μm thickness using an automated slide-preparation system (AS-200; Kurabo). Four types of antigen retrieval method were used after deparaffinization depending on the antibody used: (1) microwave treatment for 20 min in 10 mM citrate buffer (pH 6.0) at 100°C; (2) autoclave treatment for 20 min in 10 mM citrate buffer (pH 6.0) at 120°C; (3) autoclave treatment for 20 min in 10 mM Tris-EDTA (pH 9.0) at 120°C; and (4) enzymatic treatment for 10–15 min using 10 μg/mL proteinase K in 50 mM Tris buffer (pH 8.0) at 37°C. Specimens were blocked with blocking solution (1% BSA in PBS-0.3% Triton X-100) for 1 h at room temperature, followed by incubation with primary antibodies (Table S3) overnight at 4°C and secondary antibodies for 1 h at room temperature. Images were acquired using an LSM 700 confocal microscope (Carl Zeiss, Jena, Germany) and TCS SP8 confocal microscopy platform (Leica, Wetzlar, Germany). The primary antibodies used are listed in Table S3.

EM

The cornea and lens were removed from the transplanted eye of monkey 5 and fixed in 2% glutaraldehyde in 0.1 M cacodylate buffer at room temperature for 3 h. The transplantation area was trimmed and embedded in agarose gel to prepare 300-μm sections using a vibratome (DSK-Pro7; Dosaka EM, Kyoto, Japan) and stained with DAPI for observation under a fluorescence microscope to confirm CRX::Venus-positive graft integration in the host retina. The sections were imaged using the TCS SP8 confocal microscopy platform and stored in 4% glutaraldehyde in 0.1 M sodium cacodylate buffer until use. The sections were washed five times with 0.1 M cacodylate buffer, followed by fixation in 1% osmium tetroxide (OsO_4) in 0.1 M cacodylate buffer at 4°C for 1 h and subsequent washing five times with distilled water. The specimens were incubated overnight in 1% uranyl acetate and washed five more times with distilled water. The specimens were dehydrated in a graded ethanol series (20%, 50%, 70%, 90%, 99.5%, and 100%, each for 5 min on ice), followed by 100% acetone for 10 min at room temperature. Infiltration with Epon812 (TAAB, Aldermaston, Berkshire, UK) ensued, and polymerization was performed at 60°C for 72 h. The samples were sectioned at a thickness of 250 nm using an ultramicrotome (ULTRACUT-UCT; Leica) equipped with a diamond knife (Diatome, Nidau, Switzerland), stained with uranyl acetate and lead nitrate, and observed under a field-emission scanning electron microscope (MERLIN VP compact; Carl Zeiss) at 5 kV using a backscattered electron detector.

SBFSEM

One of the vibratome sections was shipped to the National Institute for Physiological Sciences, Okazaki in a temperature-controlled container for SBFSEM. The retinal pieces were incubated with 1.5% potassium ferrocyanide and 2% OsO_4 in PBS for 1 h. The tissue was placed in a freshly made thiocarbohydrazide (TCH) solution (0.1 g TCH in 10 mL dH_2O heated to 65°C for 1 h) for 20 min at room temperature after washing. After rinsing at room temperature, the tissue was incubated in 2% OsO_4 at room temperature for 30 min, rinsed again, and stained *en bloc* in 2% uranyl acetate overnight at 4°C. After another wash, the tissue was stained with Walton's lead aspartate (0.66% lead in 0.03 M L-aspartic acid, pH 5.0–5.5) at 50°C for 2 h. The retinal pieces were dehydrated in a graded ice-cold alcohol series and embedded in Durcupan resin after the final wash. Light microscopy images were acquired from semi-thin sections of the resin-embedded tissue surface, and the resin block was trimmed to retain the retinal region with signals overlapping with fluorescent signals of the grafted GFP-positive cells. SBFSEM observations of the retinal region were performed using a Merlin scanning

electron microscope (Carl Zeiss) equipped with a 3View in-chamber ultramicrotome system (Gatan, Pleasanton, CA, USA). Serial electron micrographs were acquired at thicknesses of 60 nm. Each image consisted of a 4 × 7 tiled montage, with individual tiles measuring 6,144 × 6,144 pixels at a spatial resolution of 5 nm per pixel. The image stacks were stitched, concatenated, and aligned using TrakEM2 (ImageJ/Fiji). Retinal cells were manually segmented and reconstructed using TrakEM2.

Measurement of BC length

The lengths of the BC dendrites were quantified from *z*-stack images of sectioned retinas from monkeys subjected to laser treatment (Figure 1) and monkeys 3, 4, and 6 (Figure 5). Orthogonal sections of the intersecting grafts were resliced in ImageJ, and the dendrites of the BCs were traced manually. The BCs were found in 3–4 separate sections, and the length from the base of the INL to the dendrite tip was measured for each BC.

Measurement of graft thickness and brightness of CRX::Venus

Graft thickness was measured from the same area in each monkey using OCT (Figure S4J). The longest graft length was measured in each monkey. OCT images were quantified using ImageJ, FA images of the grafts were captured using CX-1 (Figure S4I), and the mean brightness of CRX::Venus expression in the grafts was quantified using ImageJ software.

Measurement of cone ratio

The cone ratio was measured using GFP and NRL staining (Figure 4A). GFP was expressed in graft photoreceptors, whereas NRL was expressed in rod-shaped photoreceptors. The cone ratio was calculated as follows:

$$\text{Cone ratio} = (\text{GFPpos} - \text{NRLpos})/(\text{GFPpos}), \qquad \text{(Equation 1)}$$

where GFPpos represents the number of GFP-positive photoreceptors, and NRLpos represents the number of NRL-positive photoreceptors.

Four sections from separate areas of each monkey were stained, and all rosettes were manually counted in the sectioned retina using ImageJ.

To further validate this quantification method, cone proportion was also assessed using Cone Arrestin and CRX::Venus immunostaining, defined as Cone Arrestin-positive/CRX-positive cells in one representative eye (monkey 6; Figure S13). The cone proportion by Cone Arrestin-based quantification did not differ significantly from that obtained by NRL-based quantification ($p = 0.22$, Wilcoxon rank-sum test), supporting the validity of our NRL-based quantification.

Measurement of photoreceptor ratio

The photoreceptor ratio was measured using GFP and STEM121 staining (Figure S10). GFP was expressed in the graft photoreceptors, whereas STEM121 was expressed in all graft cells. The photoreceptor ratio was calculated as follows:

$$\text{PRC ratio} = (\text{GFPpos})/(\text{STEM121pos}), \qquad \text{(Equation 2)}$$

where GFPpos represents the number of GFP-positive photoreceptors, and STEM121pos represents the number of STEM121-positive photoreceptors.

Nine to 18 sections from separate areas in each monkey were stained. Blood vessels were removed after converting the image to 8-bit format. GFP and STEM121 were binarized, and the areas were measured using ImageJ software.

Evaluation of synaptic contact between host BC and graft photoreceptor

Synaptic contact was evaluated using GFP, STEM121, and PKCα staining (Figure 4D). Host and graft BCs were distinguished using STEM121 staining. Eleven to 12 sections from separate areas of monkeys 1, 2, 3, and 6 were stained. Two investigators independently evaluated synaptic contacts using a three-point grading scale. Synaptic contacts were graded as poor (no contact), fair (partial contact), or good (direct contact).

Statistical analysis

Statistical analyses were conducted using R software (version 4.1.2; https://cran.r-project.org/bin/macosx/). The exact Wilcoxon rank-sum test was used for most comparisons, whereas Fisher's exact test was used for synaptic contact analysis. Statistical significance was defined as $p < 0.05$. When multiple comparisons were performed, p values were adjusted using Bonferroni correction.

DATA AND CODE AVAILABILITY

The datasets supporting the current study have not been deposited in a public repository due to their large sizes but are available from the corresponding author upon reasonable request. Additional information required to reanalyze the data is available upon request.

ACKNOWLEDGMENTS

Animal experiments were conducted at the RIKEN BDR with the support of Hiroshi Kiyonari. We thank Masayo Takahashi, Satoshi Shirae, and Toshika Senba at Vision Care inc. for their professional support in animal experiments, Michiru Matsumura and Mitsuhiro Nishida at Kobe City Eye Hospital for supporting cell culture of human ESC and ROs, and Atsuko Imai and Nobuko Hattori at the National Institute for Physiological Sciences for the SBFSEM analysis. We would like to thank Editage (www.editage.jp) for English language editing. This research was funded by the National Institutes of Health (NIH) grant 1U24EY033272 (Y.F. and M.M.), JSPS KAKENHI grant 23K15925 (R.A.), and Beyer Retina Award 2022 (R.A.). This work was partly supported by JSPS KAKENHI grant no. JP22H04926 (N.O.), a Grant-in-Aid for Transformative Research Areas—Platforms for Advanced Technologies and Research Resources "Advanced Bioimaging Support," and Cooperative Study Programs of National Institute for Physiological Sciences (R.A.).

AUTHOR CONTRIBUTIONS

Conceptualization, A.O., Y.F., and M.M.; data curation, A.O., R.A., S.Y., and M.M.; formal analysis, A.O., A.K., R.A., and M.M.; funding acquisition, Y.F., R.A., N.O., and M.M.; investigation, A.O., K.K., A.K., R.A., D.P., N.O., S.O., K.O., S.Y., S.I., Y.K., and M.M.; methodology, R.A., N.O., T.M., S.Y., M.K., Y.K., and M.M.; project administration, M.M.; resources, M.K., T.M., S.Y., M.K., and M.M.; supervision, M.M.; visualization, R.A. and A.O.; writing – original draft, A.O. and R.A.; writing – review and editing, Y.F. and M.M.

DECLARATION OF INTERESTS

The authors declare no competing interests.

SUPPLEMENTAL INFORMATION

Supplemental information can be found online at https://doi.org/10.1016/j.ymthe.2026.06.037.

REFERENCES

1. Marc, R.E., Jones, B.W., Watt, C.B., and Strettoi, E. (2003). Neural remodeling in retinal degeneration. Prog. Retin. Eye Res. *22*, 607–655. https://doi.org/10.1016/s1350-9462(03)00039-9.
2. Pfeiffer, R.L., Marc, R.E., and Jones, B.W. (2020). Persistent remodeling and neurodegeneration in late-stage retinal degeneration. Prog. Retin. Eye Res. *74*, 100771. https://doi.org/10.1016/j.preteyeres.2019.07.004.
3. Mandai, M., Fujii, M., Hashiguchi, T., Sunagawa, G.A., Ito, S.-I., Sun, J., Kaneko, J., Sho, J., Yamada, C., and Takahashi, M. (2017). IPSC-derived retina transplants improve vision in rd1 end-stage retinal-degeneration mice. Stem Cell Rep. *8*, 69–83. https://doi.org/10.1016/j.stemcr.2016.12.008.
4. Mandai, M. (2023). Pluripotent stem cell-derived retinal organoid/cells for retinal regeneration therapies: A review. Regen. Ther. *22*, 59–67. https://doi.org/10.1016/j.reth.2022.12.005.
5. Ozaki, A., Sakai, D., and Mandai, M. (2025). hPSC-based treatment of retinal diseases - Current progress and challenges. Adv. Drug Deliv. Rev. *221*, 115587. https://doi.org/10.1016/j.addr.2025.115587.
6. Akiba, R., Tu, H.-Y., Hashiguchi, T., Takahashi, Y., Toyooka, K., Tsukamoto, Y., Baba, T., Takahashi, M., and Mandai, M. (2024). Host-graft synapses form functional microstructures and shape the host light responses after stem cell-derived retinal sheet transplantation. Invest. Ophthalmol. Vis. Sci. *65*, 8. https://doi.org/10.1167/iovs.65.12.8.
7. Watanabe, M., Yamada, T., Tu, H.-Y., Chaya, T., Okayama, S., Onoue, K., Yonemura, S., Koike, C., Tachibana, M., Furukawa, T., and Mandai, M. (2025). Graft-derived horizontal cells contribute to host-graft synapses in degenerated retinas after retinal organoid transplantation. Stem Cell Rep. *20*, 102545. https://doi.org/10.1016/j.stemcr.2025.102545.
8. Hirami, Y., Mandai, M., Sugita, S., Maeda, A., Maeda, T., Yamamoto, M., Uyama, H., Yokota, S., Fujihara, M., Igeta, M., et al. (2023). Safety and stable survival of stem-cell-derived retinal organoid for 2 years in patients with retinitis pigmentosa. Cell Stem Cell *30*, 1585–1596.e6. https://doi.org/10.1016/j.stem.2023.11.004.
9. Shirai, H., Mandai, M., Matsushita, K., Kuwahara, A., Yonemura, S., Nakano, T., Assawachananont, J., Kimura, T., Saito, K., Terasaki, H., et al. (2016). Transplantation of human embryonic stem cell-derived retinal tissue in two primate models of retinal degeneration. Proc. Natl. Acad. Sci. USA *113*, E81–E90. https://doi.org/10.1073/pnas.1512590113.
10. Tu, H.-Y., Watanabe, T., Shirai, H., Yamasaki, S., Kinoshita, M., Matsushita, K., Hashiguchi, T., Onoe, H., Matsuyama, T., Kuwahara, A., et al. (2019). Medium- to long-term survival and functional examination of human iPSC-derived retinas in rat and primate models of retinal degeneration. EBioMedicine *39*, 562–574. https://doi.org/10.1016/j.ebiom.2018.11.028.
11. Iwama, Y., Sugase-Miyamoto, Y., Onoue, K., Uyama, H., Matsuda, K., Hayashi, K., Akiba, R., Masuda, T., Yokota, S., Yonemura, S., et al. (2024). Transplantation of human pluripotent stem cell-derived retinal sheet in a primate model of macular hole. Stem Cell Rep. *19*, 1524–1533. https://doi.org/10.1016/j.stemcr.2024.09.002.
12. Sethi, C.S., Lewis, G.P., Fisher, S.K., Leitner, W.P., Mann, D.L., Luthert, P.J., and Charteris, D.G. (2005). Glial remodeling and neural plasticity in human retinal detachment with proliferative vitreoretinopathy. Invest. Ophthalmol. Vis. Sci. *46*, 329–342. https://doi.org/10.1167/iovs.03-0518.
13. Sullivan, R.K.P., Woldemussie, E., and Pow, D.V. (2007). Dendritic and synaptic plasticity of neurons in the human age-related macular degeneration retina. Invest. Ophthalmol. Vis. Sci. *48*, 2782–2791. https://doi.org/10.1167/iovs.06-1283.
14. Eliasieh, K., Liets, L.C., and Chalupa, L.M. (2007). Cellular reorganization in the human retina during normal aging. Invest. Ophthalmol. Vis. Sci. *48*, 2824–2830. https://doi.org/10.1167/iovs.06-1228.
15. Akiba, R., Lind Boniec, S., Knecht, S., Uyama, H., Tu, H.-Y., Baba, T., Takahashi, M., Mandai, M., and Wong, R.O. (2024). Cellular and circuit remodeling of the primate foveal midget pathway after acute photoreceptor loss. Proc. Natl. Acad. Sci. USA *121*, e2413104121. https://doi.org/10.1073/pnas.2413104121.
16. Matsuyama, T., Tu, H.-Y., Sun, J., Hashiguchi, T., Akiba, R., Sho, J., Fujii, M., Onishi, A., Takahashi, M., and Mandai, M. (2021). Genetically engineered stem cell-derived retinal grafts for improved retinal reconstruction after transplantation. iScience *24*, 102866. https://doi.org/10.1016/j.isci.2021.102866.
17. Yamasaki, S., Tu, H.-Y., Matsuyama, T., Horiuchi, M., Hashiguchi, T., Sho, J., Kuwahara, A., Kishino, A., Kimura, T., Takahashi, M., and Mandai, M. (2022). A Genetic modification that reduces ON-bipolar cells in hESC-derived retinas enhances functional integration after transplantation. iScience *25*, 103657. https://doi.org/10.1016/j.isci.2021.103657.
18. Watanabe, M., Yamada, T., Koike, C., Takahashi, M., Tachibana, M., and Mandai, M. (2025). Transplantation of genome-edited retinal organoids restores some fundamental physiological functions coordinated with severely degenerated host retinas. Stem Cell Rep. *20*, 102393. https://doi.org/10.1016/j.stemcr.2024.102393.
19. Grünert, U., and Martin, P.R. (2020). Cell types and cell circuits in human and non-human primate retina. Prog. Retin. Eye Res. *78*, 100844. https://doi.org/10.1016/j.preteyeres.2020.100844.
20. Dunn, F.A. (2015). Photoreceptor ablation initiates the immediate loss of glutamate receptors in postsynaptic bipolar cells in retina. J. Neurosci. *35*, 2423–2431. https://doi.org/10.1523/JNEUROSCI.4284-14.2015.
21. Kalloniatis, M., Nivison-Smith, L., Chua, J., Acosta, M.L., and Fletcher, E.L. (2016). Using the rd1 mouse to understand functional and anatomical retinal remodelling and treatment implications in retinitis pigmentosa: A review. Exp. Eye Res. *150*, 106–121. https://doi.org/10.1016/j.exer.2015.10.019.
22. Peng, Y.-R., Shekhar, K., Yan, W., Herrmann, D., Sappington, A., Bryman, G.S., van Zyl, T., Do, M.T.H., Regev, A., and Sanes, J.R. (2019). Molecular classification and comparative taxonomics of foveal and peripheral cells in primate retina. Cell *176*, 1222–1237.e22. https://doi.org/10.1016/j.cell.2019.01.004.
23. Sanjurjo-Soriano, C., Erkilic, N., Damodar, K., Boukhaddaoui, H., Diakatou, M., Garita-Hernandez, M., Mamaeva, D., Dubois, G., Jazouli, Z., Jimenez-Medina, C., et al. (2022). Retinoic acid delays initial photoreceptor differentiation and results in a highly structured mature retinal organoid. Stem Cell Res. Ther. *13*, 478. https://doi.org/10.1186/s13287-022-03146-x.
24. Okano, K., Maeda, A., Chen, Y., Chauhan, V., Tang, J., Palczewska, G., Sakai, T., Tsuneoka, H., Palczewski, K., and Maeda, T. (2012). Retinal cone and rod photoreceptor cells exhibit differential susceptibility to light-induced damage: Rods are more susceptible than cones to light-induced damage. J. Neurochem. *121*, 146–156. https://doi.org/10.1111/j.1471-4159.2012.07647.x.
25. Bringmann, A., Syrbe, S., Görner, K., Kacza, J., Francke, M., Wiedemann, P., and Reichenbach, A. (2018). The primate fovea: Structure, function and development. Prog. Retin. Eye Res. *66*, 49–84. https://doi.org/10.1016/j.preteyeres.2018.03.006.
26. Das, T., del Cerro, M., Jalali, S., Rao, V.S., Gullapalli, V.K., Little, C., Loreto, D.A., Sharma, S., Sreedharan, A., del Cerro, C., and Rao, G.N. (1999). The transplantation of human fetal neuroretinal cells in advanced retinitis pigmentosa patients: results of a long-term safety study. Exp. Neurol. *157*, 58–68. https://doi.org/10.1006/exnr.1998.6992.
27. Radtke, N.D., Aramant, R.B., Petry, H.M., Green, P.T., Pidwell, D.J., and Seiler, M.J. (2008). Vision improvement in retinal degeneration patients by implantation of retina together with retinal pigment epithelium. Am. J. Ophthalmol. *146*, 172–182. https://doi.org/10.1016/j.ajo.2008.04.009.
28. Yamasaki, S., Sugita, S., Horiuchi, M., Masuda, T., Fujii, S., Makabe, K., Kawasaki, A., Hayashi, T., Kuwahara, A., Kishino, A., et al. (2021). Low immunogenicity and immunosuppressive properties of human ESC- and iPSC-derived retinas. Stem Cell Rep. *16*, 851–867. https://doi.org/10.1016/j.stemcr.2021.02.021.
29. Uyama, H., Tu, H.-Y., Sugita, S., Yamasaki, S., Kurimoto, Y., Matsuyama, T., Shiina, T., Watanabe, T., Takahashi, M., and Mandai, M. (2022). Competency of iPSC-derived retinas in MHC-mismatched transplantation in non-human primates. Stem Cell Rep. *17*, 2392–2408. https://doi.org/10.1016/j.stemcr.2022.09.014.

30. Petrash, C.C., Palestine, A.G., and Canto-Soler, M.V. (2021). Immunologic rejection of transplanted retinal pigmented epithelium: Mechanisms and strategies for prevention. Front. Immunol. *12*, 621007. https://doi.org/10.3389/fimmu.2021.621007.

31. Fariss, R.N., Li, Z.Y., and Milam, A.H. (2000). Abnormalities in rod photoreceptors, amacrine cells, and horizontal cells in human retinas with retinitis pigmentosa. Am. J. Ophthalmol. *129*, 215–223. https://doi.org/10.1016/s0002-9394(99)00401-8.

32. Banin, E., Cideciyan, A.V., Alemán, T.S., Petters, R.M., Wong, F., Milam, A.H., and Jacobson, S.G. (1999). Retinal rod photoreceptor-specific gene mutation perturbs cone pathway development. Neuron *23*, 549–557. https://doi.org/10.1016/s0896-6273(00)80807-7.

33. Ishikura, M., Muraoka, Y., Hirami, Y., Tu, H.-Y., and Mandai, M. (2024). Adaptive optics optical coherence tomography analysis of induced pluripotent stem cell-derived retinal organoid transplantation in retinitis pigmentosa. Cureus *16*, e64962. https://doi.org/10.7759/cureus.64962.

34. Nakano, T., Ando, S., Takata, N., Kawada, M., Muguruma, K., Sekiguchi, K., Saito, K., Yonemura, S., Eiraku, M., and Sasai, Y. (2012). Self-formation of optic cups and storable stratified neural retina from human ESCs. Cell Stem Cell *10*, 771–785. https://doi.org/10.1016/j.stem.2012.05.009.

35. Kuwahara, A., Ozone, C., Nakano, T., Saito, K., Eiraku, M., and Sasai, Y. (2015). Generation of a ciliary margin-like stem cell niche from self-organizing human retinal tissue. Nat. Commun. *6*, 6286. https://doi.org/10.1038/ncomms7286.

YMTHE, Volume *34*

Supplemental Information

Long-term functional synaptic integration of genome-edited retinal organoids in a primate model of macular degeneration

Atsuta Ozaki, Akihiro Kawai, Ryutaro Akiba, Satoko Okayama, Nobuhiko Ohno, Keisuke Kajita, Tomohiro Masuda, Satoshi Yokota, Shin-ichiro Ito, Du Peiyan, Kenta Onoue, Shigenobu Yonemura, Mineo Kondo, Yasuo Kurimoto, Yingbin Fu, and Michiko Mandai

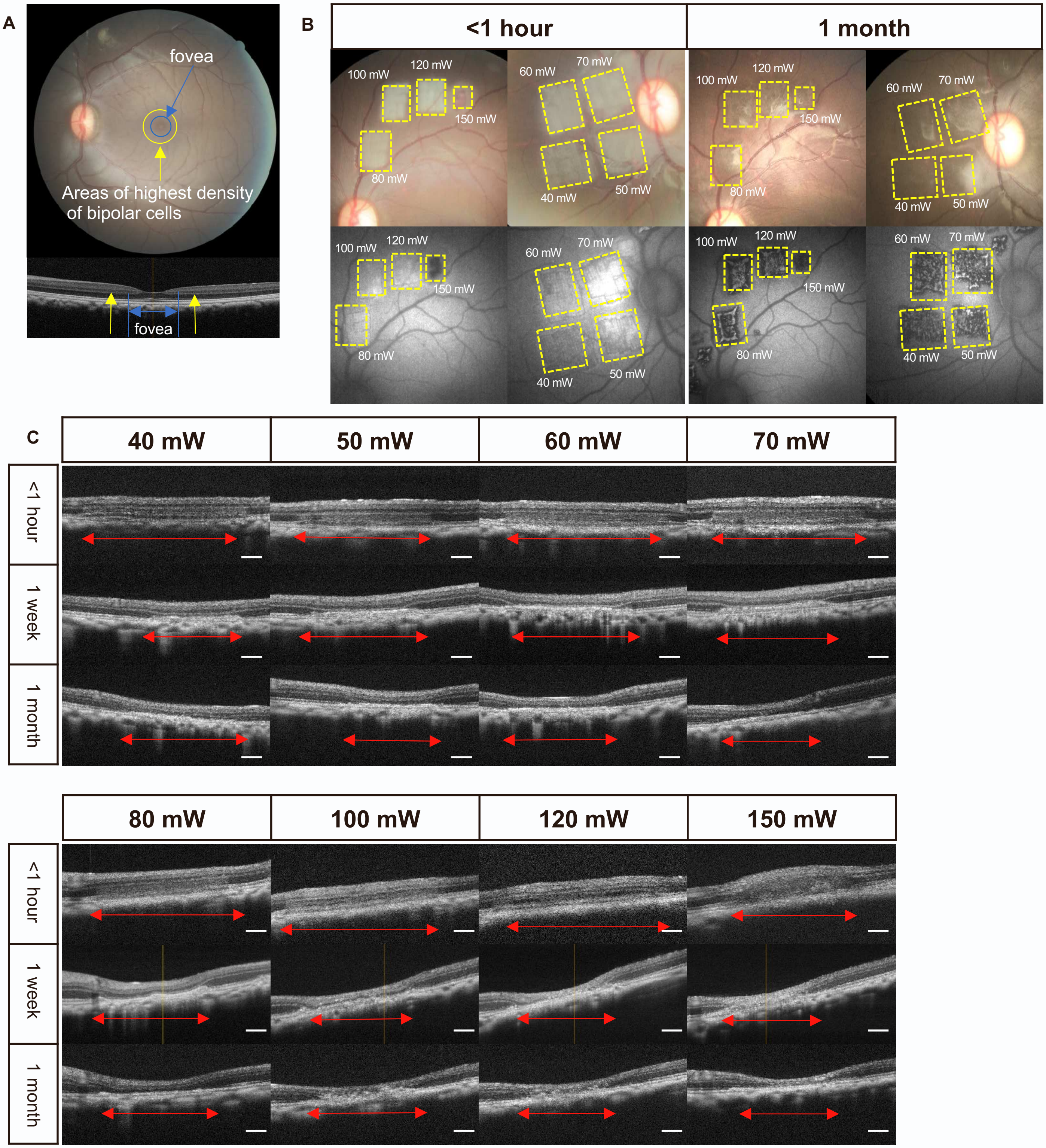

Figure S1. Optimization of laser power from 40 mW to 150 mW. (A) The eccentricity range of fovea is from 0.13 to 0.6 mm (blue circle). The eccentricity of the highest density of BCs are 1mm (yellow circle). (B) Representative fundus images of 80-150 mW (Upper left) and 40-70 mW (Upper right) and autofluorescence images of 80–150 mW (Lower left) and 40–70 mW (Lower right) taken < 1 hour and 1 month after laser. (C) Sectional views of OCT imaging of 40-150 mW < 1 hour, 1 week, and 1 month after laser. The laser area was indicated by red double-headed arrow. Scale bars: 300 μm

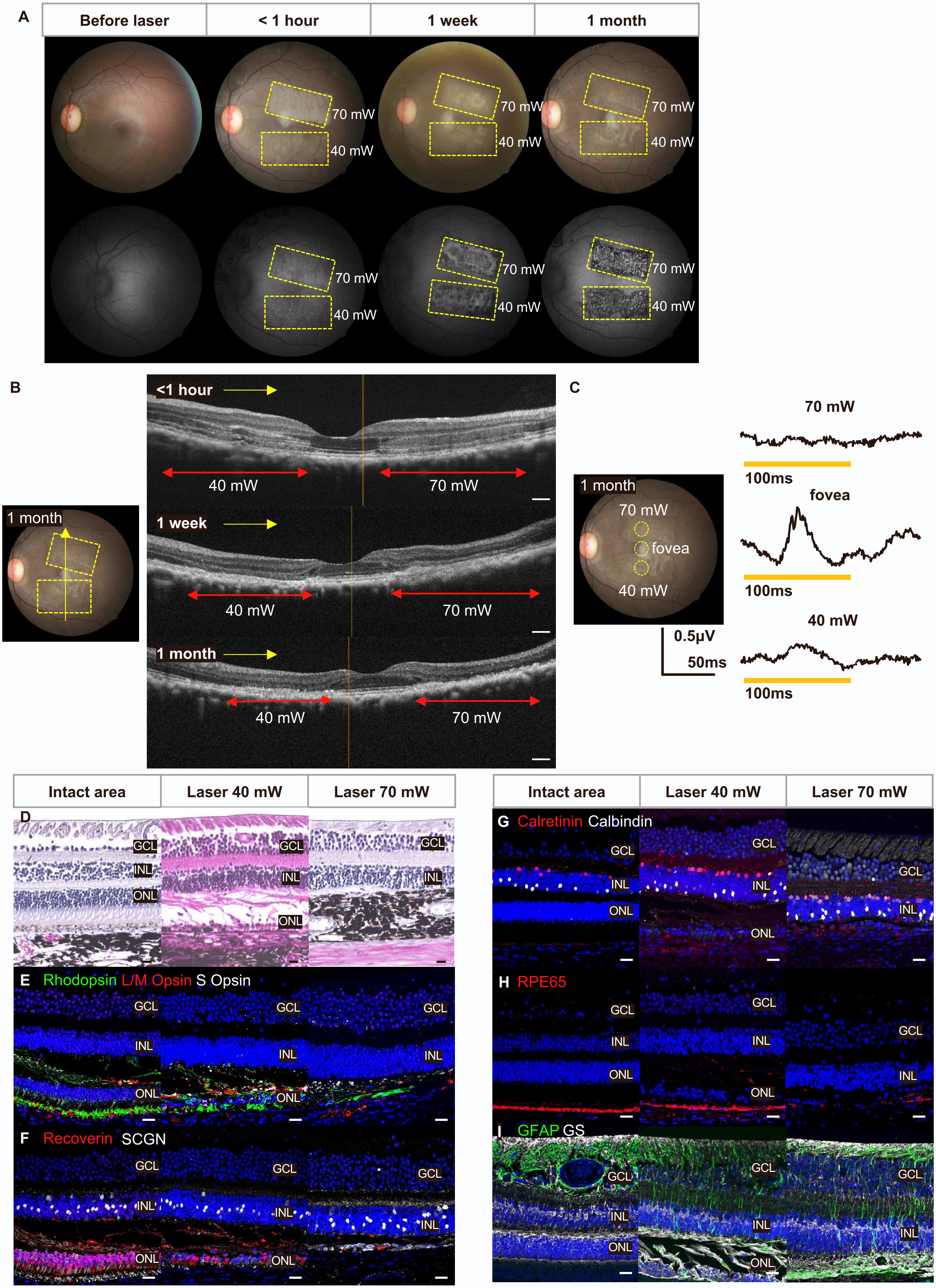
A
Before laser
< 1 hour
1 week
1 month
70 mW
40 mW
B
<1 hour
1 week
1 month
40 mW
70 mW
C
1 month
70 mW
fovea
40 mW
0.5μV
50ms
100ms
Intact area
Laser 40 mW
Laser 70 mW
D
GCL
INL
ONL
E Rhodopsin L/M Opsin S Opsin
F Recoverin SCGN
G Calretinin Calbindin
H RPE65
I GFAP GS

Figure S2. Laser-induced retinal degeneration monkey model involving para- and peri-fovea. **(A)** Representative fundus images (Upper) and autofluorescence images (Lower) taken before laser treatment, <1 hour, 1 week, and 1 month after laser treatment. The upper area from the fovea was lasered at 70 mW and the lower area at 40 mW (yellow dot). (B) Cross-sectional OCT images of the yellow line on the fundus, taken <1 hour, 1 week, and 1 month after laser treatment. The left side of the OCT was at 40 mW and the right side at 70 mW (red two-headed arrow) Scale bars: 300 μm (C) One month after laser, FMERG was stimulated at 70mW (upper), at the fovea (central), and at 40 mW (lower) (yellow dot circle). (D–I) Histological images of retinas compared with intact area, laser 40 mW, and laser 70 mW. (D) Representative images of Hematoxylin &Eosin stained retinal sections showing ONL loss of Laser 70mW. Scale bars: 20 μm (E–I) Retinal sections stained with Rhodopsin, L/M opsin and S opsin (E), Recoverin and SCGN (F), Calretinin and Calbindin (G), RPE65 (H), GFAP and GS (I). Scale bars: 25 μm

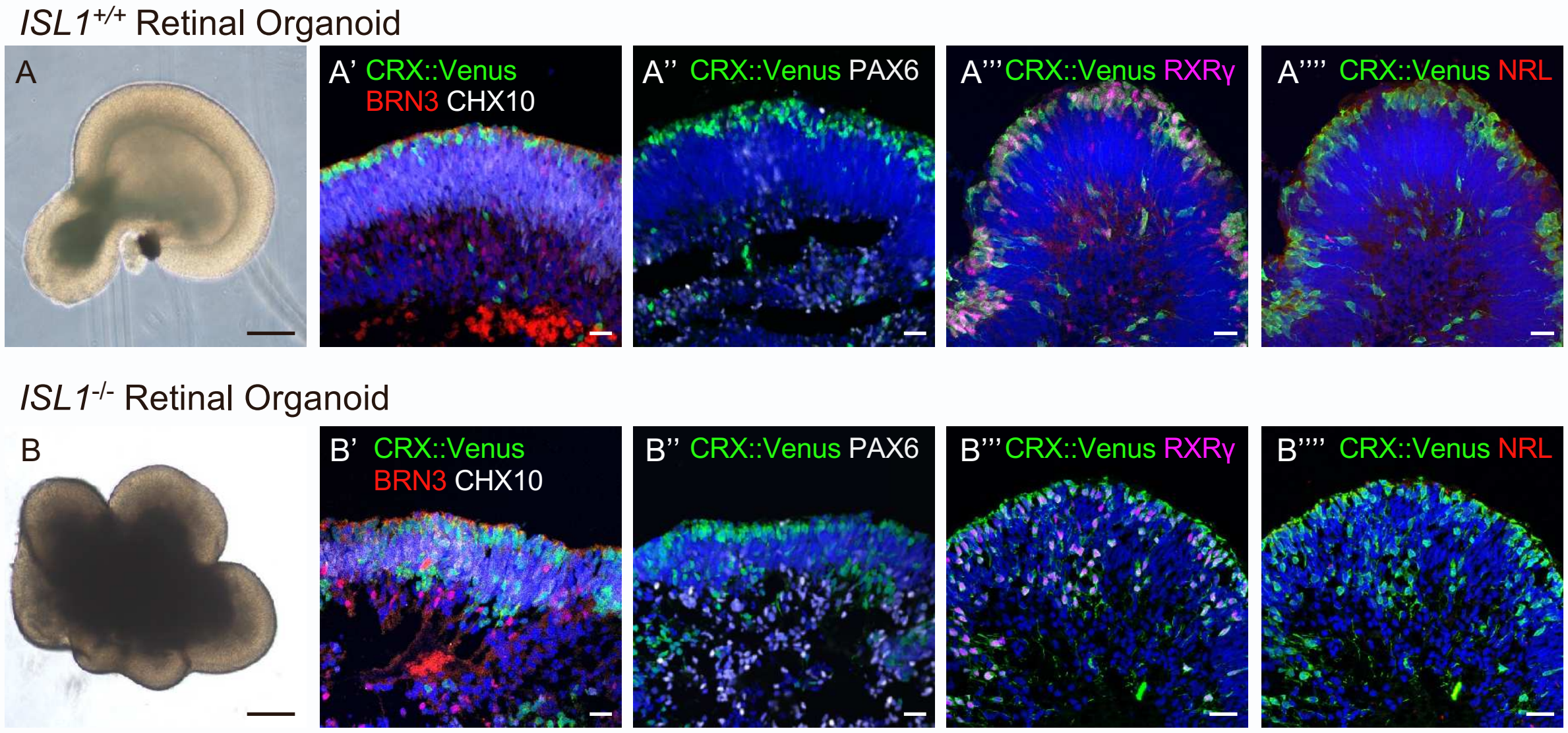

Figure S3. Human ESC-derived RO sheets at the time of transplantation. (A, B) Bright field images of *ISL1*$^{+/+}$ (A) and *ISL1*$^{-/-}$ (B) human ESC ROs (approximately DD60). Scale bars: 100 µm (A', B') Immunostaining of *ISL1*$^{+/+}$ (A') and *ISL1*$^{-/-}$ (B') human ESC retinal organoids for CRX::Venus, BRN3, and CHX10 (approximately DD60). (A'', B'') Immunostaining of *ISL1*$^{+/+}$ (A'') and *ISL1*$^{-/-}$ (B'') human ESC retinal organoids for CRX::Venus and PAX6. (A''', B''') Immunostaining of Immunostaining of *ISL1*$^{+/+}$ (A''') and *ISL1*$^{-/-}$ (B''') human ESC retinal organoids for CRX::Venus and RXRγ . (A'''', B'''') Immunostaining of *ISL1*$^{+/+}$ (A'''') and *ISL1*$^{-/-}$ (B'''') human ESC retinal organoids for CRX and NRL . Scale bars: 20 µm (A'–A'''', B'–B'''')

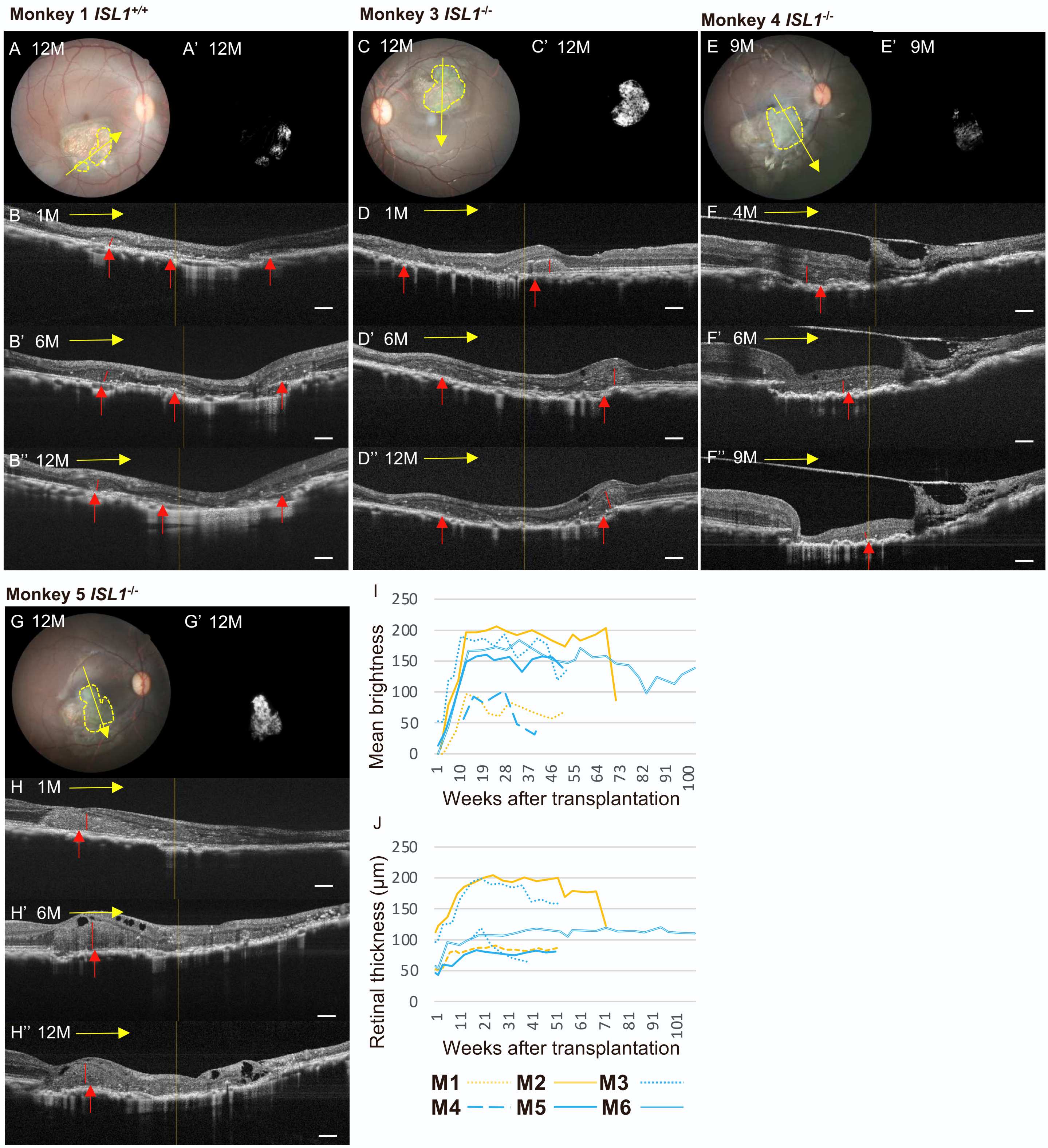

Figure S4. In vivo imaging of human ESC-derived RO sheets after transplantation. (A–H'') Representative fundus images (A, C, E, G), FA images of CRX::Venus (A', C', E', G'), and OCT images (B–B'', D–D'', F–F'', H–H'') of the transplanted grafts in the monkey eyes (Monkey 1, 3, 4, 5, respectively). Scale bars: 300µm (I) Line graph of temporal changes in Mean brightness of CRX::Venus for each transplanted eye. (J) Line graph of temporal changes in graft thickness for each transplanted eye.

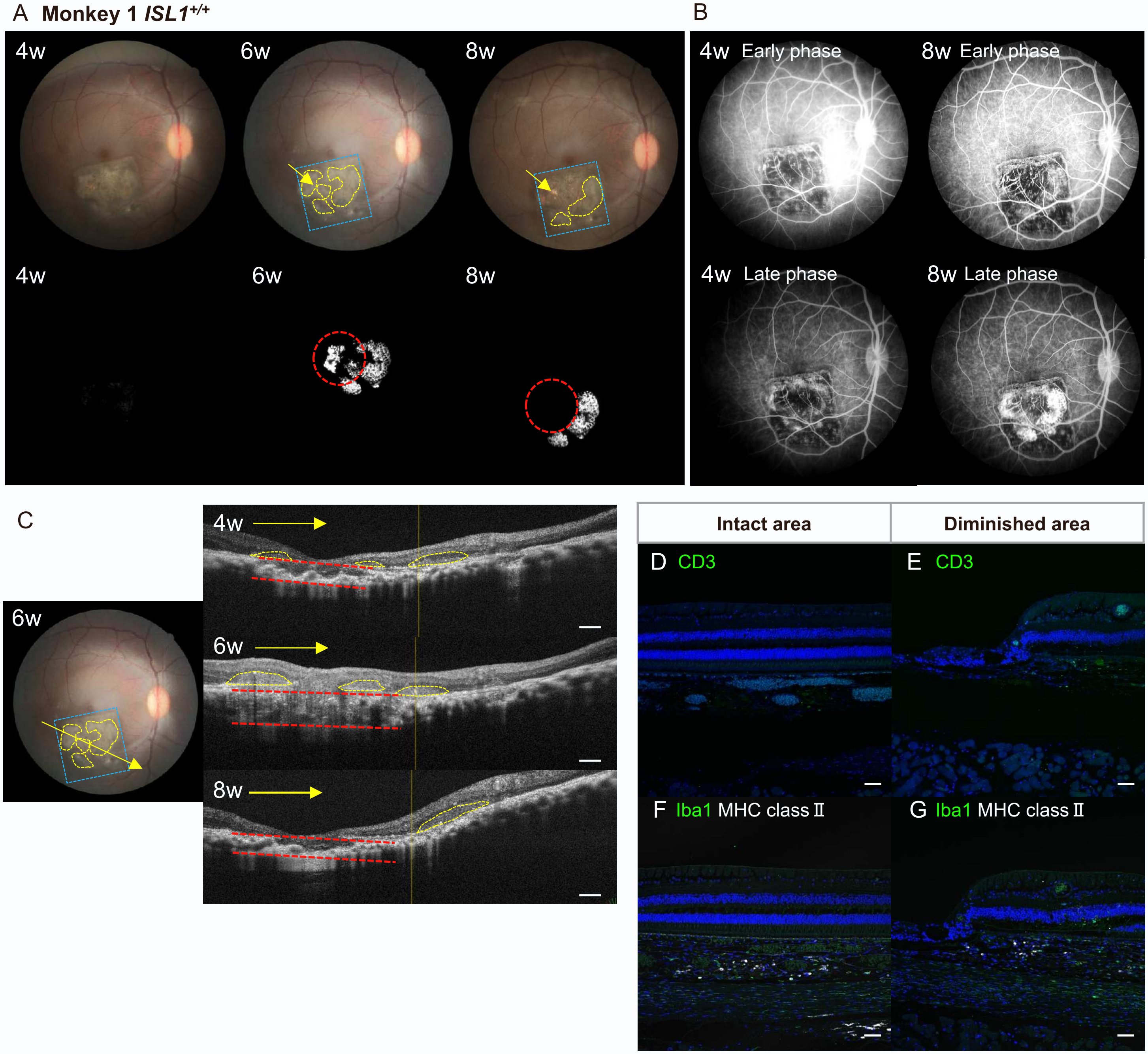

Figure S5. Evaluation of Monkey 1 before and after graft loss. (A) Representative fundus images of the transplanted graft (yellow dot) before and after graft loss (red dot circle). Laser area was indicated by light blue dot, insertion site was indicated by yellow arrow. (B) Representative FA images of the transplanted graft before (4w) and after (8w) graft loss. (C) Representative OCT images of the transplanted graft before (6w) and after (8w) graft loss. The grafts (yellow dot) were located at the laser area (light blue dot) Choroidal thickness was indicated by red dot line. Scale bars: 300μm (D–G) Retinal sections were stained with CD3 (D, E), Iba1 (F, G), and MHC class II (F, G) in the intact area and graft diminished area. Scale bars: 50 μm

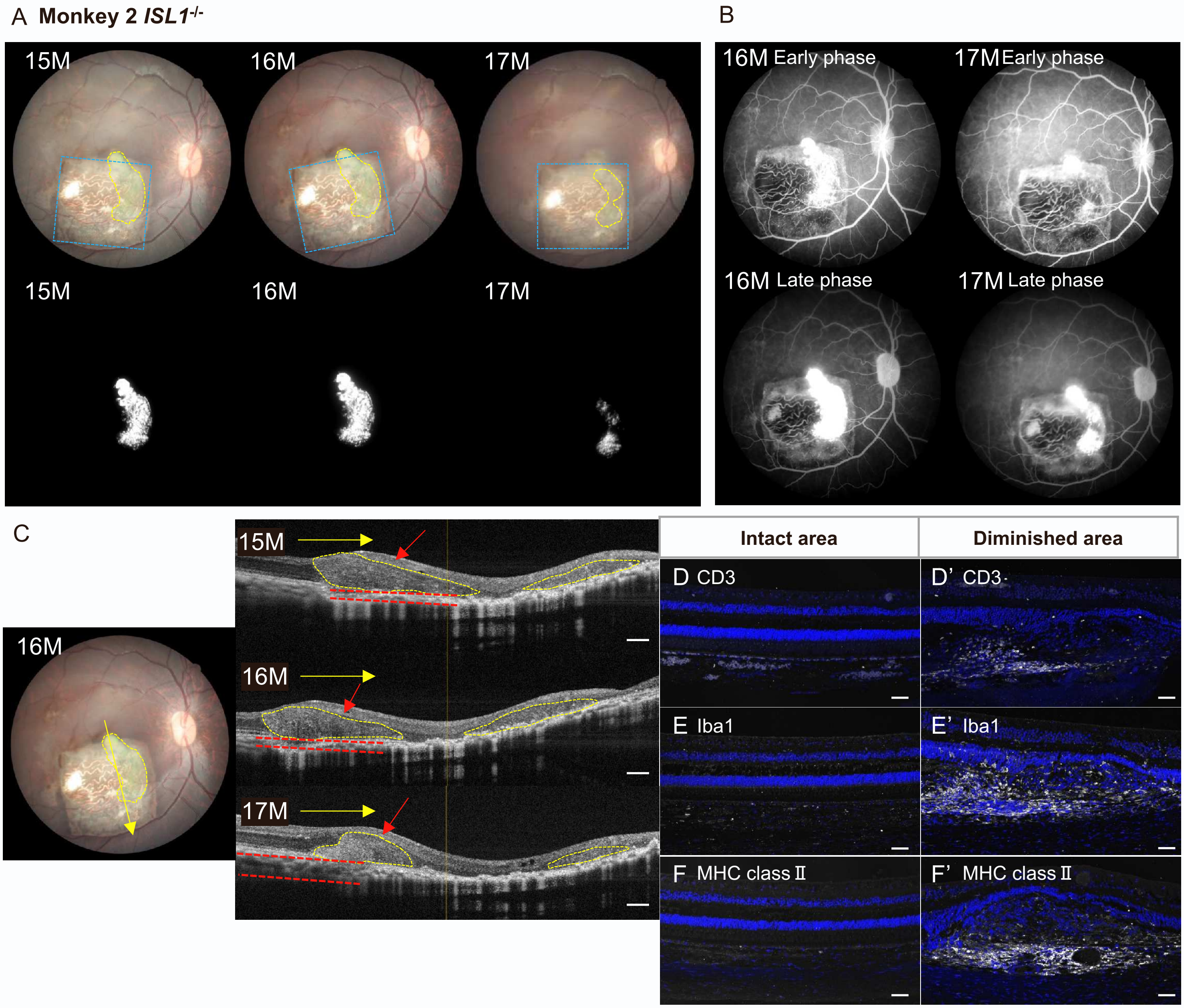

Figure S6. Evaluation of Monkey 2 before and after graft loss. (A) Representative fundus images of the transplanted graft (yellow dot) before and after graft loss. Laser area was indicated by light blue dot. (B) Representative FA images of the transplanted graft before (16M) and after (17M) graft loss. (C) Representative OCT images of the transplanted graft between 15M and 17M. The grafts (yellow dot) were located at the transplanted area, and the diminished area was indicated by red arrow. Red dot line showed the thickness of choroid vessel. Scale bars: 300 μm (D–F') Retinal sections were stained with CD3 (D, D'), Iba1 (E, E'), and MHC class II (F, F') in the intact area and graft diminished area. Scale bars: 50 μm

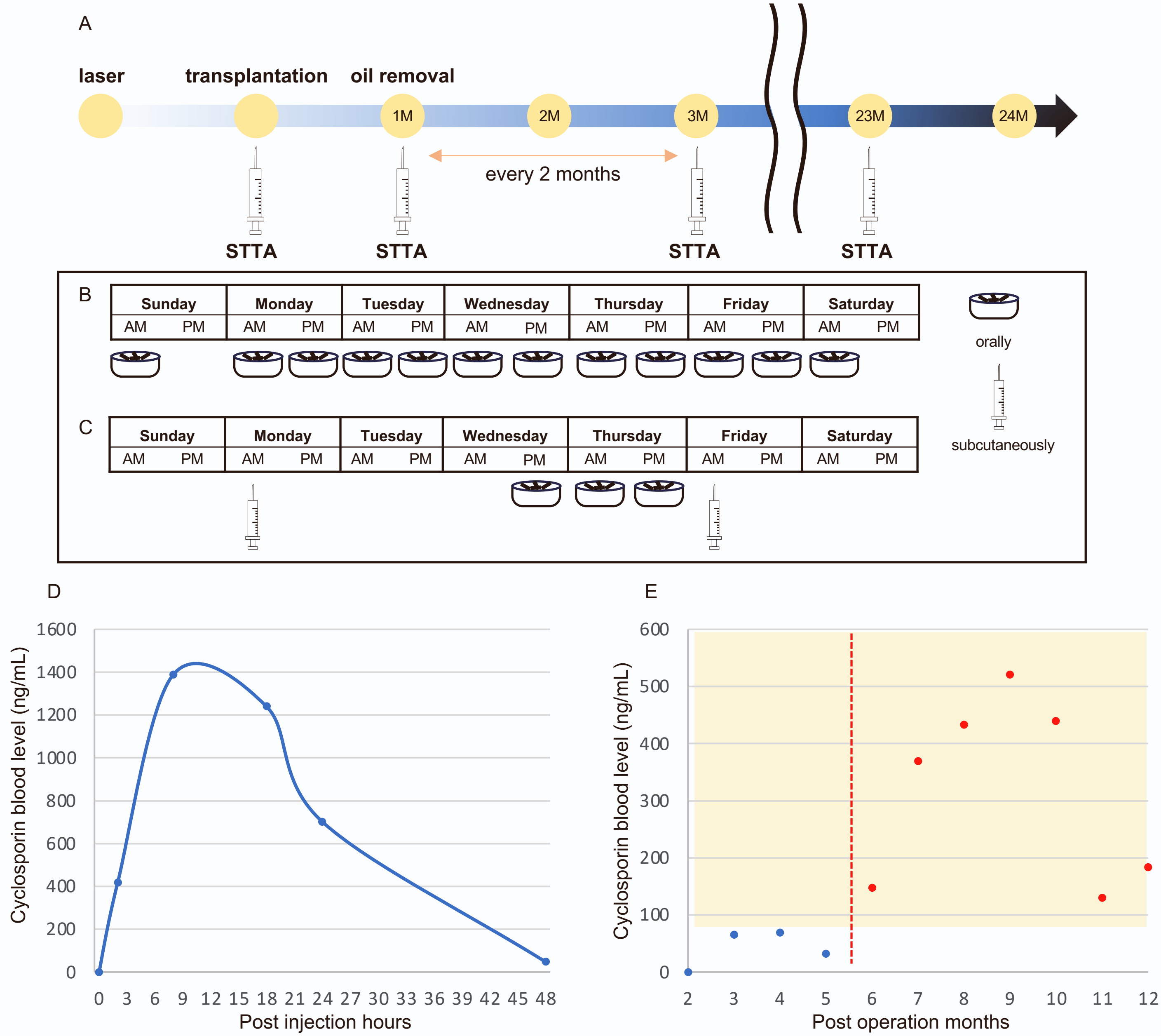

Figure S7. Management of local and systemic immunosuppression. (A) Local immunosuppressant injection protocol. STTA was administered at the time of transplantation and oil removal, and then every 2 months. (B) Systemic immunosuppression protocol of cyclosporin orally. (C) Systemic immunosuppression protocol of oral and subcutaneous administration of cyclosporin (D) Cyclosporine blood concentration profile after subcutaneous administration of cyclosporin. Cyclosporine concentrations were measured over time in four monkeys. (E) Changes in blood concentration of cyclosporine in monkey 3. Blue dots represent cyclosporin managed by only orally, whereas red dots represent cyclosporin managed subcutaneously and orally.

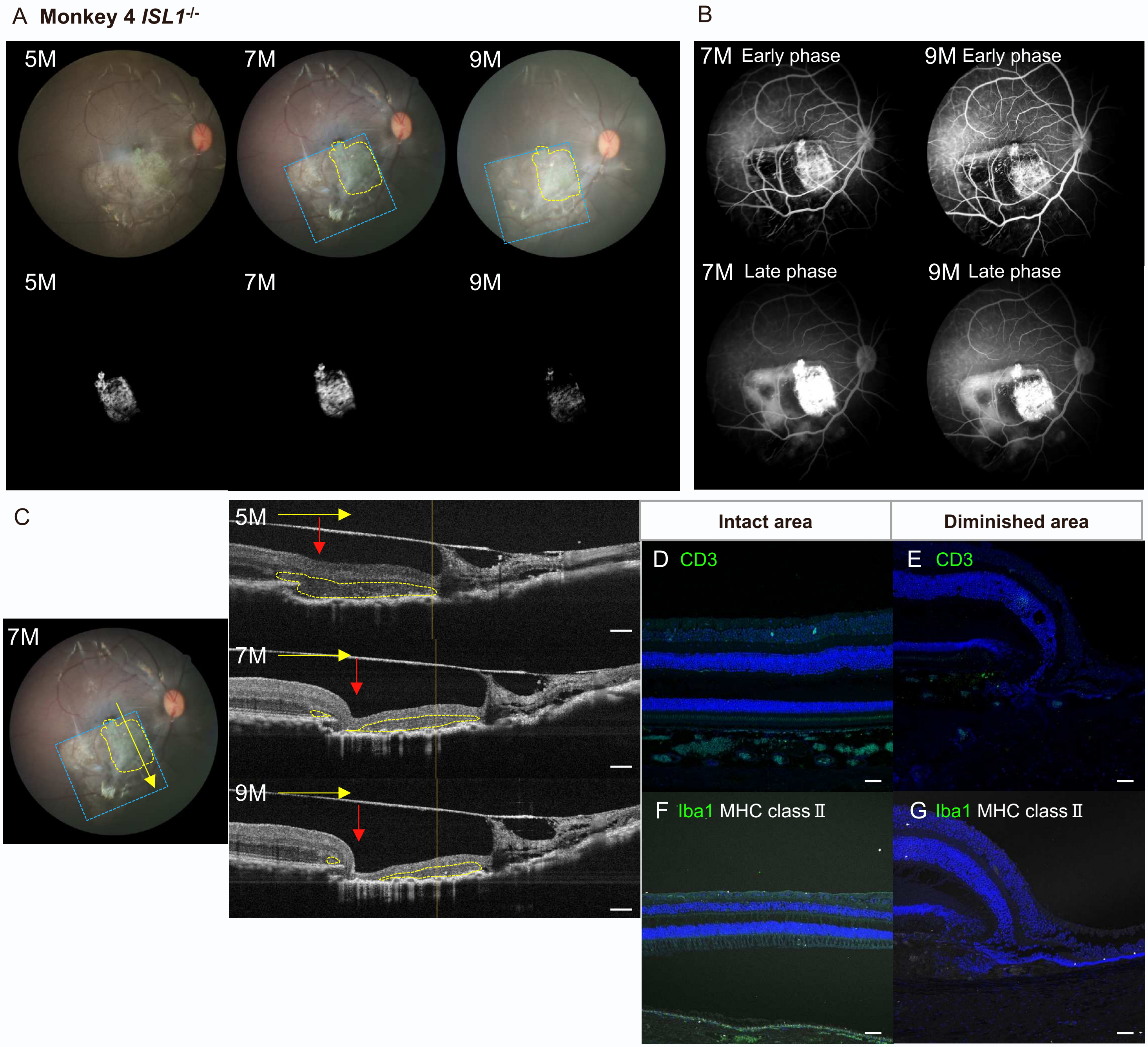

Figure S8. Evaluation of Monkey 4 before and after graft loss. (A) Representative fundus images of the transplanted graft (yellow dot) before and after graft loss. Laser area was indicated by light blue dot. (B) Representative FA images of the transplanted graft before (7M) and after (9M) graft loss. (C) Representative OCT images of the transplanted graft before (7M) and after (9M) graft loss. The grafts (yellow dot) were located at the laser area (light blue dot), and the diminished area was indicated by red arrow. Scale bars: 300 µm (D–G) Retinal sections were stained with CD3 (D, E), Iba1 (F, G), and MHC class II (F, G) in the intact area and diminished area. Scale bars: 50 µm

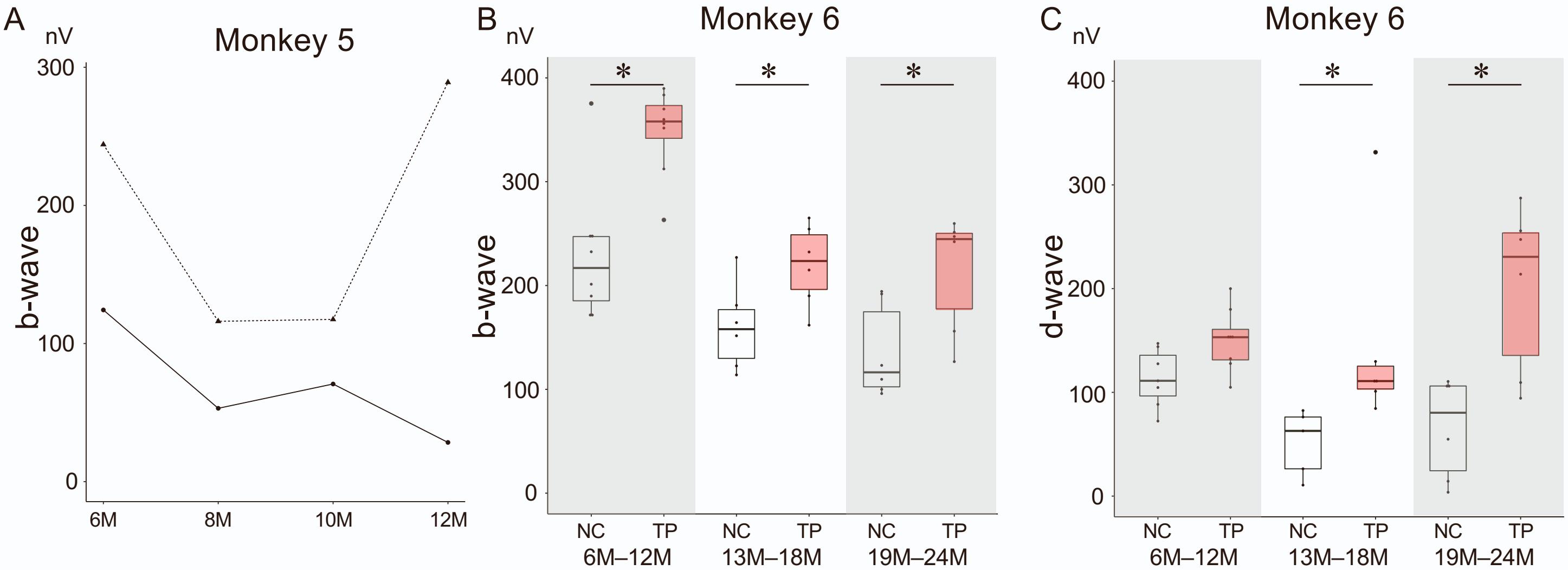

Figure S9. **Longitudinal comparison between grafted and non-grafted area in Monkey 5 and Monkey 6** (A) Longitudinal comparison of FMERG wave amplitudes between grafted and non-grafted retinal areas in Monkey 5. Dotted and solid lines indicate the longitudinal changes in b-wave amplitudes in grafted and non-grafted areas, respectively. (B, C) Comparison of FMERG wave amplitudes between non-grafted (NC) and grafted (TP) areas in Monkey 6 at 6 months–12 months, 13 months–18 months, and 19 months–24 months post transplantation for b-wave (B) and d-wave (C) (*Exact Wilcoxon rank sum test, $p < 0.05$).

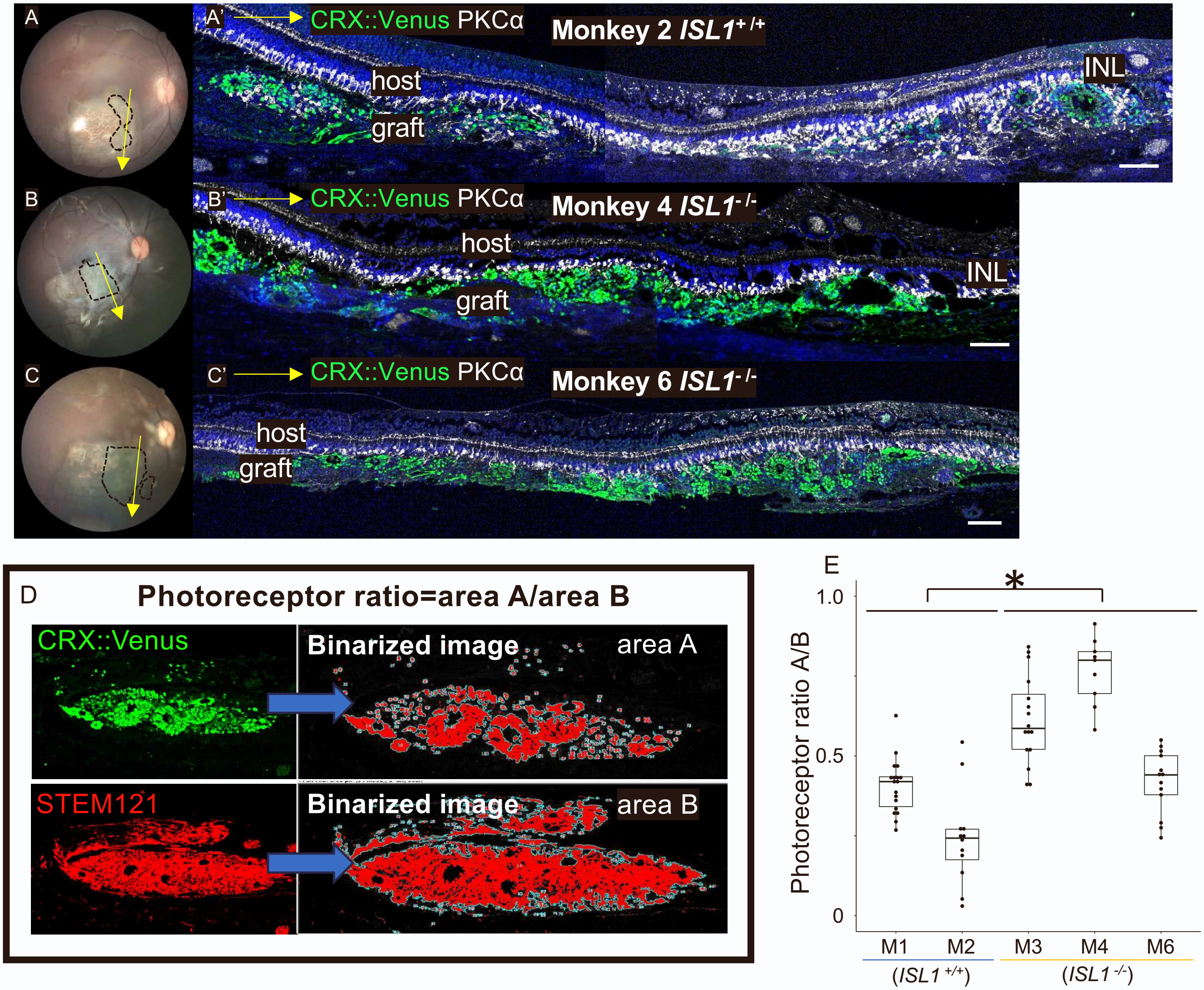

Figure S10. **Histological evaluation of the proportion of photoreceptor cells between *ISL1*$^{+/+}$ and *ISL1*$^{-/-}$.** (A–C') Immunostaining of monkey retinas transplanted with *ISL1*$^{+/+}$ (A) or *ISL1*$^{-/-}$ (B, C) RO sheets at 17 months (A), 9 months (B), and 24 months (C) post-transplantation. Graft photoreceptors were identified within CRX::Venus-positive rosettes in the graft area, and host bipolar cells were defined by their anatomical location within the host INL. In *ISL1*$^{-/-}$ grafts lacking graft-derived PKCα-positive ON-bipolar cells, all PKCα-positive dendrites extending from the host were therefore considered exclusively of host origin. Yellow arrows in the fundus images indicate the orientation of the retinal sections. Scale bars: 100 μm (D) Schematic representations of photoreceptors ratio with binarized images of CRX::Venus (graft photoreceptors) and STEM121 (graft cells). The photoreceptor ratio was defined as the area of CRX::Venus divided by the area of STEM121. (E) The photoreceptor ratio in the grafts of each monkey (monkey 1: 18 sections; monkey 2: 12 sections; monkey 3: 16 sections; monkey 4: 9 sections; monkey 6: 13 sections) (*Exact Wilcoxon rank sum test, $p < 0.05$).

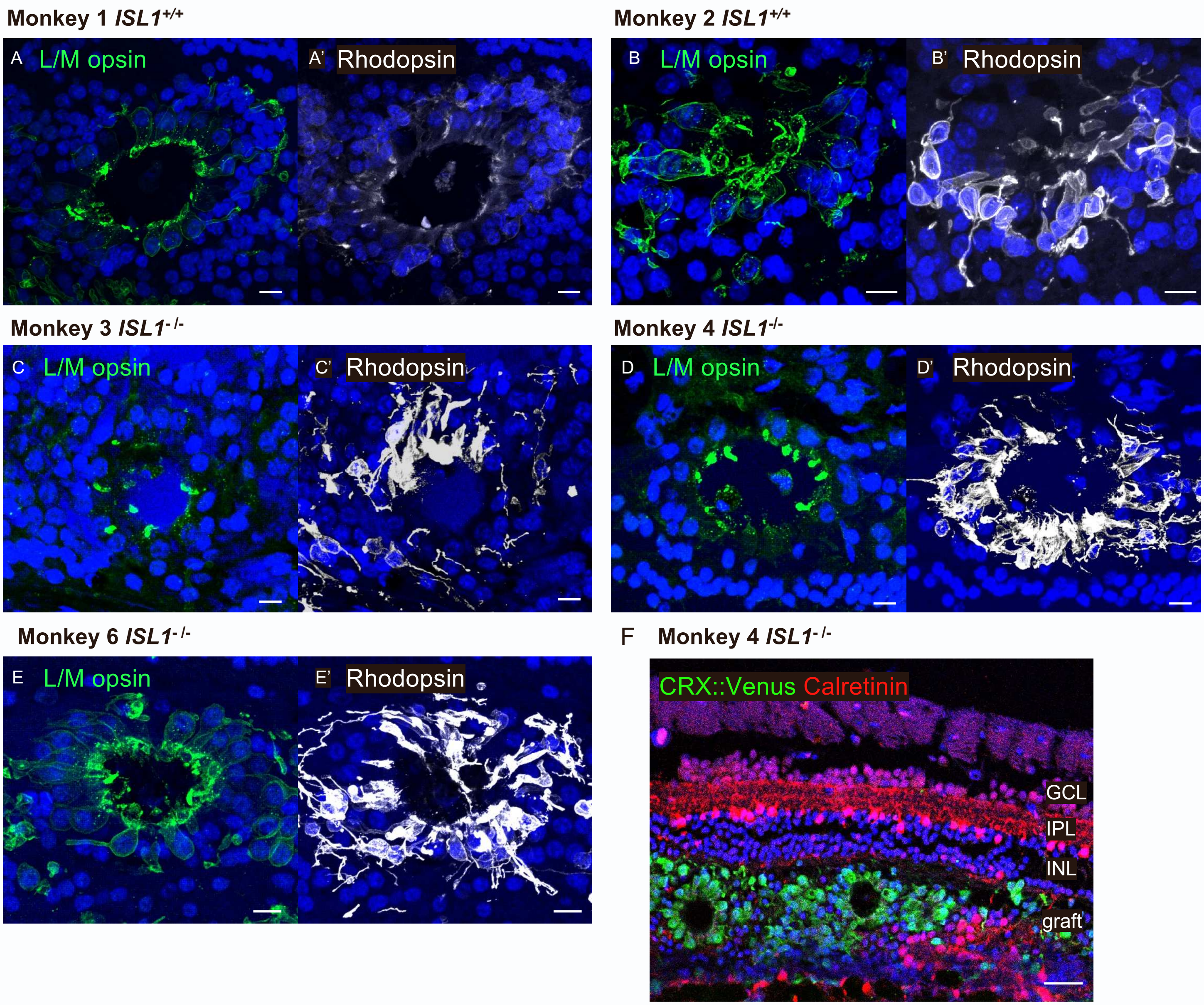

Figure S11. **Histological evaluation of graft maturation and inner plexiform layer integrity.** (A–E') Immunostaining of transplanted monkey retinas showing L/M-opsin and rhodopsin expression in photoreceptor rosettes in *ISL1*$^{+/+}$ and *ISL1*$^{-/-}$ grafts post-transplantation, indicating the differentiation of cone and rod photoreceptors. Scale bars: 10 µm. (F) Representative image of Monkey4 , showing host retinal integrity, as indicated by Calretinin immunopositivity in the host inner plexiform layer in the engrafted area with CRX::Venus-positive graft. Scale bars: 50 µm

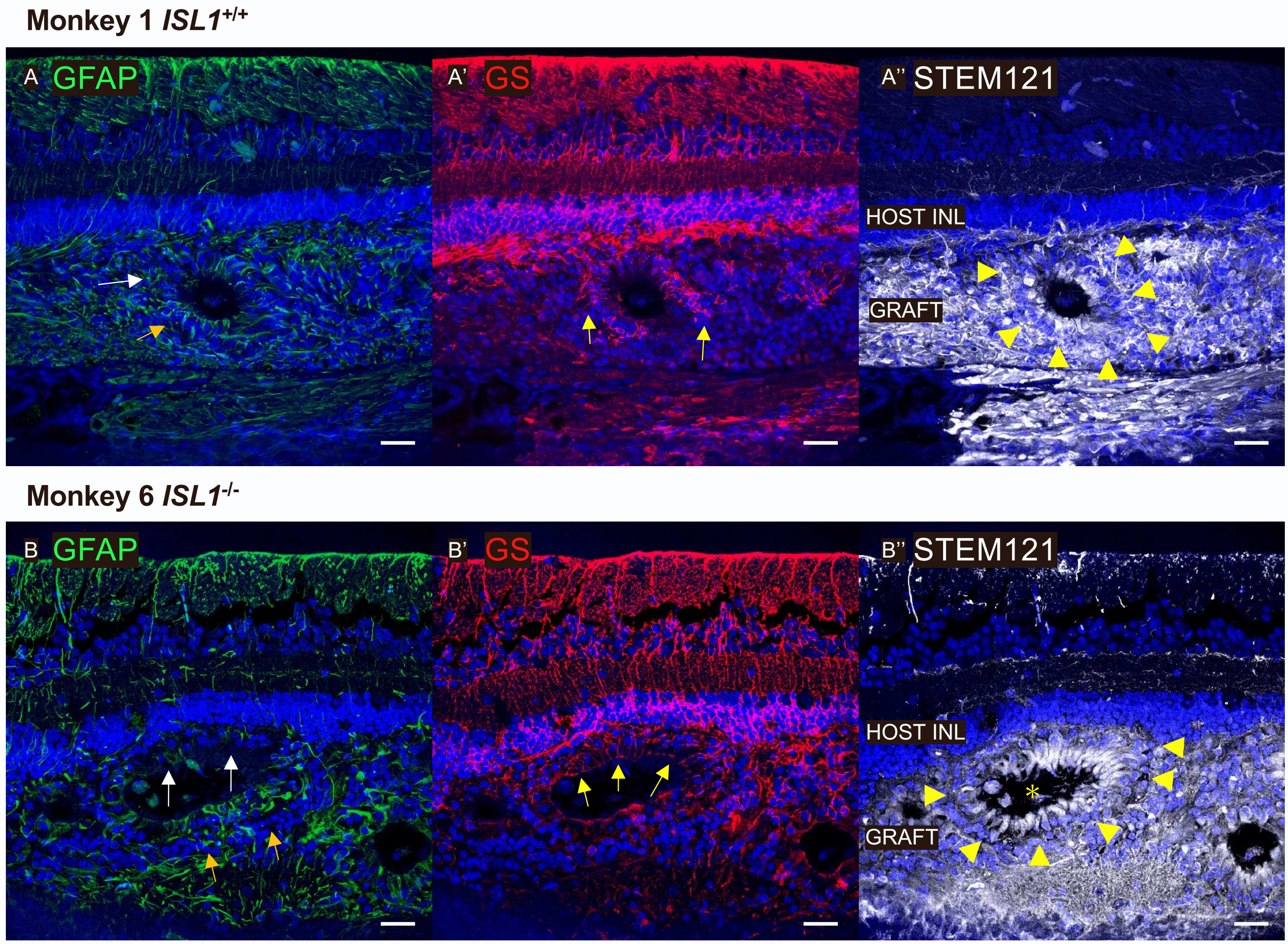

Figure S12. Host and graft Müller cells in *ISL1*$^{+/+}$ and *ISL1*$^{-/-}$ grafts after transplantation. (A–B'') Representative images of host and graft Müller cells from Monkey 1 (*ISL1*$^{+/+}$ grafts; A–A'') and Monkey 6 (*ISL1*$^{-/-}$ graft; B–B''). GS-positive Muller cells were consistently present alongside graft photoreceptors within rosettes, potentially supporting photoreceptor alignment (yellow arrows in A' and B') Scale bars: 30 µm . GFAP was moderately activated in the host retina but did not form a gliotic barrier between the host and graft. Within the graft, GFAP was elevated on the RPE-proximal side of rosettes (orange arrows) compared to the host-proximal side (white arrows in A and B). STEM121 labels grafted ROs, including photoreceptor rosettes (yellow arrow heads in A'' and B'') in which photoreceptors sometimes exhibited inner/outer segment-like morphology (asterisk). GFAP, glial fibrillary acidic protein; GS, glutamine synthetase; RPE, retinal pigment epithelium; INL, inner nuclear layer; ONL, outer nuclear layer. Graft-derived cells were distinguished by STEM121.

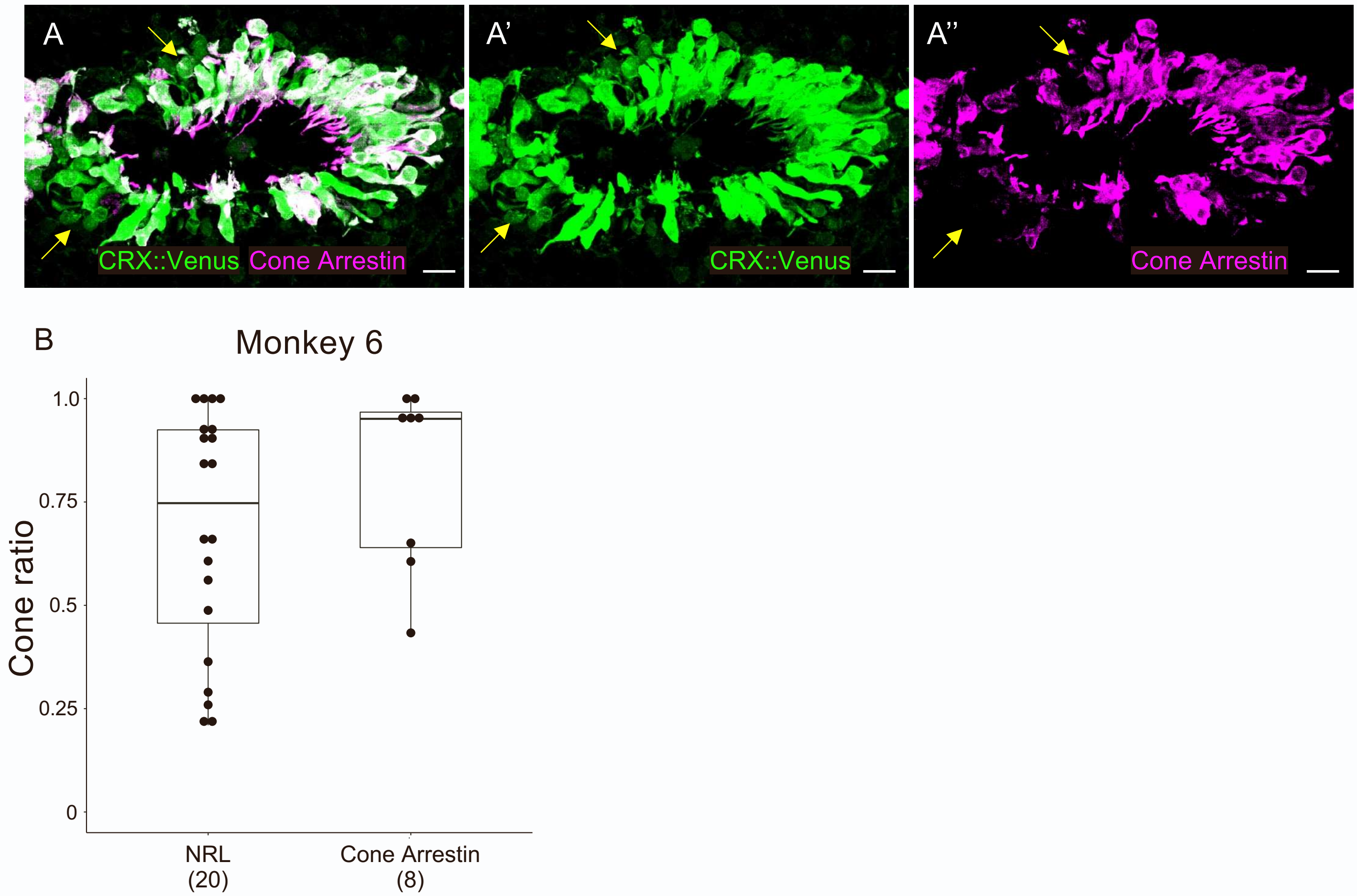

Figure S13. The cone ratio of Monkey 6 using Cone Arrestin. (A–A'') Representative image for CRX::Venus and Cone Arrestin immunostaining in the graft (monkey 6), distinguishing cone from rod photoreceptors. Graft photoreceptors were identified within CRX::Venus-positive rosettes in the graft area. Scale bars: 15 µm (B) Quantification of cone proportion per rosette in monkey 6 using NRL-based (CRX+ NRL-/CRX+) or Cone Arrestin-based (CRX+ Cone Arrestin+/CRX+) classification (NRL: n = 20 rosettes; Cone Arrestin: n = 8 rosettes). Yellow arrows indicate rod photoreceptors (CRX::Venus-positive cells; Cone Arrestin-negative cells)

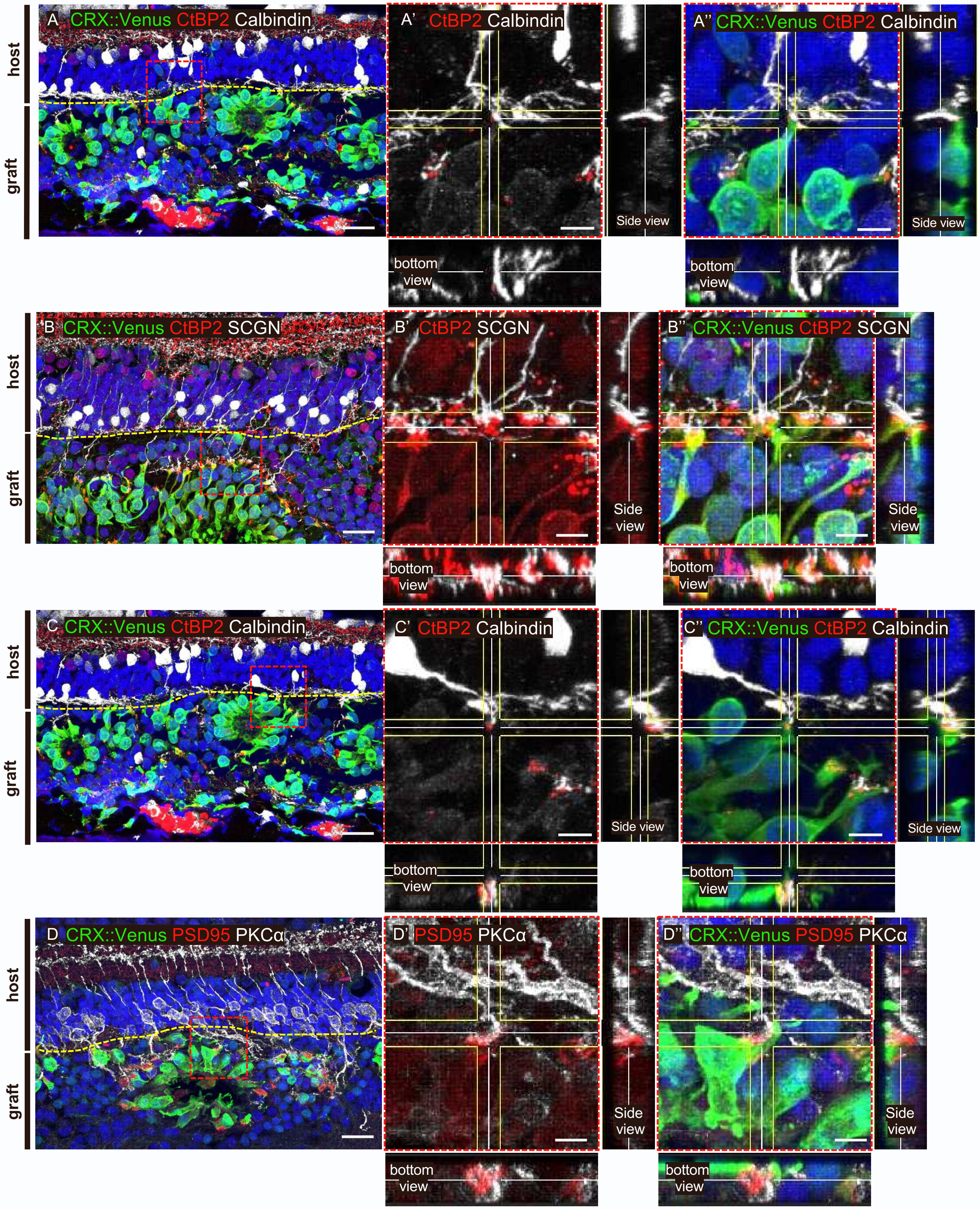

host
graft
A CRX::Venus CtBP2 Calbindin
A' CtBP2 Calbindin
Side view
bottom view
A'' CRX::Venus CtBP2 Calbindin
Side view
bottom view
B CRX::Venus CtBP2 SCGN
B' CtBP2 SCGN
Side view
bottom view
B'' CRX::Venus CtBP2 SCGN
Side view
bottom view
C CRX::Venus CtBP2 Calbindin
C' CtBP2 Calbindin
Side view
bottom view
C'' CRX::Venus CtBP2 Calbindin
Side view
bottom view
D CRX::Venus PSD95 PKCα
D' PSD95 PKCα
Side view
bottom view
D'' CRX::Venus PSD95 PKCα
Side view
bottom view

Figure S14. Host-graft synaptic formation of transplanted photoreceptors.
(A–B'') Presynaptic marker CtBP2 was localized on the margin of the CRX::Venus (graft photoreceptors) and the dendrite tips of Calbindin-positive (A–A'') and SCGN-positive (B-B'') BCs. Scale bars: 15 µm (A, B), 4 µm (A', A'', B, B'') (C–C'') Presynaptic marker CtBP2 was localized on the margin of the CRX::Venus (graft photoreceptors) and the dendrite tips of Calbindin-positive horizontal cells. Scale bars: 15 µm (C), 4 µm (C', C'') (D–D'') Presynaptic marker PSD95 was localized on the margin of graft photoreceptors identified within CRX::Venus-positive rosettes in the graft area, contacting the dendritic tips of host PKCα-positive BCs, identified by their anatomical location within the host INL. PKCα-positive bipolar dendrites were considered exclusively of host origin, as *ISL1*$^{-/-}$ grafts lack ON-bipolar cells. Scale bars: 15 µm (D), 4 µm (D', D'')

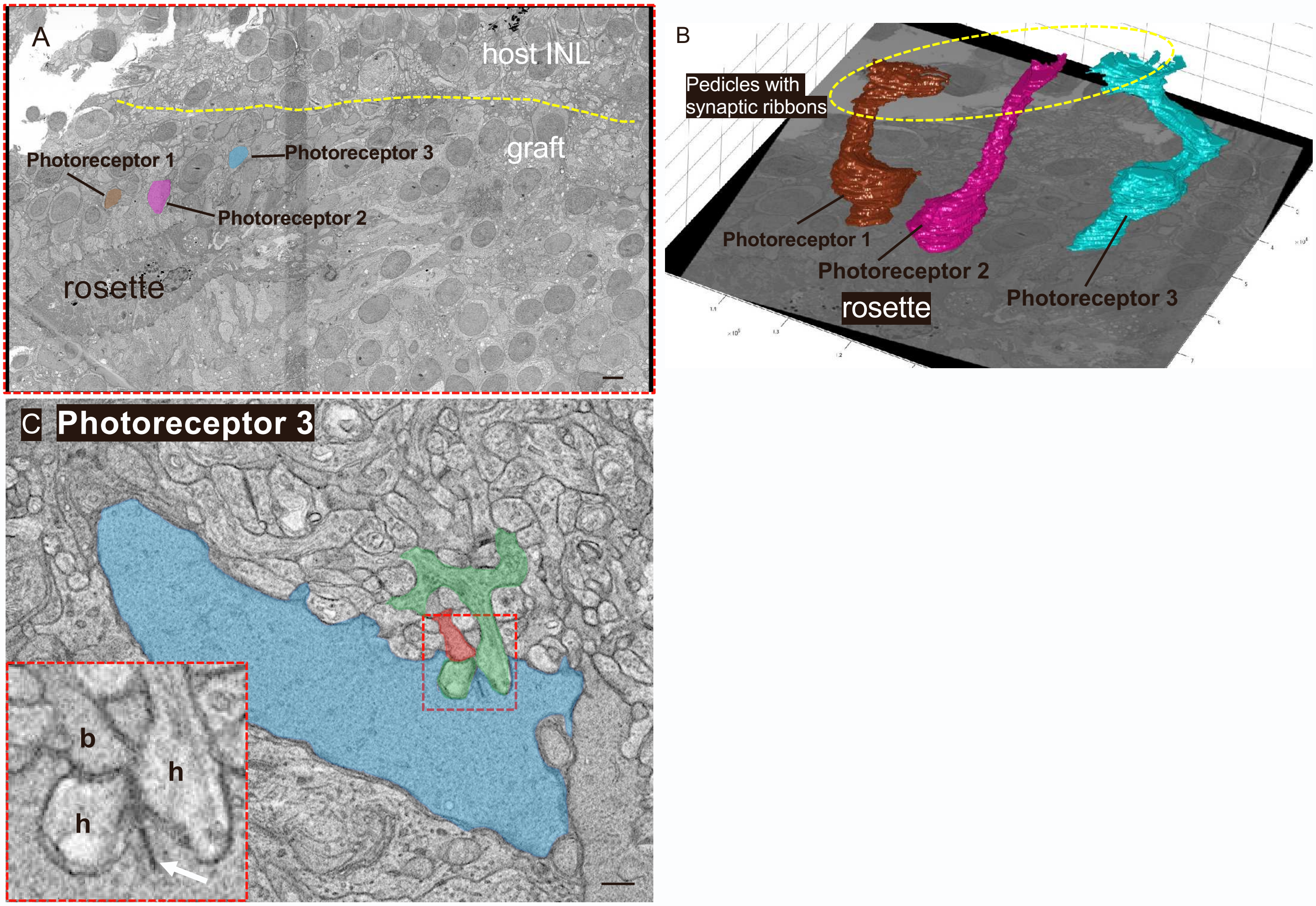

Figure S15. Synaptic connections between host BCs and graft photoreceptors observed in EM. (A) Enlarged view of the area within the red dotted rectangle in (Figure 7C), showing the rosette that includes each traced photoreceptor. Graft photoreceptors were identified within CRX::Venus-positive rosettes by first acquiring z-stack fluorescence images from an original section, from which serial electron microscopy images were obtained with subsequent correlation of the two imaging modalities. Photoreceptor 3: blue (C) scale bars: 5 µm (B) 3D reconstructed image of graft photoreceptors extending from the rosette into the host INL; three photoreceptors were traced. (C) EM images of the axon terminals of individual photoreceptors. white arrows showed synapse ribbon. b, bipolar cell; h, likely horizontal cell. scale bars: 500 nm

Table S1. Summary of transplanted monkey eyes

Monkey number	Monkey 1	Monkey 2	Monkey 3	Monkey 4	Monkey 5	Monkey 6
**Age at transplantation (year)**	6	3	3	3	3	9
**Transplanted eye**	Right	Right	Left	Right	Right	Right
**Organoid genotype**	Wild type	Wild type	*ISL1*$^{-/-}$	*ISL1*$^{-/-}$	*ISL1*$^{-/-}$	*ISL1*$^{-/-}$
**Differentiation day of RO at transplantation**	56	63	63	59	63	64
**Days post-laser at transplantation**	29	43	18	43	47	28
**Postsurgical events**	Rejection (resolved with steroid at 1.5 months)	Rejection (17 months)	-	Postoperative Endophthalmitis (3 weeks)	Cataract (1 months)	-
**Graft loss**	+	+	-	+	-	-
**Sampling time for histology**	14 months (427 days)	17 months (509 days)	14 months (413 days)	9 months (286 days)	13 months (402 days)	25 months (769 days)

Table S2. Immunohistochemical summary of bipolar cells

Primary Antibody	Type of Bipolar cells				
**EAAT2**	**Diffuse Bipolar 1**	**Diffuse Bipolar 2**	**Diffuse Bipolar 3a**	**Diffuse Bipolar 3b**	**Flat midget Bipolar**
**Calbindin**	**Diffuse Bipolar 3a**	<u>Diffuse Bipolar 6</u>	Horizontal cell		
**SCGN**	**Diffuse Bipolar 1**	<u>Diffuse Bipolar 6</u>			
**PKC α**	<u>Diffuse Bipolar 4</u>	<u>Diffuse Bipolar 5</u>	<u>Rod bipolar</u>		

ON-bipolar cells are indicated by solid lines, whereas OFF-bipolar cells are indicated in bold. Cell-type markers for the primary antibodies were selected based on previous studies.[19,22]

Table S3. Summary of primary antibodies		
Goat polyclonal anti-BRN3	Santa Cruz biotechnology	Cat#sc-6026 RRID: AB_673441
Rabbit polyclonal anti-Calbindin	Millipore	Cat#ABN2192 RRID: AB_2935805
Mouse monoclonal anti-Calretinin	Millipore	Cat#MAB1568 RRID: AB_94259
Rabbit polyclonal anti-CD3	Abcam	Cat#ab5690 RRID: AB_305055
Mouse monoclonal anti-CHX10	Santa Cruz biotechnology	Cat#sc-365519 RRID: AB_10842442
Mouse monoclonal anti-Arrestin C	Sigma-Aldrich	Cat#MABN2636 RRID:AB_2935804
Mouse monoclonal anti-CtBP2	BD	Cat#612044 RRID: AB_399431
Rabbit polyclonal anti-EAAT2	Proteintech	Cat#22515-1-AP RRID: AB_2879112
Rabbit polyclonal anti-GFAP	DAKO	Cat#Z0334 RRID: AB_ 10013382
Rabbit polyclonal anti-GFP	Abcam	Cat#ab290 RRID: AB_ 2313768
Goat polyclonal anti-Venus	ORIGENE	Cat#AB2166-100 RRID: AB_2895457
Mouse monoclonal anti-GluK1	Santa Cruz biotechnology	Cat#sc-393420 RRID: AB_2716684
Mouse monoclonal anti-GS	Millipore	Cat#MAB302 RRID: AB_ 2110656
Rabbit polyclonal Anti-GNG13	Abcam	Cat#ab126562 RRID:
Rabbit polyclonal anti-Iba1	Wako	Cat#019-19741 RRID: AB_839504
Rabbit polyclonal anti-mGluR6	Novus Biologicals	Cat#NLS4655 RRID: AB_343723
Mouse Monoclonal anti-MHC classII	DAKO	Cat#M0775 RRID: AB_2313661

Goat polyclonal anti-NRL	R&D	Cat#AF2945 RRID: AB_2155098
Goat polyclonal anti-OPN1SW	Santa Cruz biotechnology	Cat#sc-14363 RRID: AB_2158332
Rabbit polyclonal anti-Opsin Re+G	Sigma-Aldrich	Cat#AB5405 RRID: AB_177456
Rabbit polyclonal anti-Opsin Blue	Millipore	Cat#AB5407 RRID: AB_304867
Mouse monoclonal anti-PAX6	BD Biosciences	Cat#BD561462 RRID: AB_10715442
Rabbit polyclonal anti-Peripherin-2	Proteintech	Cat#18109-1-AP RRID: AB_10665364
Mouse monoclonal anti-PKCα	Sigma-Aldrich	Cat#P5704 RRID: AB_477375
Mouse monoclonal anti-PSD95	Biolegend	Cat#810401 RRID: AB_2564750
Rabbit polyclonal anti-Recoverin	Proteintech	Cat#10073-1-AP RRID: AB_2178005 _
Mouse monoclonal anti-Rhodopsin	Sigma-Aldrich	Cat#O4886 RRID: AB_260838
Rabbit polyclonal anti-RPE65	Takara	Cat#AS2352
Mouse monoclonal Anti-RXR γ	Santa Cruz biotechnology	Cat#sc-365252 RRID: AB_10850062
Sheep polyclonal anti-SCGN	BioVendor R&D	Cat#RD184120100 RRID: AB_2034062
Mouse monoclonal anti-STEM121	Takara	Cat#Y40410 RRID: AB_ 2801314

Video S1. 3D reconstruction of host-graft synaptic connection by SBFSEM. This video shows a host ON-bipolar cell (yellow) and a host OFF-bipolar cell (orange) forming synaptic contacts with a grafted cone photoreceptor (pink).
